# Primary liver cancer in the UK: Incidence, incidence-based mortality, and survival by subtype, sex, and nation

**DOI:** 10.1016/j.jhepr.2021.100232

**Published:** 2021-01-19

**Authors:** Anya Burton, Daniela Tataru, Robert J. Driver, Thomas G. Bird, Dyfed Huws, David Wallace, Timothy J.S. Cross, Ian A. Rowe, Graeme Alexander, Aileen Marshall, HCC-UK/BASL/NCRAS Partnership Steering Group, Anya Burton, Aileen Marshall, Graeme Alexander, Ian Rowe, Robert J. Driver, Vinay Kumar, Tim Cross, Katherine Cullen, Rhys Pockett, Tom Bird, Dyfed W. Huws, Anna Gavin, Daniela Tataru, Lizz Paley, David Wallace, Guruprasad Aithal

**Affiliations:** 1 HCC-UK/British Association for the Study of the Liver (BASL), Lichfield, UK; 2 National Cancer Registration and Analysis Service, National Disease Registration Service, Public Health England, London, UK; 3 Leeds Institute for Medical Research at St. James’s, University of Leeds, Leeds, UK; 4 Cancer Research UK Beatson Institute, Glasgow, UK; 5 Institute of Cancer Sciences, University of Glasgow, Glasgow, UK; 6 MRC Centre for Inflammation Research, University of Edinburgh, Edinburgh, UK; 7 Welsh Cancer Intelligence and Surveillance Unit, Knowledge Directorate, Public Health Wales, Cardiff, UK; 8 Department of Health Services Research and Policy, London School of Hygiene and Tropical Medicine, London, UK; 9 Institute of Translational Medicine, The University of Liverpool, Liverpool, UK; 10 Leeds Institute for Medical Research, University of Leeds, Leeds, UK; 11 Leeds Liver Unit, St. James's University Hospital, Leeds, UK; 12 UCL Institute for Liver and Digestive Health, Royal Free Hospital, London, UK; 13 Sheila Sherlock Liver Centre, The Royal Free Hospital, London, UK

**Keywords:** Primary liver cancer, Hepatocellular carcinoma, Intrahepatic cholangiocarcinoma, Incidence, Mortality, Survival, AAPC, average annual percentage change, APC, annual percentage change, ASMR, age-standardised mortality rate, ASR, age-standardised incidence rate, BASL, British Association for the Study of the Liver, DAA, direct-acting antivirals, DCO, death certificate only, HCC, hepatocellular carcinoma HCV, hepatitis C virus, ICCA, intrahepatic cholangiocarcinoma, ICD-10, International Classification of Diseases 10th Edition, ICD-O, International Classification of Diseases for Oncology, NAFLD, non-alcoholic fatty liver disease, NCRAS, National Cancer Registration and Analysis Service, NI, Northern Ireland, PLC, primary liver cancer

## Abstract

**Background & Aims:**

The incidence of primary liver cancer (PLC) is increasing in Western Europe. To understand trends over time and the current burden in the UK, a detailed analysis of the epidemiology of PLC and its subtypes was conducted.

**Methods:**

Data on PLCs diagnosed during 1997–2017 were obtained from population-based, nationwide registries in the UK. European age-standardised incidence (ASR) and incidence-based mortality rates (ASMR) per 100,000 person-years were calculated overall and by sex and UK-nation. Annual percentage change in rates was estimated using Joinpoint regression. One-, 2-, and 5-year age-standardised net survival was estimated.

**Results:**

A total of 82,024 PLCs were diagnosed. Both hepatocellular carcinoma (HCC) incidence and mortality rates trebled (ASR 1.8–5.5 per 100,000, ASMR 1.3–4.0). The rate of increase appeared to plateau around 2014/2015. Scottish men consistently had the highest HCC incidence rates. PLC survival increased, driven by a substantial increase in the proportion that are HCC (as prognosis is better than other PLCs) and in HCC survival (change in 1-year survival 24–47%). Intrahepatic cholangiocarcinoma was the most common PLC in women and 1-year survival improved from 22.6% to 30.5%.

**Conclusions:**

PLC incidence has been increasing rapidly but, as most risk factors are modifiable, it is largely a preventable cancer. This rate of increase has slowed in recent years, possibly attributable to effective treatment for hepatitis C. As other risk factors such as obesity and diabetes remain prevalent in the UK, it is unlikely the considerable burden of this disease will abate. While improvements in survival have been made, over half of patients are not alive after 1 year, therefore further progress in prevention, early detection, and treatment innovation are needed.

**Lay summary:**

Many more people are getting liver cancer, particularly the subtype hepatocellular carcinoma, than 20 years ago. Men in Scotland are most likely to get liver cancer and to die from it. Survival after liver cancer diagnosis is getting longer but still less than half are alive after 1 year.

## Highlights

• Primary liver cancer incidence and mortality rates have rapidly increased between 1997 and 2014.
• The rate of increase in hepatocellular carcinoma incidence rates has plateaued since around 2014.
• Hepatocellular carcinoma incidence and mortality rates were highest in Scottish men.
• Survival has improved, particularly for hepatocellular carcinoma, but remains poor.

## Graphical abstract

**Figure:**
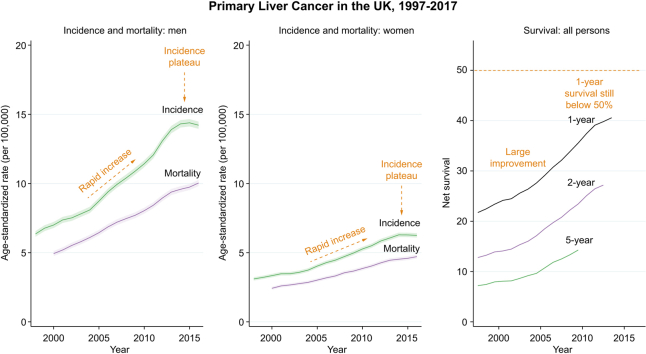

## Introduction

Primary liver cancer (PLC) is the fourth leading cause of cancer-related mortality worldwide, causing an estimated 800,000 deaths in 2018.^1^ Incidence rates have been rising in many countries globally but particularly rapidly in Western Europe, Australasia, and North America.^2^ In the UK, PLC has been amongst the cancers with the most rapid rate of growth in both incidence and mortality in recent decades, and is projected to be the cancer with the highest average annual increase over the next 15 years.^3^ Survival is particularly poor for this cancer with 5-year relative survival estimates below 10%.^4^

The main PLC subtype, hepatocellular carcinoma (HCC), is an epithelial tumour which shows hepatocytic differentiation and primarily occurs against a backdrop of cirrhosis, caused by factors such as chronic viral hepatitis, alcohol consumption, and non-alcoholic fatty liver disease. The second most common subtype, intrahepatic cholangiocarcinoma (ICCA), is an epithelial tumour showing biliary differentiation in the liver, and often occurs in patients with no known risk factors. A small proportion of PLCs have mixed features of both HCC and ICCA. There are also rare subtypes such as hepatoblastoma, Kupffer cell sarcoma, and other carcinomas and sarcomas. The distribution of risk factors, and subsequently liver cancer rates, change with time owing to factors such as changing behaviours, large-scale public health initiatives such as vaccination and other forms of infection prevention, and innovation in treatment of underlying liver diseases. Understanding the current burden of liver cancer and trends over time can quantify the public health concern, identify the need for public health interventions aimed at prevention and indicate whether it is necessary to scale-up facilities and training to meet increasing demand. Further examination by subtype is important as the causes, treatment, and outcomes are quite different.

The HCC-UK BASL/National Cancer Registration and Analysis Service (NCRAS) partnership was formed to facilitate a wide programme of research relating to HCC, primarily using the detailed data available within the NCRAS on all cancer patients resident in England. Here, within the framework of this partnership and including corresponding data from the Welsh Cancer Intelligence and Surveillance Unit, the Northern Ireland Cancer Registry, and the Scottish Cancer Registry, the incidence, incidence-based mortality and survival trends in PLC and its subtypes in the UK and each constituent nation between 1997 and 2017 were examined.

## Patients and methods

### Data sources

Patient-level data on PLC tumours, defined using the 10th edition of the International Classification of Diseases (ICD-10) code C22, diagnosed between 1997 and 2017 were obtained from the population-based, nationwide registries in Wales, Scotland, Northern Ireland (NI), and England. Patient-level data collected included ICD-10 code, second edition of the ICD for Oncology (ICD-O-2) morphology code, diagnosis year, death year, days from diagnosis to death, or latest vital status date (31 December 2017 for Wales, 31 December 2018 for NI and Scotland, and 06 February 2019 for England), vital status (alive/dead/emigrated), age at diagnosis (5-year age bands), sex, underlying cause of death (ICD-9 or ICD-10 code) and basis of diagnosis. Only those tumours with a malignant ICD-O-2 behaviour code were included.

For incidence, all new diagnoses in the period 1997–2017 were included. For mortality and survival calculations, the first liver tumour only was included. Records with a date of death before date of diagnosis (N = 27) and those with the ICD-O-2 morphology code 8162, Klatskin tumour (perihilar CCA), were excluded (N = 298). For mortality, those with missing cause of death were excluded (N = 913, 0.9% of deaths). For survival analyses those that embarked (as no last vital status date, N = 170), registrations based on death certificate only (and autopsy diagnoses only in the Scottish data) (N = 3,067) and those aged <15 years (N = 493) were excluded. ICD-10 codes and ICD-O-2 morphology codes were used to define subtypes of PLC as outlined in the Supplementary information and Table S1.

Mid-year population data for 1997–2017 were obtained from the Office for National Statistics.^5^ Life tables in 5-year age bands, by sex and calendar year of death for the UK overall and for England and Wales, Scotland, and NI separately were obtained from the Human Mortality Database^6^ for 1997–2016. For estimation of survival in 2017, 2016 life tables were used.

### Statistical analysis

#### Incidence

European age-standardised^7^ incidence (ASR) and incidence-based mortality (ASMR) rates per 100,000 person-years and Dobson confidence intervals were calculated using the *distrate* command in Stata.^8^ Estimates from 3-year rolling cohorts were used for graph smoothing.

#### Mortality

Standard mortality, which uses death certificate coding to count the number of deaths from a particular cause in a population per year, results in mortality rates that are higher than incidence rates for ICCA (Fig. S1). This may, in part, be as a result of over-recording of ICCA on death certificates, which use the ICD-10 system, as CCA is coded as intrahepatic (C22.1) if no tumour location is given. To resolve this and to more accurately identify PLC deaths by subtype, an incidence-based mortality approach was taken where the detailed cancer registry diagnostic data was used to identify all those with primary liver tumours and classify the subtype, and the death certificate cause of death used to identify the cancer-related deaths in these patients. Both deaths from liver cancer (C22) and extrahepatic cholangiocarcinoma (C24.0) were included to ensure any deaths coded as intrahepatic CCA in the registry and extrahepatic CCA on the death certificate were captured. Within each registry cancer registration officers review data from multiple sources to identify new cancer cases and classify tumours according to the ICD-O-2 system which is a cancer-specific coding system that assigns a specific site, morphology, and behaviour code to each tumour.^9^ As only diagnoses from 1997 were available, mortality rates are presented for 1999 onwards. A 2-year ‘run-off’ period was identified as being sufficient in sensitivity analyses on English data including diagnoses pre-1997 and those from 1997 only (Fig. S1).

Joinpoint regression modelling was used to model trends in incidence and incidence-based mortality rates and identify timepoints when significant changes occurred, constrained to a maximum of 3 joinpoints (Joinpoint Regression Program, Version 4.7.0, Statistical Methodology and Applications Branch, Surveillance Research Program, National Cancer Institute). Annual percentage change (APC) was calculated for each segment, and average APC (AAPC) for the whole period.

#### Survival

One-, 2-, and 5-year age-standardised net survival was calculated for 2- and 5-year cohorts using the *strs* command in Stata and the cohort approach.^10^ Survival was only estimated for years with complete follow-up. The Brenner method of age-standardisation was applied.^11^ One day was added to survival times of 0 (*i.e.* diagnosis and death on the same day). Survival estimates were supressed if there were no deaths or data in at least 1 age band, less than 10 in a group, the standard error was greater than 0.2, 2-year survival was greater than 1-year survival, and/or the follow-up time was insufficient in a cohort. Estimates from 5-year rolling cohorts were used for graph smoothing.

All analyses were conducted using Stata version 15.1 StataCorp, 2017, Stata Statistical Software: Release 15 (StataCorp, College Station, TX, USA).

## Results

### Overview

Overall, in the UK 82,024 PLCs were diagnosed between 1997 and 2017 and 58,348 individuals died from their liver cancer between 1999 and 2017 (Table 1). The age-standardised incidence rate for the whole period was 7.3 per 100,000. Ninety percent of PLCs occurred in those aged 50 years or older and ICCA patients were slightly older on average than HCC patients (Fig. S2). The age distribution was wide in patients with other liver tumours, reflecting the diverse tumours in this group. Over 60% of PLCs were in men (77% of HCCs, 47% of ICCAs, and 58% of other liver tumours) and PLC incidence rates were over twice as high in men than in women (10.5 *vs.* 4.7 per 100,000, respectively). A total of 50.5% of PLCs were HCCs (ASR 3.7 per 100,000), 37.1% were ICCAs (ASR 2.7 per 100,000), and 12.4% other or unspecified liver cancers (ASR 0.9 per 100,000). These proportions varied by nation; in Scotland and NI approximately 60% of PLCs were HCC and 30% ICCA, whereas in England and Wales approximately 50% were HCC and 40% ICCA (*p* for from chi-squared test for difference between nations <0.001). Scotland had the highest PLC incidence rate (ASR 8.9 per 100,000) and NI the lowest (ASR 6.3 per 100,000). The overall incidence-based age-standardised mortality rate was 5.2 per 100,000. A total of 47.9% were deaths from HCCs, 42.5% from ICCA, and 9.5% from other and unspecified liver cancers. In general, mortality rates followed similar patterns to incidence (higher in men than in women and highest in Scotland and lowest in NI). However, the overall mortality rate for ICCA (2.2 per 100,000) was nearly as high as for HCC (2.5 per 100,000).

**Table 1:** Primary liver cancer age-standardised incidence and incidence-based mortality rates 1997–2017, by nation, sex, and subtype.

	Incidence							Incidence-based mortality						
	N cases	Crude rate per 100,000	ASR per 100,000	95% CI	Time period	APC	95% CI	N deaths	Crude rate per 100,000	ASMR per 100,000	95% CI	Time period	APC	95% CI
Overall														
	82,024	6.3	7.3	(7.2–7.3)	AAPC 1997–2017	4.1^∗^	(3.4–4.7)	58,348	4.5	5.2	(5.2-5.2)	AAPC 1999-2017	4.5∗	(4.1–4.8)
					1997–2003	3.6^∗^	(2.0–5.1)					1999-2013	5.1∗	(4.8–5.3)
					2003–2014	5.9^∗^	(5.3–6.4)					2013-2017	2.4∗	(1.1–3.8)
					2014–2017	-1.3	(-4.1–1.5)							
Sex														
Men	52,258	8.2	10.5	(10.4–10.6)	AAPC 1997–2017	4.5^∗^	(3.8–5.2)	36,373	5.7	7.4	(7.3-7.5)	AAPC 1999-2017	4.4∗	(3.9–4.8)
					1997–2014	5.4^∗^	(5.0–5.8)					1999-2013	4.9∗	(4.6–5.3)
					2014–2017	-0.6	(-4.6–3.5)					2013-2017	2.5∗	(0.8–4.3)
Women	29,766	4.5	4.7	(4.6–4.7)	AAPC 1997–2017	3.6^∗^	(2.9–4.2)	21,975	3.3	3.5	(3.4-3.5)	AAPC 1999-2017	4.2∗	(3.7–4.8)
					1997–2003	2.6^∗^	(0.9–4.2)					1999-2013	4.8∗	(4.4–5.3)
					2003–2013	5.7^∗^	(5.0–6.4)					2013-2017	2.2	(0.0–4.4)
					2013–2017	-0.1	(-2.1–1.9)							
Country														
England	67,177	6.2	7.1	(7.1–7.2)	AAPC 1997–2017	4.5^∗^	(3.8–5.2)	47,840	4.4	5.1	(5.1-5.1)	AAPC 1999-2017	4.5∗	(4.1–4.9)
					1997–2014	5.4^∗^	(5.0–5.8)					1999-2012	5.3∗	(4.9–5.7)
					2014–2017	-0.4	(-4.4–3.6)					2012-2017	2.6∗	(1.4–3.7)
Wales	4,502	7.1	7.5	(7.3–7.8)	AAPC 1997–2017	3.2^∗^	(1.0–5.5)	3,232	5.1	5.4	(5.2-5.6)	AAPC 1999-2017	4.0∗	(3.0–4.9)
					1997–2015	4.7^∗^	(3.8–5.7)							
					2015–2017	-9.2	(-27.5–13.7)							
Scotland	8,572	7.9	8.9	(8.7–9.1)	AAPC 1997–2017	3.7^∗^	(1.4–6.2)	6,087	5.6	6.3	(6.2-6.5)	AAPC 1999-2017	4.7∗	(3.9–5.5)
					1997–2010	3.5^∗^	(2.4–4.6)							
					2010–2013	12.4	(-3.6–31)							
					2013–2017	-1.5	(-5.7–2.8)							
NI	1,773	4.8	6.3	(6.0–6.6)	AAPC 1997–2017	4.5^∗^	(3.3–5.8)	1,189	3.2	4.3	(4-4.5)	AAPC 1999-2017	5.6∗	(3.9–7.4)
Subtype														
HCC	41,454	3.2	3.7	(3.6–3.7)	AAPC 1997–2017	5.9^∗^	(5.1–6.7)	27,970	2.2	2.5	(2.5-2.5)	AAPC 1999-2017	5.8∗	(5.4–6.1)
					1997–2015	6.8^∗^	(6.4–7.2)							
					2015–2017	-1.5	(-8.7–6.3)							
ICCA	30,402	2.4	2.7	(2.7–2.8)	AAPC 1997–2017	3.4^∗^	(2.8–4.1)	24,813	1.9	2.2	(2.2-2.3)	AAPC 1999-2017	3.8∗	(3.3–4.3)
					1997–2009	4.4^∗^	(3.6–5.3)					1999-2009	5.0∗	(4.3–5.8)
					2009–2017	2.0^∗^	(0.8–3.2)					2009-2017	2.2∗	(1.4–3.0)
Other	10,165	0.8	0.9	(0.9–0.9)	AAPC 1997–2017	0.3	(-1.9–2.6)	5,563	0.4	0.5	(0.5-0.5)	AAPC 1999-2017	0.1	(-4.3–4.7)
					1997–2005	-2.5	(-5.8–0.9)					1999-2001	-16.3	(-36.2–9.7)
					2005–2013	9.7^∗^	(5.9–13.7)					2001-2011	3.3∗	(0.8–5.9)
					2013–2017	-11.0^∗^	(-17.3–4.2)					2011-2014	13.8	(-8.8–41.9)
												2014-2017	-10.9∗	(-20.2–0.5)

AAPC from best-fitting model given. If model has >1 joinpoint, APC for each time-period is also given. ∗The APC or AAPC is significantly different from zero at the alpha = 0.05 level. AAPC, average annual percentage change; APC, annual percentage change; ASR, age-standardised incidence rate; ASMR, age-standardised mortality rate; HCC, hepatocellular carcinoma; ICCA, intrahepatic cholangiocarcinoma; NI, Northern Ireland.

### Trends by subtype, nation, and sex

#### PLC

**Fig. 1:**
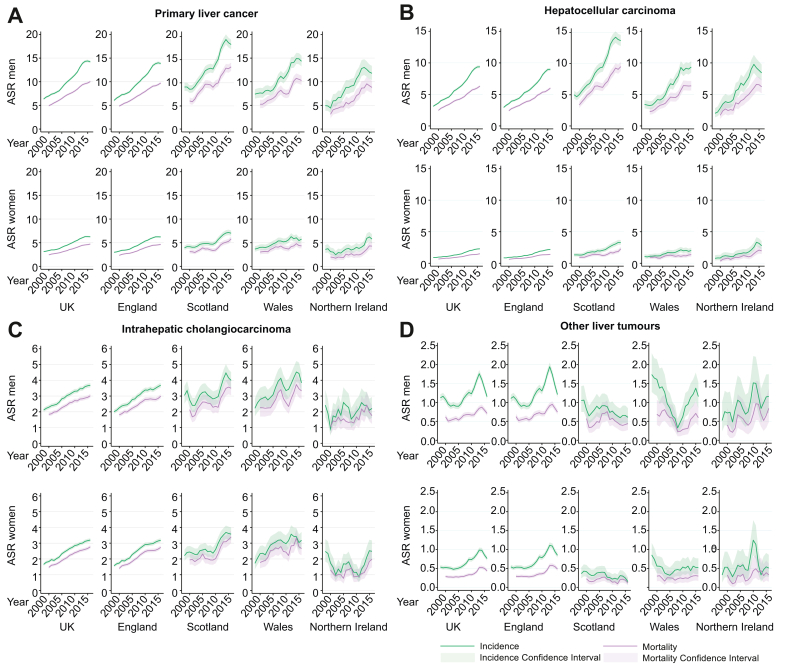
Age-standardised incidence and incidence-based mortality. (A) Primary liver cancer; (B) hepatocellular carcinoma; (C) intrahepatic cholangiocarcinoma; (D) other and unspecified liver tumours. Estimates from 3-year rolling cohorts. ASR, age-standardised incidence rate per 100,000.

In the UK, PLC incidence increased from 4.4 per 100,000 in 1997 to 9.6 in 2017, an AAPC of 4.1% (95% CI 3.4–4.7; Fig. S3 and Table 1). The Joinpoint trend analysis indicated the fastest increase in PLC incidence in the UK was between the years 2003 and 2014 (APC 5.9% per year, 95% CI 5.3–6.4) and after this it plateaued (-1.3% per year, 95% CI -4.1 to 1.5). The pattern of a rapid increase in incidence rates then a plateau was seen in both men and women and across nations (Fig. 1A), although in NI data were sparse and therefore trends less clear. The highest PLC incidence rates were seen in Scottish men, reaching a peak of 20.1 per 100,000 in 2013 (Table S2). The mortality rate changed from 3.3 per 100,000 in 1999 to 7.5 in 2017, increasing 5.1% per year (95% CI 4.8–5.3) until 2013 and then 2.4% per year (95% CI 1.1–3.8), not reaching plateau. As with incidence, the highest mortality rates were seen in Scottish men (ASMR in 2017: 13.9 per 100,000).

#### HCC

HCC incidence increased from 1.8 to 5.5 per 100,000, an AAPC increase of 5.9% per year. The fastest rate of growth was from 1997 to 2015 (APC 6.8% per year, 95% CI 6.4–7.2 UK, all persons), after which it plateaued (APC 2015–2017 -1.5%, 95% CI -8.7 to 6.3; Table 1). When further analysed by sex, the plateau was seen mainly in men (Table 2 and Fig. 1B), in whom rates overall were over 4 times higher than in women. For women, the best-fitting model had no joinpoints and incidence rates rose 5.7% per year from 1997 to 2017 (data not shown). The highest HCC rates were in Scottish men (peak ASR in 2013 at 15.0 per 100,000 (Table S3). The ASMR for HCC increased from 1.3 to 4.0 per 100,000 and the increase was constant (there were no joinpoints) at 5.8% per year (95% CI 5.4–6.1). In 2017 the highest mortality rates were in Scottish men (10.3 per 100,000), 2.6 times higher than the HCC mortality rates in all persons in the UK (4.0 per 100,000).

**Table 2:** Hepatocellular carcinoma age-standardised incidence and incidence-based mortality rates 1997–2017, by nation, sex, subtype, and year.

Incidence															
	UK			England			Scotland			Wales			Northern Ireland		
	N cases	ASR	95% CI	N cases	ASR	95% CI	N cases	ASR	95% CI	N cases	ASR	95% CI	N cases	ASR	95% CI
**Persons**															
1997	884	1.82	(1.70–1.94)	672	1.66	(1.53–1.79)	145	3.40	(2.87–4.00)	53	2.00	(1.50–2.62)	14	1.19	(0.65–2.00)
2007	1,873	3.54	(3.38–3.71)	1,469	3.33	(3.16–3.50)	243	5.24	(4.6–5.95)	106	3.74	(3.06–4.53)	55	4.21	(3.17–5.49)
2017	3,368	5.45	(5.27–5.64)	2,711	5.25	(5.05–5.45)	409	7.71	(6.98–8.50)	169	5.23	(4.47–6.08)	79	4.95	(3.92–6.17)
**Men**															
1997	647	3.03	(2.80–3.28)	487	2.73	(2.49–2.99)	111	5.99	(4.91–7.24)	40	3.55	(2.53–4.84)	9	1.75	(0.79–3.35)
2007	1,449	6.04	(5.73–6.37)	1,128	5.63	(5.31–5.98)	199	9.71	(8.38–11.19)	80	6.12	(4.84–7.62)	42	7.13	(5.10–9.68)
2017	2,643	9.22	(8.87–9.58)	2,127	8.87	(8.49–9.26)	321	13.31	(11.87–14.87)	131	8.62	(7.20–10.24)	64	8.66	(6.65–11.08)
**Women**															
1997	237	0.87	(0.77–0.99)	185	0.82	(0.70–0.95)	34	1.43	(0.99–2.00)	13	0.87	(0.46–1.50)	5	0.72	(0.23–1.67)
2007	424	1.45	(1.31–1.59)	341	1.39	(1.25–1.55)	44	1.73	(1.25–2.32)	26	1.66	(1.08–2.45)	13	1.78	(0.94–3.05)
2017	725	2.17	(2.02–2.34)	584	2.09	(1.92–2.27)	88	3.08	(2.47–3.80)	38	2.19	(1.54–3.01)	15	1.80	(1.01–2.98)

Incidence-based mortality															
	UK			England			Scotland			Wales			Northern Ireland		
	N cases	ASMR	95% CI	N cases	ASMR	95% CI	N cases	ASMR	95% CI	N cases	ASMR	95% CI	N cases	ASMR	95% CI
**Persons**															
1999	657	1.33	(1.23–1.44)	520	1.26	(1.16–1.38)	82	1.95	(1.55–2.42)	43	1.63	(1.18–2.20)	12	1.01	(0.52–1.77)
2007	1,278	2.44	(2.30–2.57)	987	2.26	(2.12–2.40)	184	3.96	(3.41–4.58)	72	2.56	(2–3.22)	35	2.74	(1.90–3.82)
2017	2,456	3.98	(3.82–4.14)	1,953	3.79	(3.62–3.96)	323	6.12	(5.47–6.83)	131	4.01	(3.35–4.75)	49	3.08	(2.27–4.07)
**Men**															
1999	463	2.11	(1.92–2.32)	372	2.01	(1.81–2.23)	56	3.16	(2.34–4.17)	26	2.24	(1.45–3.29)	9	1.75	(0.77–3.37)
2007	992	4.19	(3.93–4.46)	764	3.87	(3.59–4.15)	149	7.29	(6.14–8.58)	52	4.01	(2.99–5.27)	27	4.72	(3.09–6.90)
2017	1,918	6.74	(6.44–7.05)	1,528	6.41	(6.09–6.74)	244	10.33	(9.05–11.73)	106	6.96	(5.69–8.43)	40	5.55	(3.95–7.57)
**Women**															
1999	194	0.69	(0.60–0.80)	148	0.64	(0.54–0.75)	26	1.04	(0.68–1.52)	17	1.12	(0.65–1.8)	3	0.44	(0.09–1.28)
2007	286	0.99	(0.88–1.11)	223	0.92	(0.80–1.05)	35	1.37	(0.95–1.91)	20	1.31	(0.80–2.03)	8	1.14	(0.49–2.25)
2017	538	1.61	(1.48–1.76)	425	1.53	(1.38–1.68)	79	2.74	(2.17–3.42)	25	1.41	(0.91–2.09)	9	1.05	(0.48–2.00)

ASMR, age-standardised mortality rate; ASR, age-standardised incidence rate.

#### ICCA

ICCA incidence also increased, from 1.8 to 3.3 per 100,000, an AAPC of 3.4%. The most rapid rise was 1997–2009 (APC 4.4%, 95% CI 3.6–5.3) and then this slowed to 2.0% per year (95% CI 0.8–3.2) between 2009 and 2017 (Table 1). When analysed by nation this pattern was seen in England, but in other nations the ICCA rates were more variable and trends less clear (Fig. 1C). Incidence rates were very similar in men and women with ICCA. ASMRs were close to incidence rates (change from 1.4 to 3.0 per 100,000, AAPC 3.8% per year) and did not significantly diverge from incidence over time. Overall, ICCA incidence and mortality rates were similar in England, Scotland and Wales, but slightly lower in NI, although because of sparse data the confidence limits are wide.

#### Other and unspecified liver tumours

There was variation but no clear trend in incidence rates (ASR change 0.76–0.87, AAPC 0.3%, 95% CI -1.9 to 2.6) or mortality rates for other liver tumours (AAPC 0.1%, 95% CI -4.3 to 4.7; Fig. 1D).

Detailed year-by-year estimates of incidence and mortality rates by nation, sex, and subtype are given in Tables S2–S5.

### Survival

**Fig. 2:**
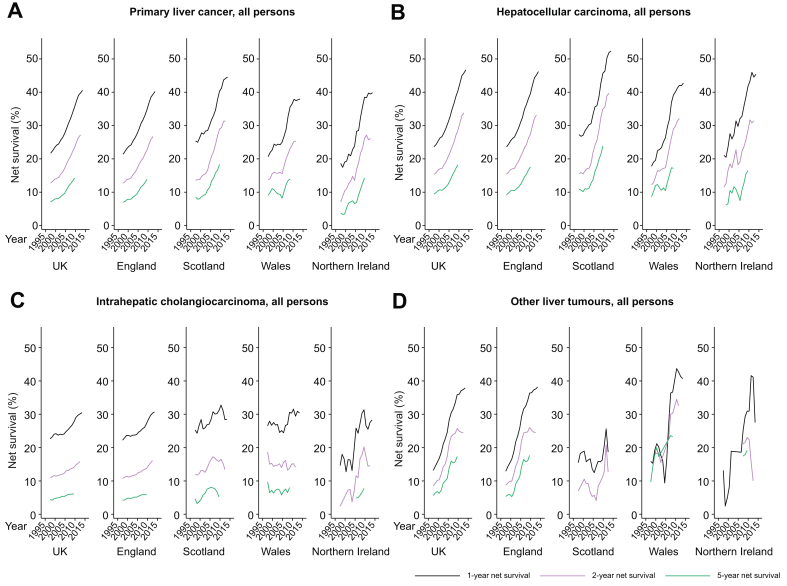
**Age-standardised net-survival in all persons by nation and subtype**. (A) Primary liver cancer; (B) hepatocellular carcinoma; (C) intrahepatic cholangiocarcinoma; (D) other and unspecified liver tumours. Estimates from 5-year rolling cohorts.

For PLC cases 1-, 2-, and 5-year net survival approximately doubled over the study period, reaching 40.6% (2013–2017), 27.2% (2012–2016) and 14.3% (2009–2013), respectively (Table 3 and Fig. 2 and S6). Survival varied by subtype; while 1-year HCC survival nearly doubled from 23.7% in 1997–2001 to 46.7% in 2013–2017, ICCA survival increased by about a third from 22.6% to 30.5% in the same period. One-year survival for other PLCs trebled, from 13.2% to 37.9%. Two- and 5-year survival change followed a similar pattern. Throughout the study period, net PLC survival appeared highest in Scotland, as it had higher HCC 1-, 2-, and 5-year survival than other nations (net 1-year HCC survival in Scotland was 52.4% in 2013–2017 compared with 46.2% in England, 42.0% in Wales, and 45.4% in NI). Although ICCA survival in the most recent cohorts were comparable between countries, NI and then England saw the largest improvements over the study period. Sex did not appear to be strongly associated with survival within subtype (Fig. S4). Complete survival estimates by sex, nation, subtype, and cohort are given in Tables S6–S8.

**Table 3:** Primary liver cancer age-standardised net-survival by nation, sex, subtype, and 5-year cohort.

Cohort	UK			England			Scotland			Wales			Northern Ireland		
	N	Net survival	95% CI	N	Net survival	95% CI	N	Net survival	95% CI	N	Net survival	95% CI	N	Net survival	95% CI
**1-year survival**															
PLC															
1997–2001	11,036	21.7	(20.9–22.5)	9,001	21.4	(20.5–22.3)	1,177	25.4	(22.8–28.0)	632	20.7	(17.5–24)	226	18.6	(13.8–24.1)
2013–2017∗	29,029	40.6	(40.0–41.2)	23,773	40.2	(39.6–40.9)	3,107	44.6	(42.8–46.4)	1,487	37.7	(35.2–40.3)	656	39.9	(36.0–43.7)
Total change		18.9			18.8			19.2			17.0			21.3	
HCC															
1997–2001	4,757	23.7	(22.5–24.9)	3,865	23.6	(22.3–25.0)	564	27.3	(23.6–31.1)	239	17.8	(13.2–23)	89	21.1	(13.2–30.3)
2013–2017∗	16,091	46.7	(45.9–47.5)	12,781	46.2	(45.3–47.1)	2,052	52.4	(50.1–54.6)	808	42.0	(38.5–45.5)	432	45.4	(40.5–50.1)
Total change		23.0			22.6			25.1			24.2			24.3	
ICCA															
1997–2001	4,573	22.6	(21.4–23.9)	3,711	22.2	(20.9–23.6)	488	25.3	(21.4–29.4)	264	26.6	(21.3–32.2)	110	14.6	(8.7–22.1)
2013–2017∗	9,795	30.5	(29.6–31.4)	8,109	30.7	(29.7–31.7)	958	28.5	(25.6–31.5)	568	30.9	(27.1–34.9)	173	28.3	(21.6–35.3)
Total change		7.9			8.5			3.2			4.3			13.7	
Other															
1997–2001	1,648	13.2	(11.6–14.9)	1,397	12.9	(11.2–14.8)	108	15.5	(9.3–23.2)	117	15.9	(9.8–23.2)	26	13.2	(3.6–29.2)
2013–2017∗	3,065	37.9	(36.1–39.6)	2,831	38.2	(36.3–40.0)			Insuff	102	41.3	(31.4–51.1)	50	27.5	(15.8–40.7)
Total change		24.7			25.3				NC		25.4			14.3	
**2-year survival**															
PLC															
1997–2001	2,300	12.8	(12.1–13.4)	1,848	12.7	(12–13.4)	286	13.6	(11.6–15.8)	126	13.7	(11.1–16.6)	41	7.0	(4.1–11.1)
2012–2016∗	10,753	27.2	(26.6–27.7)	8,686	26.7	(26.1–27.3)	1,286	31.4	(29.7–33.2)	530	25.3	(23.0–27.7)	241	26.1	(22.6–29.7)
Total change		14.4			14.0			17.8			11.6			19.1	
HCC															
1997–2001	1,085	15.4	(14.3–16.5)	879	15.5	(14.3–16.7)	148	15.6	(12.6–18.9)	41	12.2	(8.3–16.9)	18	11.6	(5.7–19.8)
2012–2016∗	6,783	33.8	(33.0–34.6)	5,277	33.1	(32.2–34.0)	1,002	39.7	(37.4–42.0)	326	32.1	(28.7–35.5)	185	31.4	(26.8–36)
Total change		18.4			17.6			24.1			19.9			19.8	
ICCA															
1997–2001	993	11.0	(10.1–12.0)	791	10.7	(9.7–11.8)	118	11.9	(9.1–15.1)	67	18.7	(14.0–23.8)	15	2.4	(0.6–7.0)
2012–2016∗	2,739	15.8	(15.0–16.6)	2,273	16.1	(15.3–17)	255	13.4	(11.2–15.8)	157	14.1	(11.3–17.3)	42	14.6	(9.4–20.8)
Total change		4.8			5.4			1.5			-4.6			12.2	
Other															
1997–2001	208	8.5	(7.1–9.9)	172	8.7	(7.2–10.3)	16	7.0	(3.1–13.1)			2 year>1 year			<10
2012–2016∗	1,139	24.4	(22.9–26)	1,066	24.5	(22.9–26.2)	14	12.7	(6.1–21.9)	40	32.6	(23.1–42.5)	18	10.0	(3.3–21.4)
Total change		15.9			15.8			5.7				NC			NC
**5-year survival**															
PLC															
1997–2001	1,300	7.2	(6.7–7.8)	1,053	7	(6.4–7.6)	146	8.5	(6.8–10.5)	81	9.1	(6.8–11.8)	14	3.8	(1.6–7.5)
2009–2013∗	5,018	14.3	(13.8–14.8)	3,975	13.9	(13.3–14.4)	647	18.4	(16.6–20.2)	253	14.0	(11.9–16.3)	123	14.2	(11.0–17.9)
Total change		7.1			6.9			9.9			4.9			10.4	
HCC															
1997–2001	675	9.4	(8.5–10.4)	552	9.3	(8.2–10.3)	80	10.8	(8.1–14.1)	27	8.7	(5.3–13.2)			<10
2009–2013∗	3,172	18.3	(17.5–19.1)	2,386	17.6	(16.7–18.5)	514	23.9	(21.5–26.4)	169	17.2	(14.0–20.7)	88	16.5	(12.2–21.4)
Total change		8.9			8.3			13.1			8.5				NC
ICCA															
1997–2001	464	4.4	(3.8–5.1)	367	4.2	(3.5–5.0)	53	4.6	(2.8–7.2)	46	9.8	(6.3–14.2)			<10
2009–2013∗	1,088	6.1	(5.6–6.7)	890	6.0	(5.4–6.7)	108	5.2	(3.6–7.2)	61	8.0	(5.6–10.9)	21	7.8	(3.6–14.2)
Total change		1.7			1.8			0.6			-1.8				NC
Other															
1997–2001	128	5.6	(4.5–7.0)	111	5.3	(4.2–6.8)			<10	10	9.7	(4.6–17.3)			SE>0.2
2009–2013∗	651	17.3	(15.8–19.0)	607	17.7	(16.0–19.4)			<10	19	23.3	(13.2–35.4)	13	19.2	(10.0–31.0)
Total change		11.7			12.4				NC		13.6				NC

∗1 year earlier for Wales (*i.e.* 2012–2016 for 1-year survival, 2011–2015 for 2-year, and 2008–2012 for 5-year). Suppressed results: Insuff, no deaths or data in at least 1 age band; <10, less than 10 cases in total; SE >0.2, standard error greater than 0.2; 2 year>1 year, 2-year survival longer than 1-year survival. HCC, hepatocellular carcinoma; ICCA, intrahepatic cholangiocarcinoma; NC, not calculatable; PLC, primary liver cancer.

## Discussion

### Main findings

These results from this HCC-UK BASL/NCRAS partnership study of PLC epidemiology in the UK over 20 years show that incidence and incidence-based mortality rates from this largely preventable disease more than doubled, primarily driven by a trebling in HCC rates. The rapid rate of increase in HCC appears to have plateaued since around 2014/15. Men in Scotland had the highest HCC burden, which reached a peak ASR of 15.0 per 100,000 in 2013. ICCA incidence also increased and was the most common PLC in women. PLC survival is improving; it has more than doubled in last 20 years mainly because of the increase in HCC survival, but also an increase in the proportion of PLCs that are HCCs (as prognosis is better in HCC than other PLCs). ICCA survival did not improve as substantially, and the incidence rates remained close to mortality rates. Despite improving PLC survival, mortality increased because of increasing incidence.

### Strengths and limitations

This comprehensive analysis of the epidemiology of liver cancer in the UK over 20 years was conducted on population-level data from high quality cancer registries with near complete population coverage. It is the first study to examine PLC incidence, incidence-based mortality and survival by subtype, sex, and UK nation. Calculation of net-survival allows estimation of disease-specific survival and age-standardisation allows comparison of survival where the background mortality may differ, *e.g.* across time points or regions. The incidence-based mortality method used gives a good estimation of the true mortality rates in a registry with long follow up and high and accurate population-based coverage of all tumours, such as those in the UK.^9^^,^^12^^,^^13^ Patients who do not initially have their cancer registered should be picked up through death certificate only (DCO) registrations. This method permitted more specific analyses by PLC subtype by taking into account the rich diagnostic data including morphology. The main advantage of this method is that it avoids including deaths from other cancers, in particular extrahepatic CCA, that were coded as PLCs on the death certificate.

Some between-nation comparisons were not possible owing to small numbers and lack of statistical power. This was a particular problem for smaller subgroups including the ‘Other’ liver cancer category, and diagnoses and deaths in NI. In addition, cause of death in the data was missing for 5.5% of deaths in NI (compared with approximately 1% in other registries) therefore mortality will be underestimated. A total of 3,730 incidence cases, mainly DCO registrations, were excluded from survival analyses. These cases may have a different risk factor distribution to those that are included. Excluded cases were younger than included cases, and in England slightly more likely to be female. The liver is a common site of metastases and registry staff are trained to identify primary from secondary disease using multiple data sources, however, in some cases this may not have been possible and therefore some cancers included (particularly in the ‘Other’ category), may actually have been metastases incorrectly recorded as liver primaries. This would result in an overestimation of incidence and mortality; however, this would be less than standard mortality estimations that rely on death certificate information only. It is also possible some in the ‘Other’ category are actually HCCs or ICCAs with insufficient information provided to categorise them correctly at registration. Diagnosis is more likely to be based on the death certificate only in this category. Additionally, when English data records were linked to hospital episodes statistics data*,* 25% of other liver tumours were identified as having cirrhosis (compared with 57% of HCCs and 11% % of ICCAs, method detailed in Driver *et al.*^14^). This indicates there are likely to be some HCCs in this category, but this will not account for all unspecified liver tumours.

In the ICD-10 coding system, there is currently no specific code for perihilar CCA (frequently cited as the most common subtype of CCA, followed by distal, and then intrahepatic^15^^,^^16^). There is a specific morphology code in ICD-O-2 (8162), which can be mapped to either C22.1 or C24, but it is rarely used. All 298 ICCA tumours with this code were excluded, however, as 75% of all CCAs in the English registry are coded as intrahepatic it is likely many perihilar CCAs remain included. This makes comparison of ICCA rates in other countries that systematically code perihilar as extrahepatic challenging.^17^ The NI cancer registry is more likely to code CCA as extrahepatic than the English cancer registry (65% of all CCAs in NI *vs.* 25% in England) therefore the lower rates of ICCA, and hence overall PLC, apparent in NI are likely as a result of differences in coding. The WHO Classification of tumours recommended the addition of C24.3 to ICD-O-2 for perihilar CCA, and this has been adopted by the English, Welsh, and NI cancer registries for data from 2018.

### Interpretation and comparison with other studies

The high increase in rates of liver cancer found here are in line with estimates from global studies, which found the annual percentage change in the UK to be amongst the highest in the world.^2^^,^[18], [19], [20] This increase in PLC incidence and mortality, in particular for HCC, mirror the substantial increase in cirrhosis^21^ and liver disease mortality^22^ over this time. The risk factors for liver disease and HCC are well-established, and geographical and temporal variation in their distribution largely explain differences in PLC rates between countries and over time.^2^ An extensive analysis of the leading causes of PLC worldwide found in the UK in 2016 these were, in descending order, hepatitis C (HCV), alcohol consumption, ‘other causes’, and hepatitis B.^2^ This penultimate category, which includes metabolic factors such as obesity, diabetes, and non-alcoholic fatty liver disease (NAFLD) alongside other factors such as aflatoxin B1 and tobacco, was the fastest growing category. Although the relative risk of HCC from obesity and alcohol are lower than for viral hepatitis, these factors are more prevalent in the population and therefore are responsible for a substantial proportion of cases.^23^ The proportion of individuals with obesity or diabetes has increased substantially over this period.^24^ The timing of the plateau in HCC incidence has coincided with the era of direct acting antivirals (DAAs – highly potent and well-tolerated drugs for the treatment of HCV).^25^ The rate of transplants as a result of cirrhosis or HCC caused by HCV in the UK also declined around this time,^26^ as did the rate of HCV-related mortality in England.^27^ Data from the US have shown sustained virologic response induced by DAAs to be associated with an approximately 70% reduced risk of *de novo* HCC in HCV-infected patients.^28^^,^^29^ An Australian study also reported a plateau in the incidence of HCC in patients with HCV in the DAA-era.^30^ However, in the US incidence rates of HCC have plateaued since 2013, and DAAs were not available until late 2014, suggesting other factors may be involved.^31^ Heavy drinking^32^ and HCV infection^33^ are more prevalent in Scotland, particularly in men, and may explain the higher HCC incidence rates seen. The concurrent increase in HCC and ICCA incidence points to some shared risk factors. Cirrhosis substantially increases risk of ICCA (odds ratio 15.3),^34^ therefore the increase in liver disease in the UK may explain some of the rise in ICCA.

EUROCARE-5 explored relative survival following a PLC diagnosis in Europe between 1999 and 2007.^4^ As with our figures for this period, 1-year survival was found to be between 20% and 30% and 5-year survival to be below 10%, which was around the European average. Although some improvements in survival were seen over the EUROCARE-5 study duration, our findings indicate substantially greater improvement in the past 10 years. The improvement in short-, medium-, and longer-term HCC survival suggests a migration to earlier stage at presentation and/or improvements in treatments, both curative and palliative. There have been many advances in treatment options over the past 20 years, including in systemic chemotherapy such as sorafenib, multiple embolization, and ablation techniques, and innovation and increase in the number of liver transplantations, which offers a cure to both the cancer and the underlying liver disease.^35^ During this time clinical decision-making in cancer care has increasingly shifted to multidisciplinary teams,^36^ which has been attributed as a factor improving survival for other cancers.^37^^,^^38^ The proportion of cancers diagnosed via surveillance may have increased over this period. Earlier stage at diagnosis as a result of surveillance could both artefactually increase survival through lead-time bias and genuinely increase it through identification of tumours when both the stage of the cancer and the liver disease are favourable to curative treatment.^39^ Despite improvements, 1-year net survival in the UK remains below 50%. ICCA survival has not improved dramatically and remains poor at 30%; the majority of ICCA cases occur in patients with no known risk factors, early disease is asymptomatic and specific and sensitive markers of early disease do not yet exist,^40^ making surveillance and early diagnosis challenging. There are fewer curative treatment options as liver transplant is not yet recommended for ICCA although trials are ongoing. Additionally, survival following treatments such as resection or chemotherapy is lower than for HCC.

## Conclusions

Liver cancer risk factors are largely modifiable and therefore prevention is possible through lifestyle modifications, vaccination programs, and widespread, effective treatment for HCV. The effect of the latter already appears to be visible. However, as other risk factors such as obesity and diabetes continue to rise and alcohol misuse remains prevalent in the UK, it is unlikely the considerable burden of liver disease and consequently liver cancer will abate. Although substantial improvements in survival have been made, most likely as a result of the multiple innovations in treatments in this field, over half of patients are not alive after 1 year. Reducing incidence through prevention, and further progress in identifying those most at risk, and finding tumours earlier, at a treatable stage, is needed.

## Financial support

This work was supported by the British Association for the Study of the Liver, which received an unrestricted and unconditional award from BTG International Ltd (grant number 52733 1 0). BTG did not have any input into the study design, data collection, analysis or interpretation of the data, writing of the manuscript, review of the manuscript, the decision to submit the manuscript, or the writing of this statement.

## Authors’ contributions

Acquired the data, performed the data analyses and drafted the manuscript: AB

Contributed to the development of the study design, interpretation of the results, and critical revision of the manuscript: all authors

Read and approved the final submitted version of the manuscript: all authors

## Data availability

Data are available from the contributing cancer registries provided there is a justified purpose for the data release, and that there is an appropriate legal basis with safeguards in place to protect the data. Dependent on the request, ethical approval may be required: The National Cancer Registration and Analysis Service, Public Health England; The Welsh Cancer Intelligence and Surveillance Unit, Health Intelligence Division, Public Health Wales; The Scottish Cancer Registry, Information Services Division; The NI Cancer Registry, Queens University, Belfast.

## Conflict of interest

All of the authors completed the ICMJE uniform disclosure form; GA, AM, DT, DH, DW, TGB, and RJD have nothing to disclose. During the conduct of the study, AB reports grants from BTG International Ltd. Outside of the submitted work TJSC reports grants from Sirtex, Bristol-Myers-Squibb, and Bayer and personal fees from Eisai pharmaceuticals, Bayer, AstraZeneca, and Roche. IAR reports personal fees from Roche and Abbvie, outside the submitted work.

Please refer to the accompanying ICMJE disclosure forms for further details.

## Supplementary data

Supplementary information.pdf

CTAT table.pdf
