# Supplementary material for: Primary liver cancer in the UK: Incidence, incidence-based mortality, and survival by subtype, sex, and nation
Source: JHEP Rep. 2021 Jan 19;3(2):100232. doi: 10.1016/j.jhepr.2021.100232 (PMC7966867; doi:10.1016/j.jhepr.2021.100232)
Supplement: Supplementary information.pdf [file mmc1.pdf]

Primary liver cancer in the UK: Incidence, incidence-based mortality, and survival by subtype, sex, and nation

Anya Burton, Daniela Tataru, Robert J. Driver, Thomas G. Bird, Dyfed Huws, David Wallace, Timothy J.S. Cross, Ian A. Rowe, Graeme Alexander, Aileen Marshall

Table of contents

Subtype definitions....................2
Table S1....................3
Table S2....................4
Table S3....................6
Table S4....................8
Table S5....................10
Table S6....................12
Table S7....................16
Table S8....................20
Fig. S1....................24
Fig. S2....................25
Fig. S3....................26
Fig. S4....................27
Fig. S5....................28

1. **Subtype definitions**

All ICD10 C22 (liver) tumours were selected. Subtype was defined primarily based on ICDO-2 morphology, and secondarily on ICD10 code as follows:

Hepatocellular carcinomas

Hepatocellular Carcinoma ICDO morphology codes (8170-8175)

Or, non-specific malignant tumour morphology codes (8000, 8001, 8010) and liver cell carcinoma ICD10 site (C220)

Or, those with Combined Hepatocellular Carcinoma & Cholangiocarcinoma morphology code 8180

Intrahepatic Cholangiocarcinomas

Cholangiocarcinoma ICDO morphology codes (8160 and 8161)

or adenocarcinoma NOS (8140) or non-specific malignant tumour morphology codes (8000, 8001, 8010) and Intrahepatic bile duct carcinoma ICD10 site code (C221)

Other

All other patients with a C22 diagnosis

Exclusions

Those coded as malignant in behaviour but with benign morphology codes

Klatskin (8162)

Table S1 Subtype designation

ICDO Morphology code	C220	C221	C222	C223	C224	C227	C229	Total
**8000**	1,379	1,454	3	8	3	12	3,503	6362
**8001**	18	8	0	0	0	0	13	39
**8002**	0	0	0	0	0	0	3	3
**8003**	0	0	0	0	0	0	3	3
**8004**	0	0	0	0	0	0	3	3
**8010**	477	546	0	3	3	635	2,419	4083
**8011**	0	0	0	0	0	3	0	3
**8012**	3	0	0	0	0	14	3	20
**8020**	0	0	0	0	0	29	3	32
**8021**	0	0	0	0	0	3	3	6
**8022**	0	3	0	0	0	0	3	6
**8031**	0	0	0	0	0	3	3	6
**8032**	3	0	0	0	0	8	3	14
**8033**	0	0	0	0	0	8	0	8
**8041**	3	0	0	0	0	34	15	52
**8046**	0	3	0	0	0	0	0	3
**8050**	0	3	0	0	0	0	0	3
**8060**	0	3	0	0	0	0	0	3
**8070**	3	3	0	0	0	22	14	42
**8071**	3	3	0	0	0	3	0	9
**8140**	79	443	0	0	3	1,626	195	2346
**8141**	0	0	0	0	0	3	0	3
**8160**	210	27,500	0	0	0	8	222	27940
**8161**	0	3	0	0	0	8	0	11
**8170**	38,792	74	3	3	0	8	206	39086
**8171**	178	3	0	0	0	0	3	184
**8172**	3	0	0	0	0	0	0	3
**8173**	3	0	0	0	0	0	0	3
**8174**	3	0	0	0	0	3	0	6
**8175**	3	0	0	0	0	0	0	3
**8180**	275	31	0	0	0	3	8	317
**8190**	3	0	0	0	0	3	3	9
**8211**	0	0	0	0	0	0	3	3
**8240**	8	3	0	0	0	153	44	208
**8241**	0	0	0	0	0	3	0	3
**8246**	8	3	3	0	0	183	52	249
**8260**	0	8	0	0	0	3	0	11
**8263**	0	0	0	0	0	0	3	3
**8310**	3	3	0	0	0	8	3	17
**8430**	0	0	0	0	0	0	3	3
**8440**	3	0	0	0	0	0	3	6
**8453**	0	3	0	0	0	0	0	3
**8470**	0	0	0	0	0	3	0	3
**8480**	3	8	0	0	0	24	3	38
**8481**	3	12	0	0	0	11	3	29

ICDO Morphology code	C220	C221	C222	C223	C224	C227	C229	Row total
**8490**	0	3	0	0	0	3	0	6
**8500**	0	8	0	0	0	8	0	16
**8503**	0	0	0	0	0	3	0	3
**8550**	3	0	0	0	0	0	0	3
**8560**	0	3	0	0	0	8	3	14
**8576**	3	0	0	0	0	0	0	3
**8680**	0	0	0	0	0	3	0	3
**8800**	3	0	0	3	28	0	11	45
**8801**	0	0	0	0	13	0	3	16
**8802**	0	0	0	0	3	0	0	3
**8803**	0	0	0	0	3	0	0	3
**8810**	0	0	0	0	3	0	0	3
**8815**	3	0	0	0	3	0	0	6
**8830**	0	0	0	0	3	0	0	3
**8840**	0	0	0	0	3	0	0	3
**8850**	0	0	0	0	3	0	0	3
**8853**	0	0	0	0	3	0	0	3
**8858**	0	0	0	0	3	0	0	3
**8890**	3	0	0	0	29	0	14	46
**8891**	0	0	0	0	0	0	3	3
**8894**	0	0	0	0	3	0	3	6
**8900**	0	0	0	0	3	0	3	6
**8901**	0	0	0	0	3	0	0	3
**8910**	0	0	0	0	8	0	3	11
**8920**	0	0	0	0	3	0	0	3
**8933**	0	0	0	0	3	0	0	3
**8963**	0	0	0	0	15	0	0	15
**8970**	11	0	378	0	3	0	0	392
**8980**	3	0	0	0	3	0	3	9
**8990**	0	0	0	3	3	0	3	9
**8991**	3	0	0	0	36	0	0	39
**9071**	3	0	0	0	0	0	8	11
**9080**	0	0	0	0	0	3	3	6
**9120**	3	3	0	165	0	0	3	174
**9124**	0	0	0	8	0	0	0	8
**9130**	0	0	0	8	3	0	3	14
**9131**	0	0	0	0	3	0	0	3
**9133**	8	0	0	34	3	3	3	51
**9150**	0	0	0	0	0	3	0	3
**9170**	3	0	0	0	0	0	0	3
**9364**	0	0	0	0	0	0	3	3
**9370**	0	0	0	0	0	0	3	3
**9540**	0	0	0	0	0	0	3	3
**Colum total**	41,515	30,137	387	235	198	2,858	6,823	82153

Summary	
**HCC:**	41,463
**iCCA:**	30,402
**Other**	10156
**Excluded**	3
	**82,024**

Small counts have been suppressed as follows 1 to ≤5 as 3 and 6 to ≤10 as 8, therefore row and column totals are representations only.

Table S2 Primary liver cancer incidence and incidence-based mortality rates, by nation, sex, subtype and year

	INCIDENCE														
	UK			England			Scotland			Wales			Northern Ireland		
Year	Number of Cases	ASR	95% CI	Number of Cases	ASR	95% CI	Number of Cases	ASR	95% CI	Number of Cases	ASR	95% CI	Number of Cases	ASR	95% CI
Persons															
1997	2126	4.41	(4.22-4.6)	1697	4.21	(4.01-4.42)	245	5.83	(5.12-6.61)	126	4.84	(4.03-5.77)	58	5.04	(3.83-6.52)
1998	2178	4.5	(4.31-4.69)	1740	4.29	(4.09-4.5)	261	6.44	(5.68-7.28)	131	5.01	(4.19-5.95)	46	3.79	(2.77-5.07)
1999	2273	4.64	(4.45-4.84)	1832	4.47	(4.27-4.68)	248	5.91	(5.19-6.69)	147	5.6	(4.73-6.59)	46	3.92	(2.86-5.25)
2000	2526	5.14	(4.94-5.34)	2067	5.02	(4.81-5.25)	261	6.14	(5.41-6.93)	136	5.24	(4.39-6.2)	62	5.12	(3.92-6.56)
2001	2487	4.99	(4.8-5.2)	2061	4.95	(4.73-5.17)	258	6.03	(5.32-6.82)	143	5.4	(4.55-6.37)	25	2.04	(1.31-3.03)
2002	2684	5.36	(5.16-5.57)	2214	5.29	(5.07-5.51)	255	5.86	(5.16-6.63)	149	5.61	(4.74-6.6)	66	5.43	(4.18-6.94)
2003	2692	5.33	(5.13-5.54)	2183	5.16	(4.95-5.39)	296	6.9	(6.13-7.74)	160	5.87	(4.99-6.86)	53	4.27	(3.19-5.6)
2004	2864	5.61	(5.41-5.82)	2318	5.44	(5.22-5.66)	327	7.51	(6.71-8.37)	170	6.13	(5.24-7.13)	49	3.88	(2.86-5.14)
2005	3124	6.07	(5.86-6.29)	2565	5.96	(5.73-6.19)	329	7.46	(6.67-8.32)	163	5.94	(5.06-6.93)	67	5.31	(4.11-6.76)
2006	3439	6.63	(6.41-6.86)	2806	6.47	(6.23-6.71)	365	8.19	(7.36-9.08)	185	6.73	(5.79-7.78)	83	6.49	(5.15-8.05)
2007	3670	6.95	(6.72-7.18)	2968	6.73	(6.49-6.98)	403	8.84	(7.99-9.75)	218	7.65	(6.66-8.74)	81	6.04	(4.78-7.52)
2008	3810	7.13	(6.9-7.36)	3117	6.98	(6.73-7.23)	370	7.94	(7.14-8.79)	246	8.7	(7.64-9.87)	77	5.68	(4.48-7.12)
2009	4197	7.75	(7.52-7.99)	3494	7.72	(7.47-7.98)	395	8.42	(7.61-9.3)	223	7.79	(6.8-8.89)	85	6.2	(4.94-7.67)
2010	4442	8.06	(7.82-8.3)	3733	8.11	(7.85-8.38)	417	8.71	(7.89-9.59)	199	6.8	(5.89-7.82)	93	6.43	(5.17-7.89)
2011	4669	8.37	(8.14-8.62)	3803	8.16	(7.9-8.42)	474	9.9	(9.03-10.84)	280	9.49	(8.4-10.67)	112	7.99	(6.57-9.63)
2012	5131	9.05	(8.8-9.3)	4197	8.86	(8.59-9.13)	539	11.06	(10.14-12.03)	287	9.56	(8.48-10.74)	108	7.3	(5.98-8.82)
2013	5759	9.97	(9.72-10.24)	4691	9.72	(9.45-10.01)	622	12.52	(11.55-13.54)	322	10.49	(9.37-11.71)	124	8.49	(7.05-10.1
2014	5839	9.9	(9.65-10.16)	4801	9.73	(9.45-10.01)	623	12.34	(11.39-13.35)	280	9.02	(7.99-10.15)	135	9.01	(7.55-10.6
2015	6006	10.05	(9.8-10.31)	4865	9.74	(9.46-10.02)	649	12.66	(11.7-13.68)	353	11.19	(10.05-12.42	139	9.11	(7.65-10.7
2016	6143	10.11	(9.86-10.37)	5094	10.03	(9.75-10.31)	618	11.85	(10.93-12.82)	296	9.32	(8.29-10.45)	135	8.68	(7.27-10.2
2017	5965	9.64	(9.4-9.89)	4931	9.53	(9.26-9.8)	617	11.71	(10.8-12.68)	288	8.89	(7.89-9.99)	129	8.08	(6.74-9.61)
Men															
1997	1259	6.06	(5.72-6.41)	998	5.74	(5.38-6.12)	157	8.56	(7.24-10.04)	73	6.74	(5.23-8.55)	31	6.38	(4.31-9.09)
1998	1310	6.31	(5.96-6.67)	1047	5.95	(5.58-6.33)	159	10.12	(8.43-12.02)	78	6.96	(5.45-8.75)	26	4.86	(3.11-7.21)
1999	1397	6.69	(6.33-7.06)	1136	6.47	(6.09-6.88)	152	8.64	(7.26-10.19)	89	8.42	(6.68-10.45)	20	3.58	(2.12-5.63)
2000	1556	7.31	(6.94-7.69)	1298	7.22	(6.83-7.64)	151	8.62	(7.24-10.18)	74	7.15	(5.5-9.11)	33	6.15	(4.2-8.68)
2001	1494	7.01	(6.65-7.39)	1240	6.94	(6.54-7.35)	157	8.56	(7.24-10.05)	80	7.33	(5.71-9.25)	17	3.63	(1.92-6.09)
2002	1668	7.77	(7.39-8.17)	1373	7.64	(7.23-8.07)	165	8.73	(7.42-10.21)	89	8.21	(6.49-10.22)	41	8.08	(5.64-11.1
2003	1696	7.76	(7.38-8.15)	1374	7.48	(7.08-7.9)	186	10.39	(8.89-12.06)	102	8.67	(7.02-10.59)	34	6.27	(4.3-8.83)
2004	1762	7.89	(7.52-8.28)	1428	7.64	(7.24-8.06)	205	11.18	(9.65-12.88)	98	7.81	(6.32-9.53)	31	5.73	(3.84-8.21)
2005	1956	8.63	(8.24-9.03)	1613	8.49	(8.07-8.92)	210	10.88	(9.41-12.51)	89	7.5	(5.98-9.28)	44	7.97	(5.75-10.7
2006	2184	9.64	(9.23-10.06)	1777	9.33	(8.89-9.78)	241	12.78	(11.16-14.57)	113	9.58	(7.83-11.58)	53	9.56	(7.06-12.6
2007	2320	9.9	(9.49-10.32)	1860	9.49	(9.05-9.94)	276	14.08	(12.4-15.92)	131	10.33	(8.61-12.29)	53	8.88	(6.59-11.6
2008	2441	10.27	(9.86-10.7)	1996	10.06	(9.61-10.52)	246	12.01	(10.53-13.65)	149	11.66	(9.83-13.73)	50	7.98	(5.9-10.56)
2009	2657	11.01	(10.58-11.44)	2172	10.75	(10.3-11.23)	278	13.39	(11.84-15.1)	146	11.45	(9.62-13.51)	61	10.38	(7.84-13.4
2010	2811	11.37	(10.95-11.81)	2346	11.36	(10.9-11.84)	287	13.51	(11.96-15.21)	119	8.92	(7.37-10.7)	59	9.29	(6.98-12.1)
2011	2978	11.88	(11.45-12.32)	2402	11.43	(10.97-11.9)	323	15.23	(13.56-17.03)	179	13.5	(11.55-15.6	74	12.11	(9.44-15.2
2012	3322	12.92	(12.48-13.38)	2684	12.48	(12-12.96)	360	16.44	(14.75-18.25)	191	14.24	(12.26-16.4	87	12.88	(10.28-15.
2013	3744	14.39	(13.93-14.87)	3017	13.85	(13.36-14.36)	440	20.09	(18.21-22.11)	205	14.33	(12.42-16.4	82	13.35	(10.5-16.7
2014	3841	14.36	(13.91-14.83)	3134	13.99	(13.5-14.5)	425	18.96	(17.16-20.89)	196	13.7	(11.83-15.7	86	12.86	(10.22-15.
2015	3879	14.19	(13.75-14.65)	3116	13.63	(13.15-14.12)	437	19.05	(17.27-20.97)	244	16.62	(14.57-18.8	82	11.76	(9.33-14.6
2016	4055	14.6	(14.15-15.06)	3342	14.37	(13.88-14.87)	421	18.03	(16.32-19.87)	208	14.18	(12.3-16.26)	84	11.61	(9.22-14.4
2017	3928	13.85	(13.42-14.29)	3232	13.61	(13.14-14.09)	423	17.81	(16.13-19.62)	184	12.21	(10.5-14.13)	89	11.98	(9.6-14.76)
Women															
1997	867	3.1	(2.9-3.31)	699	2.99	(2.77-3.22)	88	3.61	(2.9-4.46)	53	3.58	(2.68-4.69)	27	3.84	(2.53-5.6)
1998	868	3.09	(2.89-3.31)	693	2.96	(2.74-3.19)	102	4.16	(3.39-5.06)	53	3.52	(2.63-4.62)	20	3.03	(1.85-4.67)
1999	876	3.12	(2.92-3.34)	696	2.97	(2.75-3.2)	96	3.95	(3.19-4.82)	58	3.88	(2.94-5.02)	26	3.88	(2.53-5.69)
2000	970	3.42	(3.21-3.65)	769	3.25	(3.02-3.49)	110	4.43	(3.64-5.35)	62	3.99	(3.05-5.13)	29	4.27	(2.85-6.13)
2001	993	3.48	(3.27-3.71)	821	3.44	(3.21-3.68)	101	4.12	(3.35-5)	63	4.16	(3.18-5.33)	8	1.16	(0.5-2.29)
2002	1016	3.52	(3.3-3.74)	841	3.48	(3.25-3.72)	90	3.55	(2.85-4.36)	60	3.99	(3.04-5.15)	25	3.61	(2.32-5.34)
2003	996	3.44	(3.23-3.66)	809	3.33	(3.1-3.57)	110	4.45	(3.66-5.37)	58	3.77	(2.86-4.89)	19	2.64	(1.58-4.14)
2004	1102	3.79	(3.57-4.02)	890	3.66	(3.42-3.91)	122	4.82	(4-5.77)	72	4.64	(3.62-5.85)	18	2.54	(1.5-4.03)
2005	1168	4	(3.77-4.24)	952	3.89	(3.65-4.15)	119	4.69	(3.88-5.62)	74	4.84	(3.79-6.08)	23	3.24	(2.04-4.87)
2006	1255	4.29	(4.06-4.54)	1029	4.22	(3.96-4.49)	124	4.82	(4.01-5.75)	72	4.75	(3.71-5.99)	30	4.04	(2.72-5.78)
2007	1350	4.54	(4.3-4.79)	1108	4.47	(4.2-4.74)	127	4.94	(4.11-5.88)	87	5.35	(4.27-6.61)	28	3.73	(2.47-5.4)
2008	1369	4.55	(4.31-4.8)	1121	4.45	(4.19-4.72)	124	4.74	(3.94-5.66)	97	6.03	(4.88-7.37)	27	3.6	(2.36-5.25)
2009	1540	5.07	(4.82-5.33)	1322	5.22	(4.94-5.51)	117	4.44	(3.67-5.33)	77	4.7	(3.7-5.89)	24	3.08	(1.97-4.59)
2010	1631	5.3	(5.04-5.57)	1387	5.4	(5.12-5.7)	130	4.86	(4.06-5.78)	80	4.86	(3.84-6.06)	34	4.26	(2.94-5.97)
2011	1691	5.44	(5.19-5.71)	1401	5.41	(5.13-5.7)	151	5.6	(4.74-6.57)	101	6.22	(5.06-7.57)	38	4.68	(3.3-6.44)
2012	1809	5.76	(5.49-6.03)	1513	5.78	(5.49-6.09)	179	6.52	(5.59-7.55)	96	5.76	(4.66-7.04)	21	2.44	(1.5-3.74)
2013	2015	6.3	(6.03-6.59)	1674	6.29	(5.99-6.6)	182	6.54	(5.62-7.56)	117	6.81	(5.62-8.18)	42	5.08	(3.66-6.89)
2014	1998	6.11	(5.85-6.39)	1667	6.08	(5.79-6.38)	198	7.13	(6.17-8.2)	84	5.01	(3.99-6.21)	49	5.88	(4.35-7.79)
2015	2127	6.45	(6.18-6.74)	1749	6.35	(6.05-6.66)	212	7.46	(6.49-8.54)	109	6.3	(5.17-7.62)	57	6.77	(5.12-8.78)
2016	2088	6.28	(6.01-6.56)	1752	6.3	(6-6.6)	197	6.89	(5.96-7.93)	88	5.08	(4.07-6.27)	51	6.01	(4.47-7.92)
2017	2037	6.03	(5.77-6.3)	1699	6.02	(5.74-6.32)	194	6.69	(5.78-7.71)	104	5.86	(4.78-7.11)	40	4.58	(3.26-6.24)

INCIDENCE-BASED MORTALITY

Year	UK Number of Cases	UK ASMR	UK 95% CI	England Number of Cases	England ASMR	England 95% CI	Scotland Number of Cases	Scotland ASMR	Scotland 95% CI	Wales Number of Cases	Wales ASMR	Wales 95% CI	Northern Ireland Number of Cases	Northern Ireland ASMR	Northern Ireland 95% CI
Persons															
1999	1594	3.28	(3.12-3.44)	1275	3.13	(2.96-3.31)	187	4.55	(3.91-5.25)	98	3.76	(3.05-4.59)	34	2.9	(2-4.07)
2000	1738	3.55	(3.38-3.72)	1423	3.47	(3.29-3.66)	183	4.31	(3.71-4.99)	96	3.78	(3.05-4.62)	36	3.03	(2.12-4.2)
2001	1827	3.69	(3.53-3.87)	1522	3.68	(3.5-3.87)	178	4.16	(3.57-4.82)	107	4.05	(3.32-4.9)	20	1.6	(0.97-2.47)
2002	1958	3.92	(3.75-4.1)	1617	3.87	(3.69-4.07)	191	4.39	(3.78-5.06)	106	3.96	(3.24-4.8)	44	3.68	(2.65-4.96)
2003	2061	4.1	(3.92-4.28)	1701	4.04	(3.85-4.24)	209	4.84	(4.2-5.54)	116	4.3	(3.55-5.16)	35	2.93	(2.03-4.08)
2004	2169	4.26	(4.08-4.44)	1752	4.11	(3.92-4.31)	256	5.9	(5.19-6.67)	131	4.77	(3.98-5.66)	30	2.32	(1.56-3.32)
2005	2315	4.52	(4.34-4.71)	1872	4.37	(4.17-4.57)	253	5.75	(5.06-6.5)	141	5.12	(4.31-6.04)	49	3.94	(2.91-5.22)
2006	2495	4.83	(4.64-5.02)	2035	4.71	(4.5-4.92)	280	6.32	(5.6-7.11)	139	5.05	(4.24-5.96)	41	3.06	(2.19-4.17)
2007	2669	5.1	(4.91-5.3)	2135	4.89	(4.69-5.11)	307	6.74	(6-7.54)	165	5.87	(5.01-6.85)	62	4.81	(3.68-6.18)
2008	2792	5.22	(5.03-5.42)	2306	5.15	(4.94-5.37)	268	5.78	(5.11-6.52)	173	6.07	(5.19-7.05)	45	3.33	(2.42-4.46)
2009	3009	5.58	(5.38-5.78)	2505	5.55	(5.34-5.78)	271	5.79	(5.12-6.53)	176	6.2	(5.31-7.19)	57	4.17	(3.15-5.41)
2010	3101	5.66	(5.46-5.87)	2622	5.73	(5.52-5.96)	281	5.93	(5.25-6.66)	136	4.63	(3.88-5.48)	62	4.48	(3.42-5.75)
2011	3323	5.99	(5.78-6.19)	2766	5.96	(5.74-6.19)	326	6.85	(6.12-7.63)	164	5.59	(4.77-6.52)	67	4.77	(3.69-6.07)
2012	3646	6.45	(6.24-6.66)	3050	6.45	(6.22-6.68)	344	7.15	(6.41-7.95)	183	6.13	(5.27-7.08)	69	4.71	(3.66-5.97)
2013	3896	6.77	(6.56-6.99)	3140	6.53	(6.3-6.76)	439	8.9	(8.08-9.78)	225	7.34	(6.41-8.37)	92	6.35	(5.11-7.79)
2014	4059	6.9	(6.69-7.12)	3314	6.74	(6.51-6.97)	421	8.41	(7.62-9.25)	239	7.64	(6.7-8.68)	85	5.7	(4.55-7.06)
2015	4093	6.85	(6.65-7.07)	3303	6.62	(6.39-6.85)	446	8.74	(7.95-9.6)	242	7.69	(6.75-8.73)	102	6.66	(5.43-8.09)
2016	4307	7.11	(6.9-7.33)	3528	6.96	(6.73-7.19)	465	9.02	(8.22-9.89)	203	6.42	(5.56-7.37)	111	7.2	(5.92-8.68)
2017	4651	7.53	(7.32-7.75)	3818	7.39	(7.16-7.63)	513	9.79	(8.95-10.67)	237	7.28	(6.38-8.27)	83	5.24	(4.17-6.51)
Men															
1999	962	4.69	(4.38-5.01)	770	4.43	(4.11-4.77)	116	7.22	(5.84-8.8)	60	5.79	(4.34-7.55)	16	2.98	(1.66-4.91)
2000	1057	5	(4.69-5.32)	883	4.95	(4.62-5.3)	104	5.71	(4.64-6.94)	51	5.23	(3.78-7.02)	19	3.76	(2.23-5.92)
2001	1072	5.12	(4.81-5.45)	908	5.18	(4.83-5.54)	98	5.4	(4.36-6.61)	51	4.74	(3.46-6.32)	15	2.65	(1.47-4.4)
2002	1169	5.45	(5.14-5.79)	956	5.32	(4.98-5.68)	122	6.51	(5.39-7.8)	64	5.8	(4.41-7.47)	27	5.7	(3.61-8.48)
2003	1303	5.98	(5.65-6.32)	1072	5.86	(5.51-6.24)	139	7.47	(6.25-8.85)	70	6.21	(4.76-7.95)	22	4.45	(2.62-6.97)
2004	1317	5.95	(5.62-6.28)	1069	5.75	(5.41-6.12)	152	8.6	(7.23-10.14)	77	6.17	(4.86-7.73)	19	3.22	(1.93-5.05)
2005	1429	6.38	(6.04-6.72)	1158	6.17	(5.81-6.55)	162	8.49	(7.19-9.94)	76	6.27	(4.91-7.88)	33	6.33	(4.32-8.94)
2006	1581	7	(6.65-7.36)	1294	6.83	(6.45-7.22)	178	9.31	(7.96-10.82)	82	7.03	(5.53-8.8)	27	4.42	(2.88-6.47)
2007	1662	7.22	(6.87-7.59)	1315	6.83	(6.46-7.22)	218	11.25	(9.73-12.93)	94	7.59	(6.1-9.33)	35	6.2	(4.29-8.66)
2008	1749	7.4	(7.05-7.77)	1437	7.26	(6.88-7.66)	172	8.5	(7.25-9.9)	108	8.66	(7.06-10.49)	32	5.24	(3.56-7.42)
2009	1850	7.75	(7.39-8.12)	1528	7.64	(7.25-8.04)	182	8.96	(7.66-10.4)	104	8.36	(6.79-10.19)	36	5.82	(4.04-8.1)
2010	1936	7.95	(7.59-8.32)	1614	7.95	(7.56-8.35)	201	9.61	(8.31-11.07)	80	5.88	(4.65-7.32)	41	6.78	(4.79-9.3)
2011	2077	8.39	(8.03-8.77)	1702	8.21	(7.82-8.61)	219	10.51	(9.12-12.06)	105	7.92	(6.45-9.62)	51	8.42	(6.21-11.1
2012	2279	8.92	(8.56-9.3)	1905	8.91	(8.51-9.32)	211	9.83	(8.52-11.27)	116	8.58	(7.07-10.31)	47	7.11	(5.2-9.49)
2013	2464	9.53	(9.15-9.91)	1958	9.03	(8.63-9.45)	303	13.98	(12.41-15.69)	139	9.91	(8.31-11.73)	64	10.34	(7.85-13.3
2014	2569	9.69	(9.31-10.07)	2060	9.27	(8.87-9.69)	281	12.74	(11.26-14.36)	171	11.95	(10.21-13.9)	57	8.62	(6.47-11.2
2015	2576	9.53	(9.17-9.91)	2070	9.16	(8.76-9.57)	291	12.82	(11.36-14.41)	148	10.28	(8.67-12.1)	67	9.8	(7.55-12.4
2016	2757	10	(9.62-10.38)	2244	9.71	(9.31-10.12)	311	13.63	(12.12-15.28)	138	9.35	(7.84-11.06)	64	9.07	(6.95-11.6
2017	2981	10.57	(10.19-10.96)	2435	10.3	(9.89-10.72)	323	13.87	(12.37-15.49)	168	11.17	(9.53-13.01)	55	7.6	(5.71-9.92)
Women															
1999	632	2.23	(2.06-2.41)	505	2.14	(1.95-2.33)	71	2.87	(2.24-3.62)	38	2.5	(1.76-3.44)	18	2.69	(1.59-4.26)
2000	681	2.4	(2.22-2.59)	540	2.28	(2.09-2.48)	79	3.22	(2.55-4.01)	45	2.92	(2.12-3.91)	17	2.49	(1.45-3.98)
2001	755	2.65	(2.46-2.85)	614	2.57	(2.37-2.78)	80	3.25	(2.57-4.05)	56	3.67	(2.76-4.78)	5	0.75	(0.24-1.74)
2002	789	2.74	(2.55-2.94)	661	2.74	(2.54-2.96)	69	2.76	(2.15-3.5)	42	2.73	(1.96-3.7)	17	2.44	(1.42-3.92)
2003	758	2.62	(2.43-2.81)	629	2.59	(2.39-2.81)	70	2.81	(2.19-3.56)	46	3	(2.19-4.01)	13	1.95	(1.04-3.34)
2004	852	2.92	(2.73-3.13)	683	2.8	(2.59-3.02)	104	4.11	(3.35-4.98)	54	3.55	(2.66-4.63)	11	1.54	(0.76-2.77)
2005	886	3.02	(2.82-3.23)	714	2.91	(2.7-3.13)	91	3.59	(2.89-4.41)	65	4.2	(3.23-5.36)	16	2.23	(1.27-3.63)
2006	914	3.11	(2.91-3.32)	741	3.01	(2.8-3.24)	102	4	(3.26-4.86)	57	3.7	(2.8-4.81)	14	1.83	(0.99-3.08)
2007	1007	3.41	(3.2-3.63)	820	3.33	(3.1-3.57)	89	3.43	(2.75-4.22)	71	4.48	(3.48-5.66)	27	3.66	(2.41-5.34)
2008	1043	3.43	(3.23-3.65)	869	3.42	(3.2-3.66)	96	3.65	(2.95-4.46)	65	3.99	(3.07-5.1)	13	1.67	(0.88-2.86)
2009	1159	3.8	(3.58-4.03)	977	3.84	(3.6-4.09)	89	3.4	(2.73-4.18)	72	4.43	(3.45-5.58)	21	2.71	(1.67-4.15)
2010	1165	3.77	(3.55-3.99)	1008	3.91	(3.67-4.16)	80	3.01	(2.38-3.75)	56	3.36	(2.53-4.37)	21	2.7	(1.66-4.14)
2011	1246	4	(3.78-4.23)	1064	4.1	(3.85-4.35)	107	3.91	(3.2-4.73)	59	3.58	(2.72-4.63)	16	1.98	(1.12-3.22)
2012	1367	4.34	(4.11-4.58)	1145	4.35	(4.1-4.62)	133	4.89	(4.09-5.8)	67	4.03	(3.11-5.12)	22	2.59	(1.62-3.92)
2013	1432	4.45	(4.22-4.69)	1182	4.42	(4.17-4.68)	136	4.91	(4.12-5.82)	86	4.91	(3.92-6.08)	28	3.32	(2.2-4.81)
2014	1490	4.56	(4.33-4.8)	1254	4.59	(4.34-4.86)	140	4.96	(4.17-5.86)	68	4.04	(3.13-5.14)	28	3.35	(2.22-4.85)
2015	1517	4.57	(4.35-4.81)	1233	4.45	(4.2-4.7)	155	5.44	(4.61-6.37)	94	5.43	(4.38-6.65)	35	4.12	(2.87-5.74)
2016	1550	4.65	(4.42-4.89)	1284	4.59	(4.34-4.85)	154	5.44	(4.61-6.37)	65	3.73	(2.87-4.76)	47	5.49	(4.02-7.3)
2017	1670	4.93	(4.7-5.18)	1383	4.89	(4.63-5.15)	190	6.53	(5.63-7.53)	69	3.86	(3-4.89)	28	3.23	(2.14-4.68)

ASR, Age-standardised Rate; ASMR, Age-standardised Mortality Rate; CI, Confidence Interval

Table S3 Hepatocellular carcinoma incidence and incidence-based mortality rates, by nation, sex, subtype and year

	INCIDENCE														
	UK			England			Scotland			Wales			Northern Ireland		
Year	Number of Cases	ASR	95% CI	Number of Cases	ASR	95% CI	Number of Cases	ASR	95% CI	Number of Cases	ASR	95% CI	Number of Cases	ASR	95% CI
Persons															
1997	884	1.82	(1.7-1.94)	672	1.66	(1.53-1.79)	145	3.40	(2.87-4)	53	2.00	(1.5-2.62)	14	1.19	(0.65-2)
1998	900	1.84	(1.72-1.97)	720	1.76	(1.63-1.9)	114	2.71	(2.24-3.26)	51	1.98	(1.47-2.61)	15	1.22	(0.68-2.02)
1999	1000	2.02	(1.9-2.15)	806	1.95	(1.81-2.09)	114	2.67	(2.2-3.21)	60	2.27	(1.73-2.92)	20	1.63	(0.99-2.53)
2000	1077	2.17	(2.04-2.3)	889	2.14	(2-2.28)	124	2.9	(2.41-3.46)	45	1.73	(1.26-2.31)	19	1.51	(0.91-2.37)
2001	1147	2.3	(2.16-2.43)	934	2.24	(2.1-2.39)	135	3.12	(2.61-3.69)	57	2.13	(1.61-2.77)	21	1.75	(1.07-2.69)
2002	1278	2.55	(2.41-2.69)	1029	2.45	(2.31-2.61)	150	3.42	(2.89-4.01)	64	2.39	(1.84-3.05)	35	2.82	(1.95-3.95)
2003	1270	2.5	(2.36-2.64)	1014	2.38	(2.24-2.53)	167	3.83	(3.27-4.46)	63	2.34	(1.8-3)	26	2.15	(1.4-3.15)
2004	1332	2.6	(2.46-2.75)	1051	2.46	(2.31-2.61)	183	4.1	(3.53-4.75)	76	2.75	(2.16-3.45)	22	1.69	(1.06-2.57)
2005	1504	2.91	(2.76-3.06)	1227	2.83	(2.68-3)	175	3.91	(3.35-4.54)	64	2.35	(1.81-3.01)	38	2.9	(2.04-3.99)
2006	1657	3.18	(3.03-3.34)	1318	3.03	(2.86-3.19)	219	4.87	(4.25-5.57)	86	3.1	(2.48-3.84)	34	2.67	(1.84-3.74)
2007	1873	3.54	(3.38-3.71)	1469	3.33	(3.16-3.5)	243	5.24	(4.6-5.95)	106	3.74	(3.06-4.53)	55	4.21	(3.17-5.49)
2008	1800	3.36	(3.2-3.52)	1398	3.12	(2.96-3.29)	235	5	(4.38-5.68)	119	4.21	(3.49-5.04)	48	3.59	(2.64-4.77)
2009	2041	3.77	(3.6-3.93)	1619	3.58	(3.4-3.75)	255	5.4	(4.76-6.11)	114	4.02	(3.31-4.83)	53	3.9	(2.91-5.11)
2010	2258	4.09	(3.92-4.26)	1829	3.97	(3.79-4.16)	270	5.59	(4.94-6.3)	104	3.54	(2.89-4.29)	55	3.83	(2.88-5)
2011	2422	4.35	(4.18-4.53)	1887	4.06	(3.87-4.24)	315	6.51	(5.81-7.28)	159	5.38	(4.57-6.29)	61	4.36	(3.33-5.61)
2012	2653	4.68	(4.5-4.86)	2066	4.36	(4.18-4.56)	356	7.21	(6.48-8.01)	154	5.12	(4.34-6)	77	5.28	(4.16-6.61)
2013	3043	5.28	(5.09-5.47)	2366	4.91	(4.71-5.11)	412	8.25	(7.47-9.09)	176	5.75	(4.93-6.67)	89	6.04	(4.84-7.44)
2014	3160	5.36	(5.18-5.55)	2525	5.13	(4.93-5.33)	402	7.91	(7.15-8.72)	142	4.57	(3.84-5.39)	91	6.06	(4.88-7.45)
2015	3291	5.52	(5.33-5.71)	2599	5.22	(5.02-5.42)	416	8.11	(7.34-8.93)	187	5.9	(5.08-6.81)	89	5.79	(4.65-7.14)
2016	3496	5.76	(5.57-5.95)	2813	5.55	(5.35-5.76)	430	8.18	(7.43-9)	167	5.24	(4.47-6.1)	86	5.48	(4.38-6.77)
2017	3368	5.45	(5.27-5.64)	2711	5.25	(5.05-5.45)	409	7.71	(6.98-8.5)	169	5.23	(4.47-6.08)	79	4.95	(3.92-6.17)
Men															
1997	647	3.03	(2.8-3.28)	487	2.73	(2.49-2.99)	111	5.99	(4.91-7.24)	40	3.55	(2.53-4.84)	9	1.75	(0.79-3.35)
1998	660	3.06	(2.82-3.3)	532	2.92	(2.68-3.19)	84	4.7	(3.73-5.83)	35	3.19	(2.16-4.51)	9	1.54	(0.68-2.96)
1999	724	3.29	(3.05-3.54)	587	3.18	(2.92-3.45)	81	4.34	(3.4-5.45)	41	3.54	(2.53-4.81)	15	2.7	(1.48-4.52)
2000	837	3.81	(3.55-4.08)	700	3.77	(3.49-4.06)	89	5.01	(3.99-6.21)	35	3.19	(2.17-4.49)	13	2.37	(1.23-4.09)
2001	842	3.8	(3.54-4.07)	684	3.68	(3.4-3.97)	107	5.76	(4.69-6.98)	36	2.98	(2.08-4.14)	15	3.37	(1.7-5.82)
2002	980	4.46	(4.18-4.75)	795	4.33	(4.03-4.65)	116	6	(4.95-7.22)	45	3.81	(2.74-5.14)	24	4.67	(2.83-7.18)
2003	969	4.3	(4.02-4.58)	779	4.09	(3.8-4.4)	122	6.55	(5.41-7.86)	50	4.34	(3.18-5.76)	18	3.56	(2.07-5.68)
2004	1012	4.43	(4.16-4.72)	802	4.2	(3.91-4.51)	138	7.09	(5.94-8.4)	55	4.43	(3.33-5.78)	17	2.95	(1.69-4.78)
2005	1158	5.01	(4.72-5.31)	951	4.91	(4.6-5.24)	135	6.84	(5.7-8.13)	44	3.6	(2.61-4.84)	28	4.93	(3.25-7.18)
2006	1300	5.56	(5.26-5.88)	1043	5.3	(4.98-5.64)	167	8.66	(7.36-10.12)	63	5.1	(3.9-6.55)	27	4.62	(3.02-6.76)
2007	1449	6.04	(5.73-6.37)	1128	5.63	(5.31-5.98)	199	9.71	(8.38-11.19)	80	6.12	(4.84-7.62)	42	7.13	(5.1-9.68)
2008	1413	5.8	(5.49-6.11)	1111	5.45	(5.13-5.78)	179	8.57	(7.35-9.94)	88	6.76	(5.41-8.33)	35	5.73	(3.97-7.99)
2009	1595	6.47	(6.15-6.8)	1253	6.07	(5.73-6.42)	205	9.72	(8.41-11.17)	93	7.2	(5.78-8.86)	44	7.54	(5.39-10.24)
2010	1753	6.96	(6.63-7.29)	1422	6.76	(6.41-7.12)	215	9.97	(8.66-11.43)	77	5.65	(4.45-7.09)	39	6.09	(4.29-8.37)
2011	1851	7.23	(6.9-7.57)	1430	6.66	(6.32-7.02)	249	11.54	(10.11-13.11)	124	9.11	(7.55-10.9)	48	7.69	(5.65-10.23)
2012	2061	7.91	(7.56-8.26)	1596	7.31	(6.95-7.68)	281	12.68	(11.22-14.29)	121	8.97	(7.42-10.75)	63	9.33	(7.14-11.97)
2013	2365	8.93	(8.57-9.3)	1825	8.24	(7.86-8.63)	335	14.98	(13.39-16.71)	140	9.74	(8.18-11.51)	65	10.02	(7.68-12.83)
2014	2491	9.17	(8.81-9.54)	1994	8.78	(8.39-9.17)	317	13.8	(12.3-15.43)	113	7.69	(6.33-9.25)	67	9.95	(7.65-12.71)
2015	2551	9.22	(8.86-9.59)	2021	8.74	(8.36-9.13)	325	13.95	(12.45-15.58)	150	10.05	(8.5-11.81)	55	7.74	(5.81-10.1)
2016	2716	9.68	(9.31-10.05	2184	9.31	(8.92-9.71)	330	13.82	(12.35-15.42)	137	9.3	(7.8-11.01)	65	8.8	(6.78-11.24)
2017	2643	9.22	(8.87-9.58)	2127	8.87	(8.49-9.26)	321	13.31	(11.87-14.87)	131	8.62	(7.2-10.24)	64	8.66	(6.65-11.08)
Women															
1997	237	0.87	(0.77-0.99)	185	0.82	(0.7-0.95)	34	1.43	(0.99-2)	13	0.87	(0.46-1.5)	5	0.72	(0.23-1.67)
1998	240	0.86	(0.75-0.98)	188	0.81	(0.7-0.93)	30	1.22	(0.82-1.74)	16	1.11	(0.63-1.81)	6	0.9	(0.33-1.96)
1999	276	0.99	(0.88-1.12)	219	0.95	(0.83-1.09)	33	1.35	(0.93-1.9)	19	1.24	(0.74-1.94)	5	0.74	(0.24-1.72)
2000	240	0.86	(0.75-0.97)	189	0.81	(0.7-0.93)	35	1.42	(0.99-1.98)	10	0.66	(0.32-1.22)	6	0.9	(0.33-1.96)
2001	305	1.08	(0.96-1.21)	250	1.06	(0.93-1.2)	28	1.14	(0.76-1.65)	21	1.42	(0.87-2.18)	6	0.86	(0.31-1.88)
2002	298	1.05	(0.94-1.18)	234	0.99	(0.87-1.13)	34	1.36	(0.94-1.91)	19	1.29	(0.77-2.01)	11	1.6	(0.8-2.87)
2003	301	1.06	(0.94-1.18)	235	0.99	(0.86-1.12)	45	1.81	(1.32-2.42)	13	0.84	(0.45-1.45)	8	1.12	(0.48-2.21)
2004	320	1.12	(1-1.25)	249	1.04	(0.91-1.18)	45	1.81	(1.32-2.42)	21	1.36	(0.84-2.09)	5	0.73	(0.24-1.69)
2005	346	1.21	(1.09-1.35)	276	1.15	(1.02-1.3)	40	1.61	(1.15-2.2)	20	1.37	(0.83-2.12)	10	1.43	(0.68-2.63)
2006	357	1.25	(1.12-1.38)	275	1.15	(1.02-1.3)	52	2.03	(1.52-2.67)	23	1.56	(0.98-2.34)	7	0.98	(0.39-2.02)
2007	424	1.45	(1.31-1.59)	341	1.39	(1.25-1.55)	44	1.73	(1.25-2.32)	26	1.66	(1.08-2.45)	13	1.78	(0.94-3.05)
2008	387	1.31	(1.18-1.45)	287	1.16	(1.03-1.31)	56	2.16	(1.63-2.81)	31	1.97	(1.33-2.8)	13	1.74	(0.92-2.98)
2009	446	1.51	(1.37-1.65)	366	1.47	(1.33-1.64)	50	1.93	(1.43-2.55)	21	1.41	(0.87-2.16)	9	1.17	(0.53-2.22)
2010	505	1.68	(1.54-1.83)	407	1.63	(1.47-1.8)	55	2.07	(1.56-2.7)	27	1.64	(1.08-2.39)	16	2.06	(1.17-3.36)
2011	571	1.89	(1.73-2.05)	457	1.81	(1.65-1.99)	66	2.46	(1.9-3.13)	35	2.21	(1.54-3.09)	13	1.63	(0.86-2.8)
2012	592	1.92	(1.77-2.08)	470	1.84	(1.67-2.01)	75	2.76	(2.17-3.46)	33	1.99	(1.37-2.81)	14	1.69	(0.92-2.85)
2013	678	2.18	(2.01-2.35)	541	2.08	(1.91-2.26)	77	2.82	(2.23-3.53)	36	2.18	(1.52-3.03)	24	3	(1.91-4.47)
2014	669	2.09	(1.94-2.26)	531	1.98	(1.81-2.15)	85	3.11	(2.49-3.85)	29	1.77	(1.18-2.54)	24	2.92	(1.87-4.36)
2015	740	2.31	(2.15-2.48)	578	2.16	(1.99-2.35)	91	3.26	(2.62-4)	37	2.22	(1.56-3.06)	34	4.06	(2.8-5.68)
2016	780	2.39	(2.22-2.56)	629	2.31	(2.13-2.5)	100	3.5	(2.85-4.26)	30	1.7	(1.15-2.44)	21	2.5	(1.54-3.83)
2017	725	2.17	(2.02-2.34)	584	2.09	(1.92-2.27)	88	3.08	(2.47-3.8)	38	2.19	(1.54-3.01)	15	1.80	(1.01-2.98)

INCIDENCE-BASED MORTALITY

	UK			England			Scotland			Wales			Northern Ireland		
Year	Number c	ASMR	95% CI	Number (	ASMI	95% CI	Number (	ASMR	95% CI	Number c	ASMR	95% CI	Number c	ASMR	95% CI
Persons															
1999	657	1.33	(1.23-1.44)	520	1.26	(1.16-1.38)	82	1.95	(1.55-2.42)	43	1.63	(1.18-2.2)	12	1.01	(0.52-1.77)
2000	752	1.52	(1.41-1.63)	621	1.5	(1.38-1.62)	88	2.06	(1.65-2.53)	34	1.32	(0.91-1.85)	9	0.75	(0.34-1.42)
2001	788	1.58	(1.47-1.69)	646	1.55	(1.43-1.67)	85	1.96	(1.57-2.43)	44	1.67	(1.21-2.24)	13	1.03	(0.55-1.76)
2002	921	1.83	(1.71-1.95)	732	1.74	(1.62-1.88)	116	2.63	(2.17-3.16)	46	1.7	(1.24-2.27)	27	2.19	(1.43-3.21)
2003	940	1.86	(1.74-1.98)	761	1.8	(1.67-1.93)	118	2.68	(2.22-3.21)	46	1.71	(1.25-2.28)	15	1.24	(0.68-2.06)
2004	997	1.95	(1.83-2.08)	800	1.87	(1.74-2.01)	130	2.93	(2.45-3.49)	55	1.98	(1.49-2.58)	12	0.96	(0.5-1.68)
2005	1055	2.05	(1.93-2.18)	829	1.92	(1.79-2.06)	145	3.28	(2.76-3.86)	55	2.02	(1.52-2.63)	26	2.07	(1.35-3.03)
2006	1188	2.29	(2.16-2.42)	952	2.19	(2.05-2.34)	154	3.45	(2.93-4.05)	61	2.2	(1.68-2.83)	21	1.61	(0.99-2.47)
2007	1278	2.44	(2.3-2.57)	987	2.26	(2.12-2.4)	184	3.96	(3.41-4.58)	72	2.56	(2-3.22)	35	2.74	(1.9-3.82)
2008	1294	2.42	(2.28-2.55)	1030	2.3	(2.16-2.44)	160	3.43	(2.92-4.01)	79	2.78	(2.2-3.47)	25	1.92	(1.24-2.84)
2009	1375	2.54	(2.41-2.68)	1107	2.45	(2.31-2.6)	150	3.19	(2.7-3.74)	83	2.94	(2.34-3.65)	35	2.52	(1.75-3.51)
2010	1517	2.76	(2.63-2.91)	1225	2.68	(2.53-2.83)	176	3.68	(3.15-4.27)	71	2.41	(1.88-3.04)	45	3.22	(2.34-4.32)
2011	1606	2.90	(2.76-3.04)	1290	2.79	(2.64-2.94)	204	4.26	(3.69-4.89)	79	2.7	(2.14-3.37)	33	2.32	(1.59-3.26)
2012	1852	3.27	(3.12-3.43)	1507	3.19	(3.03-3.35)	197	4.05	(3.5-4.66)	106	3.54	(2.9-4.29)	42	2.86	(2.05-3.87)
2013	1939	3.38	(3.23-3.53)	1509	3.15	(2.99-3.31)	266	5.33	(4.71-6.01)	103	3.39	(2.76-4.11)	61	4.14	(3.17-5.33)
2014	2014	3.43	(3.28-3.58)	1592	3.24	(3.09-3.41)	244	4.83	(4.24-5.48)	123	3.9	(3.24-4.65)	55	3.66	(2.75-4.76)
2015	2076	3.48	(3.34-3.64)	1630	3.28	(3.12-3.44)	272	5.3	(4.69-5.97)	111	3.5	(2.88-4.22)	63	4.11	(3.16-5.27)
2016	2236	3.70	(3.55-3.86)	1783	3.53	(3.37-3.7)	268	5.18	(4.58-5.84)	115	3.62	(2.99-4.35)	70	4.52	(3.52-5.71)
2017	2456	3.98	(3.82-4.14)	1953	3.79	(3.62-3.96)	323	6.12	(5.47-6.83)	131	4.01	(3.35-4.75)	49	3.08	(2.27-4.07)
Men															
1999	463	2.11	(1.92-2.32)	372	2.01	(1.81-2.23)	56	3.16	(2.34-4.17)	26	2.24	(1.45-3.29)	9	1.75	(0.77-3.37)
2000	582	2.66	(2.44-2.89)	483	2.62	(2.39-2.87)	64	3.5	(2.68-4.48)	27	2.47	(1.58-3.66)	8	1.58	(0.67-3.13)
2001	590	2.66	(2.44-2.89)	490	2.63	(2.4-2.88)	65	3.48	(2.67-4.45)	25	2.16	(1.39-3.2)	10	1.78	(0.84-3.29)
2002	686	3.12	(2.88-3.36)	548	2.98	(2.73-3.25)	90	4.65	(3.72-5.73)	30	2.47	(1.66-3.53)	18	3.75	(2.06-6.15)
2003	725	3.24	(3-3.49)	584	3.1	(2.85-3.37)	93	4.93	(3.96-6.06)	36	3.13	(2.14-4.41)	12	2.55	(1.14-4.72)
2004	757	3.34	(3.1-3.59)	609	3.21	(2.96-3.48)	98	5.22	(4.22-6.38)	43	3.42	(2.47-4.62)	7	1.13	(0.45-2.34)
2005	824	3.60	(3.35-3.86)	652	3.39	(3.13-3.67)	110	5.67	(4.62-6.87)	40	3.3	(2.35-4.5)	22	4.07	(2.52-6.21)
2006	927	4.01	(3.75-4.28)	752	3.88	(3.6-4.17)	119	6.15	(5.07-7.38)	43	3.47	(2.49-4.71)	13	2.23	(1.17-3.85)
2007	992	4.19	(3.93-4.46)	764	3.87	(3.59-4.15)	149	7.29	(6.14-8.58)	52	4.01	(2.99-5.27)	27	4.72	(3.09-6.9)
2008	1013	4.15	(3.89-4.42)	809	3.95	(3.68-4.23)	122	5.85	(4.85-7)	61	4.75	(3.63-6.11)	21	3.51	(2.16-5.37)
2009	1056	4.31	(4.05-4.58)	850	4.13	(3.86-4.43)	120	5.75	(4.74-6.9)	62	4.92	(3.74-6.35)	24	3.77	(2.39-5.64)
2010	1188	4.77	(4.5-5.06)	953	4.59	(4.3-4.89)	149	6.97	(5.89-8.2)	54	3.92	(2.94-5.12)	32	5.27	(3.53-7.54)
2011	1234	4.88	(4.61-5.16)	980	4.62	(4.33-4.92)	159	7.58	(6.4-8.9)	64	4.72	(3.62-6.04)	31	4.93	(3.33-7.03)
2012	1435	5.52	(5.24-5.82)	1170	5.37	(5.07-5.69)	153	7.02	(5.93-8.25)	80	5.85	(4.62-7.29)	32	4.79	(3.26-6.79)
2013	1507	5.72	(5.43-6.02)	1155	5.24	(4.94-5.55)	218	9.72	(8.45-11.12)	90	6.41	(5.14-7.89)	44	6.75	(4.86-9.12)
2014	1581	5.88	(5.6-6.19)	1232	5.48	(5.18-5.8)	203	9.09	(7.85-10.46)	102	6.94	(5.64-8.43)	44	6.51	(4.69-8.8)
2015	1611	5.89	(5.6-6.19)	1269	5.56	(5.25-5.87)	211	9.08	(7.88-10.41)	86	5.77	(4.6-7.13)	45	6.56	(4.75-8.83)
2016	1754	6.30	(6-6.6)	1400	6	(5.69-6.33)	213	9.09	(7.89-10.41)	94	6.32	(5.1-7.74)	47	6.54	(4.79-8.72)
2017	1918	6.74	(6.44-7.05)	1528	6.41	(6.09-6.74)	244	10.33	(9.05-11.73)	106	6.96	(5.69-8.43)	40	5.55	(3.95-7.57)
Women															
1999	194	0.69	(0.6-0.8)	148	0.64	(0.54-0.75)	26	1.04	(0.68-1.52)	17	1.12	(0.65-1.8)	3	0.44	(0.09-1.28)
2000	170	0.61	(0.52-0.71)	138	0.6	(0.5-0.71)	24	0.98	(0.63-1.46)	7	0.46	(0.18-0.95)	1	0.16	(0-0.88)
2001	198	0.70	(0.61-0.81)	156	0.66	(0.56-0.78)	20	0.81	(0.49-1.25)	19	1.28	(0.76-2)	3	0.44	(0.09-1.28)
2002	235	0.84	(0.73-0.95)	184	0.78	(0.67-0.9)	26	1.06	(0.69-1.56)	16	1.1	(0.63-1.8)	9	1.26	(0.57-2.39)
2003	215	0.74	(0.65-0.85)	177	0.73	(0.63-0.85)	25	1	(0.64-1.47)	10	0.62	(0.29-1.14)	3	0.46	(0.1-1.35)
2004	240	0.84	(0.74-0.96)	191	0.8	(0.69-0.92)	32	1.3	(0.89-1.84)	12	0.77	(0.39-1.34)	5	0.75	(0.24-1.74)
2005	231	0.81	(0.71-0.92)	177	0.74	(0.63-0.85)	35	1.41	(0.98-1.97)	15	1	(0.56-1.66)	4	0.6	(0.16-1.53)
2006	261	0.89	(0.79-1.01)	200	0.82	(0.71-0.94)	35	1.37	(0.95-1.91)	18	1.21	(0.71-1.92)	8	1.05	(0.45-2.09)
2007	286	0.99	(0.88-1.11)	223	0.92	(0.8-1.05)	35	1.37	(0.95-1.91)	20	1.31	(0.8-2.03)	8	1.14	(0.49-2.25)
2008	281	0.95	(0.84-1.07)	221	0.89	(0.77-1.01)	38	1.49	(1.05-2.04)	18	1.18	(0.7-1.87)	4	0.51	(0.14-1.32)
2009	319	1.07	(0.95-1.2)	257	1.03	(0.9-1.16)	30	1.16	(0.78-1.66)	21	1.36	(0.84-2.08)	11	1.46	(0.73-2.63)
2010	329	1.09	(0.98-1.22)	272	1.09	(0.96-1.23)	27	1.02	(0.67-1.48)	17	1.03	(0.6-1.66)	13	1.67	(0.88-2.86)
2011	372	1.22	(1.1-1.36)	310	1.22	(1.09-1.37)	45	1.68	(1.22-2.25)	15	0.95	(0.53-1.57)	2	0.25	(0.03-0.91)
2012	417	1.36	(1.23-1.49)	337	1.32	(1.18-1.47)	44	1.65	(1.2-2.21)	26	1.58	(1.03-2.32)	10	1.15	(0.55-2.11)
2013	432	1.38	(1.25-1.52)	354	1.35	(1.22-1.5)	48	1.77	(1.31-2.35)	13	0.79	(0.41-1.35)	17	2.04	(1.18-3.27)
2014	433	1.36	(1.23-1.49)	360	1.35	(1.21-1.5)	41	1.47	(1.05-2)	21	1.22	(0.76-1.87)	11	1.38	(0.69-2.48)
2015	465	1.44	(1.31-1.57)	361	1.34	(1.2-1.49)	61	2.15	(1.64-2.76)	25	1.49	(0.96-2.21)	18	2.16	(1.28-3.42)
2016	482	1.48	(1.35-1.62)	383	1.4	(1.27-1.55)	55	1.96	(1.47-2.56)	21	1.22	(0.75-1.86)	23	2.71	(1.71-4.08)
2017	538	1.61	(1.48-1.76)	425	1.53	(1.38-1.68)	79	2.74	(2.17-3.42)	25	1.41	(0.91-2.09)	9	1.05	(0.48-2)

ASR, Age-standardised Rate; ASMR, Age-standardised Mortality Rate; CI, Confidence Interval

Table S4 Intrahepatic cholangiocarcinoma incidence and incidence-based mortality rates, by nation, sex, subtype and year

INCIDENCE

	UK			England			Scotland			Wales			Northern Ireland		
Year	Number of Cases	ASR	95% CI	r of Cases	ASR	95% CI	Number of Cases	ASR	95% CI	r of Cases	ASR	95% CI	Number of Cases	ASR	95% CI
Persons															
1997	875	1.84	(1.72-1.96)	712	1.79	(1.66-1.92)	81	1.98	(1.57-2.46)	46	1.78	(1.3-2.38)	36	3.16	(2.21-4.37)
1998	919	1.92	(1.79-2.05)	729	1.81	(1.68-1.95)	117	2.97	(2.45-3.57)	46	1.75	(1.28-2.34)	27	2.32	(1.53-3.38)
1999	858	1.78	(1.66-1.9)	676	1.67	(1.54-1.8)	106	2.58	(2.11-3.13)	55	2.13	(1.6-2.77)	21	1.92	(1.18-2.95)
2000	1058	2.18	(2.05-2.31)	850	2.09	(1.95-2.24)	113	2.65	(2.19-3.19)	68	2.66	(2.06-3.37)	27	2.25	(1.48-3.27)
2001	1014	2.06	(1.93-2.19)	850	2.05	(1.92-2.2)	100	2.4	(1.95-2.92)	63	2.4	(1.84-3.08)	1	0.08	(0-0.47)
2002	1055	2.12	(2-2.26)	874	2.11	(1.97-2.25)	91	2.11	(1.7-2.59)	62	2.35	(1.8-3.01)	28	2.42	(1.6-3.51)
2003	1076	2.15	(2.02-2.28)	882	2.11	(1.97-2.25)	105	2.49	(2.03-3.02)	73	2.68	(2.1-3.37)	16	1.37	(0.78-2.23)
2004	1204	2.37	(2.24-2.51)	987	2.33	(2.18-2.48)	119	2.81	(2.32-3.37)	81	2.93	(2.33-3.65)	17	1.37	(0.79-2.21)
2005	1269	2.48	(2.35-2.62)	1035	2.42	(2.27-2.57)	131	3.02	(2.53-3.59)	83	3.04	(2.42-3.77)	20	1.63	(0.99-2.52)
2006	1417	2.75	(2.61-2.9)	1172	2.72	(2.57-2.88)	121	2.74	(2.27-3.28)	83	3.06	(2.44-3.8)	41	3.19	(2.28-4.34)
2007	1382	2.63	(2.49-2.77)	1133	2.58	(2.43-2.74)	129	2.89	(2.4-3.43)	104	3.63	(2.96-4.41)	16	1.15	(0.65-1.87)
2008	1507	2.83	(2.68-2.97)	1264	2.83	(2.68-2.99)	111	2.42	(1.98-2.92)	109	3.87	(3.18-4.68)	23	1.68	(1.06-2.54)
2009	1644	3.05	(2.9-3.2)	1400	3.11	(2.94-3.27)	124	2.67	(2.21-3.18)	98	3.4	(2.75-4.15)	22	1.6	(1-2.43)
2010	1602	2.92	(2.78-3.07)	1386	3.03	(2.87-3.19)	122	2.58	(2.14-3.08)	82	2.81	(2.23-3.49)	12	0.87	(0.44-1.54)
2011	1743	3.14	(3-3.29)	1486	3.2	(3.04-3.37)	135	2.9	(2.43-3.44)	94	3.2	(2.59-3.92)	28	2.01	(1.33-2.92)
2012	1776	3.15	(3-3.3)	1472	3.12	(2.96-3.29)	169	3.55	(3.03-4.13)	112	3.76	(3.09-4.52)	23	1.54	(0.97-2.31)
2013	1883	3.26	(3.12-3.41)	1539	3.19	(3.03-3.35)	190	3.86	(3.33-4.45)	125	4.08	(3.39-4.86)	29	2.05	(1.37-2.96)
2014	1895	3.22	(3.08-3.37)	1559	3.16	(3.01-3.32)	194	3.88	(3.35-4.46)	109	3.54	(2.9-4.27)	33	2.18	(1.5-3.07)
2015	2104	3.52	(3.37-3.68)	1715	3.44	(3.27-3.6)	218	4.26	(3.71-4.87)	135	4.29	(3.6-5.08)	36	2.37	(1.65-3.28)
2016	2068	3.41	(3.27-3.56)	1754	3.46	(3.29-3.62)	173	3.36	(2.88-3.9)	103	3.27	(2.67-3.97)	38	2.51	(1.77-3.45)
2017	2053	3.33	(3.18-3.47)	1728	3.34	(3.19-3.51)	189	3.63	(3.13-4.19)	98	3.01	(2.45-3.68)	38	2.37	(1.68-3.26)
Men															
1997	411	2.04	(1.84-2.25)	340	2.02	(1.81-2.26)	36	2	(1.38-2.79)	18	1.62	(0.95-2.57)	17	3.63	(2.1-5.83)
1998	432	2.2	(1.99-2.43)	336	2.01	(1.79-2.25)	54	3.88	(2.77-5.23)	28	2.54	(1.68-3.69)	14	2.97	(1.59-5.02)
1999	410	2.08	(1.88-2.3)	331	1.99	(1.77-2.22)	52	3.25	(2.4-4.29)	24	2.5	(1.55-3.79)	3	0.67	(0.11-2.02)
2000	483	2.39	(2.17-2.62)	396	2.32	(2.09-2.57)	52	2.98	(2.2-3.94)	24	2.64	(1.59-4.07)	11	2.09	(1.03-3.77)
2001	476	2.4	(2.17-2.63)	409	2.45	(2.2-2.71)	35	2.03	(1.4-2.84)	32	3.21	(2.1-4.65)	0	0	(-)
2002	488	2.37	(2.16-2.6)	405	2.35	(2.12-2.6)	39	2.22	(1.54-3.07)	29	2.76	(1.79-4.05)	15	3.12	(1.71-5.19)
2003	526	2.52	(2.3-2.75)	431	2.48	(2.24-2.73)	51	2.96	(2.16-3.93)	34	2.89	(1.96-4.08)	10	1.83	(0.87-3.38)
2004	548	2.54	(2.33-2.77)	456	2.52	(2.29-2.77)	51	3.06	(2.24-4.08)	33	2.63	(1.8-3.7)	8	1.54	(0.64-3.08)
2005	607	2.76	(2.54-3)	499	2.71	(2.48-2.97)	61	3.25	(2.46-4.2)	36	3.2	(2.19-4.49)	11	1.99	(0.98-3.58)
2006	683	3.15	(2.91-3.41)	559	3.08	(2.82-3.35)	62	3.43	(2.58-4.45)	40	3.41	(2.41-4.67)	22	4.2	(2.52-6.51)
2007	613	2.73	(2.52-2.97)	502	2.66	(2.43-2.92)	56	3.12	(2.32-4.11)	49	4.05	(2.97-5.38)	6	1.05	(0.37-2.32)
2008	732	3.21	(2.97-3.45)	615	3.23	(2.97-3.5)	52	2.65	(1.95-3.51)	54	4.39	(3.26-5.79)	11	1.63	(0.79-2.95)
2009	772	3.34	(3.1-3.59)	652	3.37	(3.11-3.65)	60	3.04	(2.31-3.92)	49	3.94	(2.88-5.25)	11	1.91	(0.93-3.47)
2010	731	3.09	(2.86-3.33)	632	3.19	(2.94-3.45)	56	2.76	(2.06-3.61)	34	2.66	(1.83-3.74)	9	1.62	(0.65-3.22)
2011	838	3.48	(3.25-3.73)	724	3.58	(3.32-3.86)	56	2.81	(2.1-3.67)	43	3.44	(2.46-4.68)	15	2.41	(1.33-4.01)
2012	837	3.37	(3.14-3.61)	695	3.35	(3.1-3.61)	70	3.33	(2.59-4.22)	54	4.14	(3.08-5.43)	18	2.6	(1.53-4.13)
2013	894	3.53	(3.3-3.78)	736	3.46	(3.21-3.72)	92	4.49	(3.59-5.55)	53	3.77	(2.82-4.94)	13	2.7	(1.33-4.76)
2014	907	3.49	(3.26-3.73)	736	3.37	(3.13-3.63)	95	4.42	(3.56-5.42)	63	4.57	(3.5-5.86)	13	1.97	(1.04-3.39)
2015	1005	3.78	(3.54-4.02)	811	3.65	(3.4-3.91)	101	4.49	(3.63-5.48)	75	5.2	(4.07-6.53)	18	2.63	(1.54-4.17)
2016	1002	3.69	(3.46-3.92)	859	3.77	(3.52-4.03)	79	3.58	(2.81-4.48)	52	3.57	(2.66-4.69)	12	1.71	(0.88-3)
2017	991	3.6	(3.38-3.83)	843	3.65	(3.41-3.91)	89	3.92	(3.13-4.83)	41	2.79	(1.99-3.79)	18	2.33	(1.37-3.69)
Women															
1997	464	1.63	(1.49-1.79)	372	1.57	(1.41-1.74)	45	1.82	(1.33-2.44)	28	1.86	(1.23-2.69)	19	2.73	(1.64-4.26)
1998	487	1.74	(1.59-1.9)	393	1.68	(1.52-1.86)	63	2.58	(1.98-3.3)	18	1.21	(0.71-1.91)	13	1.98	(1.05-3.39)
1999	448	1.6	(1.45-1.76)	345	1.47	(1.32-1.63)	54	2.24	(1.68-2.92)	31	2.11	(1.43-3)	18	2.72	(1.61-4.3)
2000	575	2.03	(1.87-2.21)	454	1.92	(1.75-2.11)	61	2.45	(1.87-3.15)	44	2.83	(2.05-3.81)	16	2.34	(1.34-3.81)
2001	538	1.88	(1.72-2.04)	441	1.83	(1.67-2.02)	65	2.65	(2.04-3.38)	31	2.07	(1.4-2.94)	1	0.16	(0-0.87)
2002	567	1.96	(1.8-2.13)	469	1.94	(1.77-2.12)	52	2.01	(1.5-2.64)	33	2.19	(1.5-3.09)	13	1.91	(1.01-3.28)
2003	550	1.89	(1.73-2.05)	451	1.84	(1.68-2.02)	54	2.19	(1.65-2.86)	39	2.52	(1.79-3.46)	6	0.9	(0.33-1.97)
2004	656	2.24	(2.07-2.42)	531	2.17	(1.99-2.36)	68	2.67	(2.07-3.39)	48	3.06	(2.25-4.07)	9	1.29	(0.58-2.46)
2005	662	2.24	(2.07-2.42)	536	2.17	(1.99-2.36)	70	2.73	(2.13-3.46)	47	3.05	(2.24-4.07)	9	1.22	(0.55-2.32)
2006	734	2.5	(2.32-2.69)	613	2.5	(2.3-2.7)	59	2.3	(1.75-2.97)	43	2.86	(2.07-3.87)	19	2.55	(1.53-3.98)
2007	769	2.58	(2.4-2.77)	631	2.54	(2.34-2.75)	73	2.84	(2.23-3.58)	55	3.3	(2.47-4.3)	10	1.27	(0.61-2.34)
2008	775	2.55	(2.37-2.74)	649	2.55	(2.36-2.76)	59	2.25	(1.71-2.91)	55	3.41	(2.56-4.45)	12	1.66	(0.85-2.9)
2009	872	2.84	(2.66-3.04)	748	2.93	(2.72-3.15)	64	2.39	(1.84-3.06)	49	2.91	(2.15-3.86)	11	1.43	(0.71-2.57)
2010	871	2.8	(2.61-2.99)	754	2.9	(2.69-3.12)	66	2.46	(1.9-3.13)	48	2.89	(2.12-3.84)	3	0.38	(0.08-1.13)
2011	905	2.89	(2.71-3.09)	762	2.92	(2.72-3.14)	79	2.92	(2.31-3.65)	51	3.1	(2.3-4.09)	13	1.62	(0.86-2.78)
2012	939	2.97	(2.78-3.17)	777	2.95	(2.75-3.17)	99	3.57	(2.9-4.36)	58	3.47	(2.63-4.5)	5	0.56	(0.18-1.3)
2013	989	3.05	(2.86-3.25)	803	2.98	(2.77-3.19)	98	3.46	(2.81-4.22)	72	4.13	(3.22-5.21)	16	1.89	(1.07-3.07)
2014	988	3.01	(2.82-3.2)	823	2.99	(2.79-3.2)	99	3.52	(2.86-4.29)	46	2.72	(1.98-3.63)	20	2.37	(1.44-3.67)
2015	1099	3.29	(3.1-3.49)	904	3.24	(3.03-3.46)	117	4.06	(3.35-4.86)	60	3.42	(2.61-4.42)	18	2.1	(1.24-3.33)
2016	1066	3.19	(3-3.39)	895	3.19	(2.98-3.41)	94	3.29	(2.65-4.02)	51	2.98	(2.22-3.93)	26	3.08	(2.01-4.52)
2017	1062	3.13	(2.94-3.32)	885	3.12	(2.92-3.34)	100	3.4	(2.76-4.14)	57	3.15	(2.38-4.09)	20	2.27	(1.38-3.51)

	UK			England			Scotland			Wales			Northern Ireland		
	**INCIDENCE-BASED MORTALITY**														
**Year**	**Number of Cases**	**ASMI**	**95% CI**	**Number of Cases**	**ASMR**	**95% CI**	**Number of Cases**	**ASMF**	**95% CI**	**Number of Cases**	**ASMF**	**95% CI**	**Number of Cases**	**ASMR**	**95% CI**
Persons															
1999	689	1.43	(1.32-1.54)	549	1.36	(1.25-1.48)	84	2.09	(1.66-2.59)	40	1.55	(1.11-2.12)	16	1.43	(0.81-2.33)
2000	777	1.6	(1.49-1.72)	627	1.54	(1.42-1.67)	81	1.92	(1.52-2.38)	50	1.99	(1.47-2.63)	19	1.6	(0.96-2.5)
2001	868	1.77	(1.65-1.89)	725	1.77	(1.64-1.9)	81	1.92	(1.52-2.39)	55	2.09	(1.57-2.72)	7	0.57	(0.23-1.17)
2002	856	1.73	(1.61-1.85)	722	1.74	(1.62-1.87)	70	1.63	(1.27-2.06)	47	1.77	(1.3-2.36)	17	1.48	(0.86-2.38)
2003	902	1.81	(1.69-1.93)	754	1.8	(1.68-1.94)	79	1.85	(1.46-2.31)	55	2.06	(1.55-2.69)	14	1.24	(0.67-2.08)
2004	982	1.94	(1.82-2.06)	786	1.85	(1.72-1.98)	111	2.62	(2.15-3.16)	70	2.58	(2.01-3.27)	15	1.16	(0.65-1.92)
2005	1048	2.06	(1.93-2.18)	869	2.04	(1.91-2.18)	95	2.17	(1.75-2.65)	70	2.54	(1.98-3.22)	14	1.11	(0.6-1.88)
2006	1116	2.17	(2.05-2.3)	921	2.14	(2.01-2.29)	111	2.54	(2.08-3.06)	69	2.52	(1.96-3.19)	15	1.12	(0.63-1.86)
2007	1152	2.21	(2.08-2.34)	946	2.17	(2.04-2.32)	95	2.12	(1.71-2.6)	87	3.1	(2.48-3.83)	24	1.83	(1.17-2.72)
2008	1231	2.3	(2.17-2.43)	1038	2.32	(2.18-2.47)	92	2.01	(1.61-2.47)	85	2.97	(2.37-3.68)	16	1.11	(0.63-1.81)
2009	1389	2.58	(2.45-2.72)	1178	2.62	(2.47-2.77)	107	2.32	(1.89-2.8)	87	3.04	(2.43-3.76)	17	1.3	(0.75-2.09)
2010	1307	2.39	(2.26-2.52)	1153	2.52	(2.38-2.67)	86	1.82	(1.45-2.25)	58	1.98	(1.5-2.56)	10	0.76	(0.36-1.4)
2011	1449	2.61	(2.47-2.74)	1240	2.67	(2.52-2.82)	109	2.31	(1.89-2.79)	76	2.58	(2.03-3.23)	24	1.66	(1.06-2.47)
2012	1504	2.67	(2.53-2.81)	1283	2.72	(2.57-2.87)	136	2.86	(2.39-3.38)	69	2.33	(1.81-2.95)	16	1.1	(0.62-1.78)
2013	1542	2.68	(2.55-2.82)	1242	2.58	(2.44-2.73)	160	3.31	(2.81-3.86)	114	3.69	(3.04-4.44)	26	1.85	(1.2-2.72)
2014	1589	2.7	(2.57-2.83)	1312	2.66	(2.52-2.81)	160	3.21	(2.73-3.76)	95	3.09	(2.5-3.78)	22	1.46	(0.91-2.22)
2015	1681	2.81	(2.68-2.95)	1365	2.73	(2.58-2.88)	164	3.24	(2.76-3.77)	118	3.77	(3.11-4.51)	34	2.22	(1.53-3.1)
2016	1719	2.84	(2.7-2.97)	1425	2.8	(2.66-2.95)	187	3.64	(3.13-4.2)	75	2.39	(1.88-3)	32	2.09	(1.43-2.96)
2017	1851	3	(2.86-3.14)	1562	3.02	(2.87-3.17)	177	3.4	(2.91-3.94)	90	2.79	(2.24-3.43)	22	1.41	(0.88-2.14)
Men															
1999	341	1.75	(1.56-1.96)	270	1.62	(1.43-1.84)	45	3.07	(2.15-4.23)	22	2.32	(1.4-3.58)	4	0.78	(0.21-1.99)
2000	346	1.73	(1.54-1.93)	290	1.71	(1.51-1.92)	32	1.79	(1.21-2.55)	17	2.07	(1.11-3.45)	7	1.42	(0.54-2.98)
2001	392	2.03	(1.82-2.26)	337	2.08	(1.85-2.33)	26	1.57	(1.01-2.33)	24	2.42	(1.48-3.7)	5	0.88	(0.28-2.06)
2002	383	1.86	(1.67-2.07)	321	1.86	(1.65-2.08)	28	1.64	(1.08-2.39)	25	2.3	(1.45-3.44)	9	1.95	(0.88-3.73)
2003	439	2.07	(1.87-2.28)	373	2.1	(1.88-2.33)	39	2.06	(1.45-2.84)	21	1.98	(1.16-3.11)	6	1.28	(0.45-2.83)
2004	447	2.09	(1.9-2.3)	361	2.01	(1.81-2.24)	46	2.86	(2.04-3.88)	30	2.45	(1.64-3.51)	10	1.77	(0.84-3.26)
2005	483	2.22	(2.02-2.44)	405	2.23	(2.02-2.47)	44	2.34	(1.69-3.15)	28	2.34	(1.52-3.42)	6	1.19	(0.42-2.62)
2006	536	2.45	(2.24-2.67)	443	2.42	(2.19-2.66)	50	2.72	(1.99-3.62)	32	2.72	(1.84-3.88)	11	1.7	(0.84-3.06)
2007	523	2.37	(2.16-2.58)	427	2.3	(2.08-2.54)	51	2.78	(2.02-3.71)	38	3.27	(2.27-4.54)	7	1.28	(0.5-2.65)
2008	566	2.49	(2.28-2.71)	475	2.49	(2.26-2.73)	40	2.15	(1.51-2.97)	44	3.68	(2.63-5)	7	1.03	(0.4-2.15)
2009	652	2.84	(2.62-3.08)	552	2.87	(2.63-3.13)	50	2.61	(1.91-3.48)	40	3.28	(2.31-4.51)	10	1.77	(0.83-3.28)
2010	585	2.49	(2.29-2.71)	516	2.63	(2.4-2.87)	41	2.1	(1.48-2.87)	22	1.64	(1.03-2.49)	6	1.06	(0.38-2.34)
2011	677	2.81	(2.6-3.03)	578	2.86	(2.63-3.11)	48	2.34	(1.7-3.13)	37	2.84	(1.98-3.93)	14	2.2	(1.18-3.72)
2012	686	2.78	(2.57-3)	595	2.87	(2.64-3.12)	50	2.41	(1.78-3.18)	33	2.5	(1.7-3.54)	8	1.18	(0.5-2.34)
2013	712	2.85	(2.64-3.07)	575	2.73	(2.51-2.96)	76	3.86	(3.01-4.86)	44	3.13	(2.26-4.21)	17	3.12	(1.73-5.14)
2014	744	2.86	(2.65-3.07)	608	2.78	(2.56-3.02)	71	3.23	(2.51-4.09)	55	3.99	(3-5.21)	10	1.62	(0.73-3.06)
2015	773	2.92	(2.72-3.14)	625	2.82	(2.6-3.05)	74	3.39	(2.64-4.28)	56	4.05	(3.04-5.28)	18	2.62	(1.54-4.16)
2016	805	2.97	(2.76-3.18)	668	2.94	(2.72-3.17)	90	4.06	(3.24-5.03)	35	2.38	(1.65-3.32)	12	1.68	(0.86-2.95)
2017	872	3.15	(2.95-3.37)	743	3.2	(2.97-3.44)	70	3.05	(2.37-3.86)	52	3.56	(2.65-4.67)	7	1.00	(0.39-2.07)
Women															
1999	348	1.22	(1.1-1.36)	279	1.17	(1.04-1.32)	39	1.6	(1.13-2.18)	18	1.17	(0.69-1.86)	12	1.82	(0.94-3.19)
2000	431	1.52	(1.37-1.67)	337	1.41	(1.27-1.58)	49	1.99	(1.47-2.64)	33	2.13	(1.46-3)	12	1.77	(0.92-3.1)
2001	476	1.66	(1.52-1.82)	388	1.62	(1.46-1.79)	55	2.23	(1.68-2.91)	31	2.03	(1.37-2.89)	2	0.31	(0.04-1.12)
2002	473	1.63	(1.48-1.78)	401	1.65	(1.49-1.83)	42	1.66	(1.19-2.24)	22	1.39	(0.87-2.11)	8	1.19	(0.51-2.34)
2003	463	1.6	(1.46-1.76)	381	1.57	(1.42-1.74)	40	1.61	(1.15-2.2)	34	2.26	(1.56-3.17)	8	1.21	(0.52-2.38)
2004	535	1.82	(1.67-1.98)	425	1.72	(1.56-1.9)	65	2.55	(1.97-3.26)	40	2.64	(1.88-3.6)	5	0.7	(0.22-1.65)
2005	565	1.91	(1.75-2.07)	464	1.87	(1.7-2.05)	51	1.98	(1.47-2.61)	42	2.69	(1.93-3.64)	8	1.08	(0.46-2.13)
2006	580	1.97	(1.81-2.13)	478	1.94	(1.76-2.12)	61	2.39	(1.83-3.07)	37	2.39	(1.68-3.31)	4	0.54	(0.15-1.38)
2007	629	2.12	(1.95-2.29)	519	2.1	(1.92-2.29)	44	1.7	(1.23-2.28)	49	3.04	(2.24-4.03)	17	2.26	(1.31-3.63)
2008	665	2.17	(2.01-2.34)	563	2.2	(2.02-2.39)	52	1.94	(1.45-2.55)	41	2.47	(1.76-3.36)	9	1.15	(0.52-2.2)
2009	737	2.4	(2.23-2.59)	626	2.44	(2.25-2.65)	57	2.17	(1.64-2.81)	47	2.84	(2.08-3.79)	7	0.9	(0.36-1.87)
2010	722	2.31	(2.15-2.49)	637	2.45	(2.26-2.65)	45	1.69	(1.23-2.26)	36	2.13	(1.49-2.96)	4	0.54	(0.14-1.38)
2011	772	2.46	(2.29-2.64)	662	2.53	(2.34-2.74)	61	2.21	(1.68-2.84)	39	2.35	(1.66-3.22)	10	1.23	(0.59-2.27)
2012	818	2.58	(2.4-2.76)	688	2.6	(2.4-2.8)	86	3.13	(2.5-3.87)	36	2.17	(1.51-3.01)	8	0.95	(0.41-1.87)
2013	830	2.56	(2.38-2.74)	667	2.47	(2.28-2.67)	84	3	(2.39-3.71)	70	3.96	(3.08-5.01)	9	1.06	(0.48-2.01)
2014	845	2.56	(2.39-2.74)	704	2.55	(2.36-2.75)	89	3.13	(2.51-3.85)	40	2.43	(1.73-3.32)	12	1.38	(0.71-2.41)
2015	908	2.72	(2.54-2.9)	740	2.64	(2.46-2.84)	90	3.14	(2.52-3.87)	62	3.58	(2.74-4.6)	16	1.85	(1.05-3)
2016	914	2.72	(2.54-2.9)	757	2.68	(2.49-2.88)	97	3.41	(2.76-4.16)	40	2.32	(1.65-3.17)	20	2.31	(1.41-3.58)
2017	979	2.87	(2.69-3.06)	819	2.87	(2.68-3.08)	107	3.65	(2.99-4.41)	38	2.09	(1.48-2.88)	15	1.77	(0.99-2.93)

ASR, Age-standardised Rate; ASMR, Age-standardised Mortality Rate; CI, Confidence Interval

Table S5 Other and unspecified liver tumour incidence and incidence-based mortality rates, by nation, sex, subtype and year

	INCIDENCE														
	UK			England			Scotland			Wales			Northern Ireland		
Year	Number of Cases	ASR	95% CI	Number of Cases	ASR	95% CI	Number of Cases	ASR	95% CI	Number of Cases	ASR	95% CI	Number of Cases	ASR	95% CI
Persons															
1997	367	0.76	(0.68-0.84	313	0.77	(0.69-0.86)	19	0.45	(0.27-0.7)	27	1.06	(0.7-1.54)	8	0.69	(0.3-1.36)
1998	359	0.74	(0.66-0.82	291	0.72	(0.64-0.81)	30	0.76	(0.5-1.09)	34	1.28	(0.88-1.79	4	0.25	(0.06-0.65)
1999	415	0.85	(0.77-0.94	350	0.86	(0.77-0.96)	28	0.65	(0.43-0.95)	32	1.21	(0.82-1.71	5	0.37	(0.11-0.89)
2000	390	0.79	(0.71-0.87	327	0.79	(0.71-0.88)	24	0.58	(0.37-0.87)	23	0.85	(0.54-1.29	16	1.35	(0.77-2.2)
2001	326	0.64	(0.58-0.72	277	0.65	(0.58-0.74)	23	0.52	(0.33-0.78)	23	0.87	(0.55-1.3)	3	0.2	(0.04-0.6)
2002	350	0.69	(0.62-0.76	310	0.72	(0.64-0.81)	14	0.33	(0.18-0.56)	23	0.88	(0.55-1.33	3	0.19	(0.04-0.56)
2003	345	0.68	(0.61-0.75	286	0.67	(0.6-0.76)	24	0.58	(0.37-0.87)	24	0.85	(0.54-1.27	11	0.76	(0.37-1.37)
2004	328	0.64	(0.57-0.71	280	0.65	(0.58-0.73)	25	0.59	(0.38-0.88)	13	0.45	(0.24-0.77	10	0.81	(0.39-1.49)
2005	351	0.68	(0.61-0.76	303	0.7	(0.63-0.79)	23	0.52	(0.33-0.79)	16	0.55	(0.31-0.9)	9	0.79	(0.36-1.5)
2006	365	0.69	(0.62-0.77	316	0.72	(0.64-0.8)	25	0.57	(0.37-0.84)	16	0.56	(0.32-0.92	8	0.63	(0.27-1.24)
2007	415	0.78	(0.71-0.86	366	0.82	(0.74-0.91)	31	0.71	(0.48-1.01)	8	0.28	(0.12-0.54	10	0.68	(0.31-1.26)
2008	503	0.94	(0.86-1.03	455	1.02	(0.93-1.12)	24	0.52	(0.33-0.78)	18	0.62	(0.37-0.98	6	0.41	(0.15-0.9)
2009	512	0.94	(0.86-1.02	475	1.04	(0.95-1.14)	16	0.36	(0.2-0.58)	11	0.38	(0.19-0.68	10	0.7	(0.33-1.29)
2010	582	1.05	(0.96-1.13	518	1.11	(1.02-1.21)	25	0.54	(0.35-0.8)	13	0.45	(0.24-0.77	26	1.72	(1.12-2.54)
2011	504	0.88	(0.81-0.97	430	0.9	(0.82-0.99)	24	0.48	(0.31-0.72)	27	0.9	(0.59-1.32	23	1.62	(1.01-2.45)
2012	702	1.22	(1.13-1.32	659	1.38	(1.27-1.48)	14	0.3	(0.16-0.5)	21	0.68	(0.42-1.05	8	0.48	(0.2-0.96)
2013	833	1.44	(1.34-1.54	786	1.62	(1.51-1.74)	20	0.41	(0.25-0.63)	21	0.66	(0.41-1.02	6	0.4	(0.14-0.88)
2014	784	1.32	(1.23-1.41	717	1.44	(1.33-1.55)	27	0.56	(0.37-0.82)	29	0.92	(0.62-1.32	11	0.77	(0.38-1.37)
2015	611	1.01	(0.93-1.09	551	1.08	(1-1.18)	15	0.3	(0.17-0.5)	31	1	(0.68-1.42	14	0.95	(0.52-1.59)
2016	579	0.94	(0.87-1.02	527	1.02	(0.94-1.11)	15	0.3	(0.17-0.5)	26	0.81	(0.53-1.19	11	0.69	(0.34-1.24)
2017	544	0.87	(0.8-0.94)	492	0.94	(0.86-1.02)	19	0.37	(0.22-0.57)	21	0.65	(0.4-1)	12	0.76	(0.39-1.33)
Men															
1997	201	0.98	(0.85-1.14	171	0.98	(0.84-1.15)	10	0.57	(0.26-1.06)	15	1.58	(0.81-2.71	5	1	(0.32-2.35)
1998	218	1.05	(0.91-1.21	179	1.02	(0.87-1.18)	21	1.55	(0.84-2.53)	15	1.23	(0.68-2.04	3	0.36	(0.06-1.09)
1999	263	1.32	(1.16-1.5)	218	1.31	(1.13-1.51)	19	1.05	(0.62-1.66)	24	2.38	(1.47-3.62	2	0.21	(0.02-0.77)
2000	236	1.12	(0.97-1.27	202	1.14	(0.98-1.31)	10	0.63	(0.25-1.24)	15	1.32	(0.72-2.2)	9	1.69	(0.77-3.22)
2001	176	0.82	(0.69-0.95	147	0.81	(0.68-0.96)	15	0.78	(0.42-1.3)	12	1.14	(0.54-2.07	2	0.27	(0.03-0.96)
2002	200	0.94	(0.81-1.09	173	0.96	(0.82-1.12)	10	0.51	(0.24-0.95)	15	1.64	(0.85-2.8)	2	0.29	(0.04-1.05)
2003	200	0.93	(0.8-1.08)	163	0.91	(0.77-1.07)	13	0.88	(0.43-1.57)	18	1.45	(0.85-2.31	6	0.88	(0.31-1.95)
2004	202	0.92	(0.79-1.06	170	0.91	(0.78-1.07)	16	1.03	(0.57-1.7)	10	0.75	(0.36-1.38	6	1.24	(0.44-2.74)
2005	191	0.85	(0.73-0.99	163	0.87	(0.73-1.01)	14	0.8	(0.42-1.36)	9	0.71	(0.32-1.34	5	1.05	(0.33-2.46)
2006	201	0.92	(0.8-1.07)	175	0.94	(0.8-1.1)	12	0.7	(0.33-1.26)	10	1.07	(0.47-2.04	4	0.74	(0.2-1.91)
2007	258	1.12	(0.98-1.27	230	1.19	(1.03-1.35)	21	1.25	(0.72-1.98)	2	0.16	(0.02-0.59	5	0.7	(0.18-1.74)
2008	296	1.27	(1.12-1.43	270	1.38	(1.22-1.57)	15	0.79	(0.44-1.31)	7	0.51	(0.2-1.06)	4	0.62	(0.17-1.6)
2009	290	1.19	(1.06-1.34	267	1.31	(1.16-1.48)	13	0.64	(0.34-1.1)	4	0.3	(0.08-0.78	6	0.92	(0.33-2.01)
2010	327	1.33	(1.18-1.48	292	1.41	(1.25-1.59)	16	0.78	(0.44-1.27)	8	0.61	(0.26-1.2)	11	1.59	(0.77-2.87)
2011	289	1.17	(1.03-1.31	248	1.19	(1.04-1.35)	18	0.88	(0.52-1.4)	12	0.94	(0.47-1.68	11	2	(0.92-3.7)
2012	424	1.65	(1.49-1.81	393	1.82	(1.64-2.02)	9	0.42	(0.19-0.81)	16	1.13	(0.64-1.84	6	0.95	(0.33-2.11)
2013	485	1.92	(1.75-2.11	456	2.16	(1.96-2.37)	13	0.62	(0.31-1.08)	12	0.81	(0.41-1.42	4	0.63	(0.17-1.63)
2014	443	1.7	(1.55-1.87	404	1.84	(1.67-2.04)	13	0.73	(0.37-1.29)	20	1.45	(0.87-2.25	6	0.94	(0.34-2.05)
2015	323	1.2	(1.07-1.34	284	1.24	(1.1-1.4)	11	0.61	(0.28-1.12)	19	1.37	(0.81-2.16	9	1.39	(0.63-2.65)
2016	337	1.24	(1.11-1.38	299	1.3	(1.15-1.46)	12	0.63	(0.31-1.12)	19	1.3	(0.78-2.05	7	1.1	(0.4-2.35)
2017	294	1.03	(0.91-1.15	262	1.09	(0.96-1.23)	13	0.58	(0.3-1.01)	12	0.81	(0.42-1.41	7	0.99	(0.39-2.05)
Women															
1997	166	0.59	(0.51-0.69	142	0.61	(0.51-0.72)	9	0.36	(0.17-0.69)	12	0.85	(0.44-1.49	3	0.4	(0.08-1.18)
1998	141	0.49	(0.42-0.58	112	0.47	(0.39-0.57)	9	0.37	(0.17-0.7)	19	1.21	(0.72-1.89	1	0.14	(0-0.8)
1999	152	0.53	(0.45-0.62	132	0.55	(0.46-0.65)	9	0.36	(0.16-0.68)	8	0.53	(0.23-1.04	3	0.43	(0.09-1.26)
2000	154	0.53	(0.45-0.62	125	0.51	(0.43-0.61)	14	0.57	(0.31-0.95)	8	0.5	(0.21-0.98	7	1.02	(0.41-2.11)
2001	150	0.52	(0.44-0.61	130	0.54	(0.45-0.65)	8	0.32	(0.14-0.64)	11	0.67	(0.33-1.2)	1	0.14	(0-0.8)
2002	150	0.5	(0.42-0.59	137	0.55	(0.46-0.65)	4	0.17	(0.05-0.43)	8	0.52	(0.22-1.02	1	0.09	(0-0.52)
2003	145	0.5	(0.42-0.59	123	0.5	(0.42-0.6)	11	0.45	(0.22-0.8)	6	0.41	(0.15-0.89	5	0.62	(0.19-1.45)
2004	126	0.43	(0.36-0.52	110	0.45	(0.37-0.55)	9	0.35	(0.16-0.66)	3	0.22	(0.04-0.64	4	0.53	(0.14-1.36)
2005	160	0.55	(0.46-0.64	140	0.57	(0.48-0.68)	9	0.34	(0.16-0.66)	7	0.41	(0.16-0.86	4	0.59	(0.16-1.51)
2006	164	0.55	(0.47-0.64	141	0.57	(0.48-0.68)	13	0.49	(0.26-0.84)	6	0.33	(0.12-0.72	4	0.52	(0.14-1.34)
2007	157	0.52	(0.44-0.61	136	0.54	(0.45-0.64)	10	0.37	(0.18-0.68)	6	0.39	(0.14-0.84	5	0.68	(0.22-1.6)
2008	207	0.68	(0.59-0.78	185	0.74	(0.63-0.85)	9	0.33	(0.15-0.62)	11	0.66	(0.32-1.18	2	0.2	(0.02-0.74)
2009	222	0.72	(0.63-0.82	208	0.81	(0.7-0.93)	3	0.12	(0.02-0.35)	7	0.38	(0.15-0.78	4	0.48	(0.13-1.24)
2010	255	0.82	(0.73-0.93	226	0.88	(0.76-1)	9	0.33	(0.15-0.63)	5	0.34	(0.11-0.78	15	1.81	(1-3.01)
2011	215	0.66	(0.58-0.76	182	0.67	(0.58-0.78)	6	0.21	(0.08-0.46)	15	0.9	(0.5-1.49)	12	1.43	(0.73-2.51)
2012	278	0.87	(0.77-0.97	266	0.99	(0.88-1.12)	5	0.18	(0.06-0.43)	5	0.29	(0.09-0.69	2	0.19	(0.02-0.68)
2013	348	1.08	(0.97-1.2)	330	1.23	(1.1-1.37)	7	0.25	(0.1-0.52)	9	0.5	(0.23-0.96	2	0.2	(0.02-0.74)
2014	341	1.01	(0.91-1.13	313	1.11	(0.99-1.24)	14	0.5	(0.27-0.84)	9	0.52	(0.24-1)	5	0.59	(0.19-1.38)
2015	288	0.85	(0.76-0.96	267	0.94	(0.83-1.07)	4	0.15	(0.04-0.38)	12	0.66	(0.34-1.16	5	0.61	(0.2-1.43)
2016	242	0.71	(0.62-0.8)	228	0.8	(0.7-0.91)	3	0.1	(0.02-0.29)	7	0.4	(0.16-0.82	4	0.43	(0.12-1.1)
2017	250	0.73	(0.64-0.83	230	0.8	(0.7-0.92)	6	0.21	(0.08-0.46)	9	0.52	(0.24-1)	5	0.5	(0.16-1.18)

INCIDENCE-BASED MORTALITY

Year	UK Number of Cases	UK ASMR	UK 95% CI	England Number of Cases	England ASMR	England 95% CI	Scotland Number of Cases	Scotland ASMR	Scotland 95% CI	Wales Number of Cases	Wales ASMR	Wales 95% CI	Northern Ireland Number of Cases	Northern Ireland ASMR	Northern Ireland 95% CI
Persons															
1999	248	0.51	(0.45-0.58	206	0.51	(0.44-0.59)	21	0.51	(0.31-0.78)	15	0.58	(0.32-0.96	6	0.46	(0.16-1.02)
2000	209	0.43	(0.37-0.49	175	0.43	(0.37-0.5)	14	0.34	(0.19-0.57)	12	0.46	(0.24-0.81	8	0.68	(0.29-1.35)
2001	171	0.35	(0.3-0.4)	151	0.37	(0.31-0.43)	12	0.27	(0.14-0.48)	8	0.3	(0.13-0.58	0	0	(-)
2002	180	0.36	(0.31-0.42	162	0.38	(0.33-0.45)	5	0.12	(0.04-0.29)	13	0.49	(0.26-0.84	0	0	(-)
2003	219	0.44	(0.38-0.5)	186	0.44	(0.38-0.51)	12	0.3	(0.16-0.53)	15	0.53	(0.29-0.87	6	0.45	(0.16-0.99)
2004	190	0.37	(0.32-0.43	166	0.39	(0.33-0.46)	15	0.35	(0.19-0.58)	6	0.2	(0.07-0.45	3	0.2	(0.04-0.6)
2005	212	0.41	(0.36-0.47	174	0.41	(0.35-0.47)	13	0.3	(0.16-0.51)	16	0.56	(0.32-0.91	9	0.76	(0.34-1.46)
2006	191	0.37	(0.32-0.42	162	0.37	(0.32-0.44)	15	0.33	(0.18-0.55)	9	0.33	(0.15-0.62	5	0.33	(0.1-0.79)
2007	239	0.46	(0.4-0.52)	202	0.46	(0.4-0.53)	28	0.65	(0.43-0.94)	6	0.22	(0.08-0.48	3	0.24	(0.05-0.71)
2008	267	0.5	(0.45-0.57	238	0.54	(0.47-0.61)	16	0.34	(0.2-0.56)	9	0.32	(0.14-0.6)	4	0.3	(0.08-0.76)
2009	244	0.45	(0.4-0.51)	219	0.49	(0.42-0.56)	14	0.29	(0.16-0.49)	6	0.22	(0.08-0.48	5	0.35	(0.11-0.83)
2010	277	0.51	(0.45-0.57	244	0.53	(0.47-0.61)	19	0.43	(0.25-0.67)	7	0.24	(0.1-0.49)	7	0.5	(0.2-1.03)
2011	268	0.48	(0.43-0.55	236	0.51	(0.44-0.58)	13	0.28	(0.15-0.47)	9	0.31	(0.14-0.59	10	0.79	(0.38-1.46)
2012	290	0.51	(0.45-0.57	260	0.54	(0.48-0.61)	11	0.24	(0.12-0.42)	8	0.26	(0.11-0.51	11	0.76	(0.37-1.36)
2013	415	0.71	(0.65-0.79	389	0.8	(0.72-0.88)	13	0.27	(0.14-0.46)	8	0.26	(0.11-0.52	5	0.35	(0.11-0.82)
2014	456	0.78	(0.71-0.85	410	0.83	(0.75-0.92)	17	0.36	(0.21-0.58)	21	0.65	(0.4-1)	8	0.58	(0.25-1.15)
2015	336	0.56	(0.5-0.62)	308	0.61	(0.54-0.68)	10	0.21	(0.1-0.38)	13	0.43	(0.23-0.73	5	0.33	(0.11-0.78)
2016	352	0.57	(0.52-0.64	320	0.62	(0.56-0.7)	10	0.21	(0.1-0.38)	13	0.41	(0.22-0.7)	9	0.59	(0.27-1.13)
2017	344	0.56	(0.5-0.62)	303	0.59	(0.52-0.66)	13	0.27	(0.14-0.46)	16	0.49	(0.28-0.79	12	0.76	(0.39-1.32)
Men															
1999	158	0.82	(0.69-0.97	128	0.79	(0.65-0.95)	15	0.99	(0.5-1.71)	12	1.24	(0.58-2.26	3	0.46	(0.06-1.44)
2000	129	0.61	(0.51-0.73	110	0.62	(0.51-0.75)	8	0.42	(0.18-0.84)	7	0.68	(0.27-1.43	4	0.76	(0.2-1.96)
2001	90	0.43	(0.34-0.53	81	0.47	(0.37-0.58)	7	0.35	(0.14-0.72)	2	0.16	(0.02-0.58	0	0	(-)
2002	100	0.48	(0.38-0.58	87	0.48	(0.38-0.6)	4	0.22	(0.06-0.58)	9	1.04	(0.44-2.03	0	0	(-)
2003	139	0.67	(0.56-0.8)	115	0.67	(0.54-0.81)	7	0.48	(0.18-1.02)	13	1.1	(0.58-1.89	4	0.62	(0.16-1.6)
2004	113	0.51	(0.42-0.62	99	0.53	(0.43-0.65)	8	0.52	(0.21-1.04)	4	0.31	(0.08-0.78	2	0.32	(0.04-1.17)
2005	122	0.56	(0.46-0.67	101	0.55	(0.44-0.67)	8	0.48	(0.2-0.96)	8	0.63	(0.27-1.24	5	1.06	(0.34-2.49)
2006	118	0.54	(0.44-0.65	99	0.53	(0.43-0.65)	9	0.44	(0.2-0.84)	7	0.84	(0.3-1.79)	3	0.49	(0.08-1.47)
2007	147	0.67	(0.56-0.79	124	0.66	(0.55-0.8)	18	1.18	(0.63-1.96)	4	0.31	(0.08-0.79	1	0.2	(0.01-1.11)
2008	170	0.76	(0.65-0.89	153	0.82	(0.69-0.97)	10	0.5	(0.23-0.92)	3	0.23	(0.05-0.67	4	0.7	(0.18-1.82)
2009	142	0.6	(0.5-0.71)	126	0.64	(0.53-0.76)	12	0.6	(0.31-1.05)	2	0.16	(0.02-0.59	2	0.28	(0.03-1.02)
2010	163	0.69	(0.58-0.8)	145	0.73	(0.61-0.86)	11	0.54	(0.27-0.98)	4	0.31	(0.09-0.81	3	0.45	(0.09-1.31)
2011	166	0.71	(0.6-0.82)	144	0.72	(0.61-0.85)	12	0.6	(0.31-1.05)	4	0.37	(0.08-0.98	6	1.3	(0.43-2.9)
2012	158	0.62	(0.53-0.73	140	0.66	(0.55-0.78)	8	0.4	(0.17-0.79)	3	0.23	(0.05-0.67	7	1.14	(0.44-2.37)
2013	245	0.96	(0.84-1.09	228	1.07	(0.93-1.22)	9	0.41	(0.18-0.78)	5	0.38	(0.12-0.88	3	0.46	(0.09-1.36)
2014	244	0.94	(0.83-1.07	220	1.01	(0.88-1.15)	7	0.42	(0.15-0.88)	14	1.02	(0.55-1.73	3	0.49	(0.1-1.43)
2015	192	0.72	(0.62-0.83	176	0.78	(0.67-0.91)	6	0.35	(0.11-0.79)	6	0.47	(0.16-1.03	4	0.61	(0.16-1.56)
2016	198	0.73	(0.63-0.84	176	0.77	(0.66-0.89)	8	0.48	(0.19-0.97)	9	0.65	(0.29-1.24	5	0.85	(0.24-2.05)
2017	191	0.68	(0.58-0.78	164	0.69	(0.59-0.8)	9	0.48	(0.21-0.94)	10	0.66	(0.31-1.21	8	1.05	(0.44-2.1)
Women															
1999	90	0.31	(0.25-0.38	78	0.32	(0.26-0.41)	6	0.24	(0.09-0.51)	3	0.21	(0.04-0.61	3	0.43	(0.09-1.26)
2000	80	0.27	(0.22-0.34	65	0.27	(0.21-0.34)	6	0.25	(0.09-0.54)	5	0.33	(0.11-0.76	4	0.55	(0.15-1.42)
2001	81	0.28	(0.22-0.35	70	0.29	(0.23-0.37)	5	0.2	(0.07-0.48)	6	0.36	(0.13-0.79	0	0	(-)
2002	80	0.27	(0.21-0.34	75	0.31	(0.24-0.38)	1	0.04	(0-0.24)	4	0.24	(0.06-0.61	0	0	(-)
2003	80	0.27	(0.21-0.34	71	0.29	(0.22-0.36)	5	0.21	(0.07-0.48)	2	0.11	(0.01-0.41	2	0.29	(0.03-1.03)
2004	77	0.26	(0.21-0.33	67	0.28	(0.21-0.35)	7	0.26	(0.1-0.53)	2	0.14	(0.02-0.52	1	0.09	(0-0.53)
2005	90	0.31	(0.25-0.38	73	0.3	(0.23-0.38)	5	0.19	(0.06-0.45)	8	0.51	(0.22-1.01	4	0.55	(0.15-1.42)
2006	73	0.25	(0.19-0.31	63	0.26	(0.2-0.33)	6	0.24	(0.09-0.52)	2	0.1	(0.01-0.37	2	0.24	(0.03-0.87)
2007	92	0.3	(0.24-0.37	78	0.31	(0.25-0.39)	10	0.36	(0.17-0.66)	2	0.12	(0.01-0.45	2	0.26	(0.03-0.93)
2008	97	0.32	(0.26-0.39	85	0.34	(0.27-0.42)	6	0.22	(0.08-0.47)	6	0.34	(0.12-0.75	0	0	(-)
2009	102	0.33	(0.26-0.4)	93	0.36	(0.29-0.44)	2	0.07	(0.01-0.26)	4	0.22	(0.06-0.58	3	0.34	(0.07-0.99)
2010	114	0.36	(0.3-0.44)	99	0.38	(0.3-0.46)	8	0.31	(0.13-0.61)	3	0.19	(0.04-0.57	4	0.49	(0.13-1.27)
2011	102	0.32	(0.26-0.38	92	0.34	(0.27-0.42)	1	0.03	(0-0.18)	5	0.28	(0.09-0.67	4	0.49	(0.13-1.26)
2012	132	0.4	(0.34-0.48	120	0.44	(0.36-0.53)	3	0.11	(0.02-0.33)	5	0.28	(0.09-0.65	4	0.49	(0.13-1.27)
2013	170	0.52	(0.44-0.6)	161	0.59	(0.5-0.69)	4	0.14	(0.04-0.37)	3	0.16	(0.03-0.48	2	0.23	(0.03-0.83)
2014	212	0.64	(0.56-0.74	190	0.69	(0.6-0.8)	10	0.36	(0.17-0.66)	7	0.39	(0.16-0.81	5	0.59	(0.19-1.38)
2015	144	0.42	(0.36-0.5)	132	0.46	(0.39-0.55)	4	0.15	(0.04-0.39)	7	0.36	(0.14-0.73	1	0.12	(0-0.65)
2016	154	0.45	(0.38-0.53	144	0.51	(0.43-0.6)	2	0.07	(0.01-0.25)	4	0.19	(0.05-0.49	4	0.46	(0.12-1.19)
2017	153	0.45	(0.38-0.52	139	0.49	(0.41-0.58)	4	0.14	(0.04-0.36)	6	0.35	(0.13-0.77	4	0.41	(0.11-1.05)

ASR, Age-standardised Rate; ASMR, Age-standardised Mortality Rate; CI, Confidence Interval

Table S6 Primary liver cancer one-year age-standardised net-survival by nation, sex, subtype and 5-year cohort

		UK				England				Scotland				Wales				Northern Ireland			
	Cohort	N	Net Survival	95% CI	suppress	N	Net Survival	95% CI	suppress	N	Net Survival	95% CI	suppress	N	Net Survival	95% CI	suppress	N	Net Survival	95% CI	suppress
PLC																					
Persons	1997-2001	11036	21.7	(20.9-22.5)		9001	21.4	(20.5-22.3)		1177	25.4	(22.8-28)		632	20.7	(17.5-24)		226	18.6	(13.8-24.1)	
	1998-2002	11576	22.5	(21.7-23.3)		9492	22.3	(21.5-23.2)		1201	25	(22.5-27.5)		653	21.9	(18.8-25.3)		230	17.5	(12.8-22.8)	
	1999-2003	12039	23.4	(22.6-24.2)		9879	23.2	(22.3-24.1)		1246	26.3	(23.9-28.9)		679	22.6	(19.5-25.9)		235	19	(14.2-24.4)	
	2000-2004	12522	24.1	(23.4-24.9)		10250	23.8	(23-24.7)		1340	27.8	(25.4-30.3)		696	24.4	(21.2-27.8)		236	19.3	(14.5-24.7)	
	2001-2005	13050	24.5	(23.7-25.2)		10674	24.2	(23.4-25.1)		1414	27.4	(25.1-29.9)		721	24.1	(20.9-27.3)		241	21.4	(16.4-26.9)	
	2002-2006	13905	25.6	(24.8-26.3)		11340	25.4	(24.6-26.3)		1509	28.4	(26-30.7)		758	24.4	(21.3-27.6)		298	20.9	(16.4-25.8)	
	2003-2007	14771	26.4	(25.7-27.1)		11986	26.3	(25.5-27.2)		1646	28.5	(26.3-30.8)		825	24.3	(21.3-27.3)		314	22.8	(18.2-27.7)	
	2004-2008	15828	27.6	(26.9-28.3)		12844	27.6	(26.8-28.3)		1727	30	(27.8-32.3)		916	25.0	(22.2-27.9)		341	23.9	(19.4-28.6)	
	2005-2009	17197	29.1	(28.4-29.8)		14054	29	(28.2-29.8)		1794	31.8	(29.6-34)		968	26.8	(24-29.7)		381	28.3	(23.8-33)	
	2006-2010	18525	30.8	(30.1-31.4)		15225	30.7	(29.9-31.4)		1889	33.1	(30.9-35.3)		1003	28.8	(26-31.7)		408	28.5	(24.2-33.1)	
	2007-2011	19731	32.1	(31.4-32.8)		16185	31.7	(31-32.5)		2018	34.7	(32.6-36.9)		1097	32.8	(29.9-35.6)		431	32.5	(28.1-37.1)	
	2008-2012	21132	33.8	(33.2-34.5)		17337	33.2	(32.5-33.9)		2164	37.8	(35.7-40)		1175	35.6	(32.8-38.4)		456	35.7	(31.2-40.2)	
	2009-2013	23006	35.5	(34.9-36.2)		18835	34.8	(34.1-35.5)		2413	40.3	(38.3-42.3)		1254	36.5	(33.8-39.2)		504	38.5	(34.1-42.9)	
	2010-2014	24670	37.4	(36.8-38.1)		20161	36.9	(36.2-37.5)		2644	41.3	(39.3-43.2)		1310	37.9	(35.2-40.6)		555	38.4	(34.2-42.6)	
	2011-2015	26343	39.1	(38.5-39.7)		21402	38.6	(37.9-39.3)		2873	43.7	(41.8-45.5)		1463	37.5	(35-40)		605	39.8	(35.7-43.7)	
	2012-2016	27984	39.8	(39.2-40.4)		22840	39.4	(38.7-40)		3025	44.1	(42.3-46)		1487	37.7	(35.2-40.3)		632	39.5	(35.6-43.4)	
	2013-2017	29029	40.6	(40-41.2)		23773	40.2	(39.6-40.9		3107	44.6	(42.8-46.4					ShortFU	656	39.9	(36-43.7)	
	Total Change*		18.9				18.8				19.2				17.0				21.3		
Males	1997-2001	6668	20.8	(19.8-21.8)		5475	20.4	(19.3-21.5)		710	25	(21.8-28.4)		362	18.3	(14.5-22.6)		121	19.2	(12.6-26.9)	
	1998-2002	7071	21.6	(20.6-22.5)		5838	21.4	(20.3-22.4)		727	24.5	(21.4-27.8)		377	21.1	(17.1-25.5)		129	13.6	(8.3-20.3)	
	1999-2003	7428	22.4	(21.4-23.4)		6135	22.3	(21.2-23.3)		759	24.5	(21.4-27.7)		397	22.2	(18.1-26.5)		137	16.3	(10.6-23.2)	
	2000-2004	7726	23.3	(22.4-24.3)		6354	23.2	(22.1-24.3)		823	25.1	(22.1-28.2)		402	23.7	(19.6-28.1)		147	16.3	(10.8-23)	
	2001-2005	8072	24.0	(23-24.9)		6619	23.9	(22.8-24.9)		884	25.6	(22.7-28.6)		411	24.2	(20.1-28.6)		158	17.5	(12-24)	
	2002-2006	8690	25.1	(24.2-26.1)		7094	25.1	(24.1-26.2)		960	27	(24.2-30)		443	24.1	(20.2-28.3)		193	18.2	(13-24.1)	
	2003-2007	9275	26.4	(25.5-27.3)		7520	26.4	(25.4-27.5)		1065	27.8	(25.1-30.6)		486	24.7	(20.9-28.7)		204	22.2	(16.7-28.3)	
	2004-2008	9984	28.0	(27.1-28.9)		8088	27.9	(26.9-28.9)		1135	30.3	(27.6-33.1)		539	25.4	(21.7-29.2)		222	23.8	(18.3-29.7)	
	2005-2009	10911	29.4	(28.5-30.2)		8866	29	(28-30)		1205	32.9	(30.2-35.6)		586	28.2	(24.5-32)		254	28.6	(23.1-34.4)	
	2006-2010	11784	31.3	(30.5-32.2)		9610	31.2	(30.2-32.1)		1286	33.6	(30.9-36.3)		619	29.8	(26.2-33.5)		269	29.5	(24.1-35.2)	
	2007-2011	12580	32.8	(32-33.6)		10222	32.4	(31.5-33.3)		1383	35.4	(32.8-38)		688	33.7	(30.1-37.4)		287	33.1	(27.6-38.7)	
	2008-2012	13545	34.6	(33.8-35.4)		10995	33.9	(33-34.8)		1473	39.4	(36.9-42)		756	35.9	(32.4-39.4)		321	35.6	(30.2-40.9)	
	2009-2013	14800	36.4	(35.6-37.2)		11978	35.7	(34.8-36.5)		1659	41.5	(39.1-44)		809	36.8	(33.4-40.3)		354	38.1	(32.9-43.3)	
	2010-2014	15998	38.7	(37.9-39.4)		12953	38.2	(37.3-39.1)		1807	41.7	(39.4-44)		859	38.4	(35.1-41.8)		379	39.1	(34-44.1)	
	2011-2015	17122	40.4	(39.6-41.2)		13782	39.9	(39-40.7)		1957	45.5	(43.2-47.7)		979	38.1	(34.9-41.2)		404	40.1	(35.1-44.9)	
	2012-2016	18299	41.0	(40.3-41.7)		14815	40.4	(39.6-41.3)		2057	46.2	(43.9-48.4)		1011	39.8	(36.7-42.9)		416	40.3	(35.4-45.1)	
	2013-2017	19027	41.9	(41.2-42.6)		15480	41.5	(40.7-42.3)		2122	46.4	(44.2-48.6)					ShortFU	420	40.8	(35.9-45.6)	
	Total Change*		21.1				21.1				21.4				21.5				21.6		
Females	1997-2001	4368	24.1	(22.8-25.4)		3526	23.9	(22.5-25.4)		467	26.5	(22.5-30.6)		270	24.2	(19.2-29.5)		105	16.8	(10.3-24.7)	
	1998-2002	4505	24.8	(23.5-26.1)		3654	24.9	(23.4-26.3)		474	26	(22-30.1)		276	23.2	(18.3-28.4)		101	21.8	(14.2-30.5)	
	1999-2003	4611	26.0	(24.7-27.3)		3744	25.7	(24.3-27.2)		487	30.1	(26-34.4)		282	23.7	(18.8-28.9)		98	24.2	(16.1-33.3)	
	2000-2004	4796	26.4	(25.1-27.7)		3896	25.8	(24.4-27.2)		517	32.3	(28.2-36.5)		294	26.1	(21.2-31.4)		89	26.7	(17.9-36.5)	
	2001-2005	4978	26.1	(24.9-27.4)		4055	25.6	(24.3-27)		530	31.2	(27.2-35.3)		310	25.3	(20.5-30.3)		83	32	(22.1-42.4)	
	2002-2006	5215	27.0	(25.7-28.2)		4246	26.6	(25.2-28)		549	31.9	(27.9-35.9)		315	25.6	(20.8-30.6)		105	28.4	(20-37.4)	
	2003-2007	5496	26.8	(25.6-28)		4466	26.7	(25.3-28)		581	30.6	(26.8-34.5)		339	24.3	(19.8-29.1)		110	19.2	(12.4-27.2)	
	2004-2008	5844	27.1	(26-28.3)		4756	27.1	(25.8-28.4)		592	29.8	(26-33.6)		377	25.3	(21-29.9)		119	19.2	(12.6-26.8)	
	2005-2009	6286	29.1	(27.9-30.2)		5188	29.2	(28-30.5)		589	30.2	(26.4-34)		382	25.8	(21.5-30.4)		127	27.3	(19.8-35.4)	
	2006-2010	6741	30.0	(28.9-31.1)		5615	30	(28.7-31.2)		603	33	(29.2-36.9)		384	27.5	(23-32.1)		139	27	(19.8-34.7)	
	2007-2011	7151	31.1	(30-32.2)		5963	30.7	(29.5-31.9)		635	33.7	(30-37.5)		409	31.5	(26.9-36.1)		144	32.4	(24.8-40.3)	
	2008-2012	7587	32.5	(31.5-33.6)		6342	32.1	(30.9-33.2)		691	34.8	(31.2-38.5)		419	35.2	(30.5-39.9)		135	37.8	(29.5-46.1)	
	2009-2013	8206	34.2	(33.2-35.3)		6857	33.6	(32.4-34.7)		754	38.3	(34.7-41.8)		445	36.3	(31.8-41)		150	40.2	(32.2-48.2)	
	2010-2014	8672	35.2	(34.1-36.2)		7208	34.4	(33.3-35.6)		837	40.4	(36.9-43.8)		451	36.2	(31.6-40.7)		176	37.7	(30.4-45.1)	
	2011-2015	9221	36.6	(35.6-37.6)		7620	36.2	(35.1-37.3)		916	40	(36.8-43.3)		484	36.0	(31.6-40.4)		201	40	(33.1-46.9)	
	2012-2016	9685	37.7	(36.7-38.7)		8025	37.6	(36.5-38.7)		968	40	(36.8-43.2)		476	33.7	(29.4-38.1)		216	38.4	(31.8-45)	
	2013-2017	10002	38.1	(37.1-39.1)		8293	37.9	(36.8-39)		985	40.8	(37.6-43.9)					ShortFU	236	38.7	(32.4-45.1)	
	Total Change*		14.0				14.0				14.3				9.5				21.9		

HCC		UK				England				Scotland				Wales				Northern Ireland			
	Cohort	N	Net Survival	CI	suppress	N	Net Survival	CI	suppress	N	Net Survival	CI	suppress	N	Net Survival	CI	suppress	N	Net Survival	CI	suppress
**Persons**	**1997-2001**	4757	23.7	(22.5-24.9)		3865	23.6	(22.3-25)		564	27.3	(23.6-31.1)		239	17.8	(13.2-23)		89	21.1	(13.2-30.3)	
	**1998-2002**	5157	24.3	(23.1-25.5)		4212	24.4	(23.1-25.7)		581	26.8	(23.2-30.5)		255	19.0	(14.4-24.2)		109	20.5	(13.4-28.7)	
	**1999-2003**	5515	25.3	(24.1-26.5)		4489	25.4	(24.1-26.7)		642	27.1	(23.6-30.7)		265	19.6	(15-24.7)		119	23.6	(16.2-31.7)	
	**2000-2004**	5812	26.3	(25.1-27.5)		4695	26.1	(24.9-27.4)		724	28.4	(25.1-31.8)		274	22.5	(17.6-27.7)		119	27.6	(19.7-36)	
	**2001-2005**	6215	26.7	(25.6-27.8)		5008	26.6	(25.3-27.8)		779	29.3	(26.1-32.6)		291	22.8	(18.1-27.9)		137	26	(18.8-33.7)	
	**2002-2006**	6672	27.9	(26.8-29)		5349	27.8	(26.6-29.1)		855	30.2	(27.1-33.4)		318	23.4	(18.8-28.3)		150	27.3	(20.3-34.8)	
	**2003-2007**	7210	29.2	(28.1-30.3)		5734	29.2	(28-30.4)		948	30.6	(27.6-33.6)		357	25.1	(20.7-29.8)		171	31.4	(24.4-38.6)	
	**2004-2008**	7729	30.9	(29.8-31.9)		6097	30.9	(29.7-32.1)		1020	33.3	(30.3-36.3)		419	26.4	(22.2-30.8)		193	29.8	(23.3-36.5)	
	**2005-2009**	8455	32.6	(31.5-33.6)		6679	32.5	(31.3-33.6)		1089	35.6	(32.7-38.5)		461	29.7	(25.5-34)		226	32.1	(26-38.3)	
	**2006-2010**	9200	34.8	(33.8-35.8)		7268	34.9	(33.8-36)		1189	36	(33.2-38.8)		500	31.9	(27.8-36.1)		243	32.8	(26.8-38.8)	
	**2007-2011**	9968	36.6	(35.6-37.6)		7829	36.3	(35.2-37.4)		1295	38.6	(35.9-41.3)		575	36.9	(32.9-41)		269	35.8	(30-41.7)	
	**2008-2012**	10726	38.8	(37.8-39.7)		8402	38.1	(37-39.2)		1408	42.8	(40.2-45.5)		627	39.2	(35.2-43.1)		289	38.6	(32.8-44.3)	
	**2009-2013**	11930	40.4	(39.5-41.4)		9332	39.6	(38.6-40.6)		1586	45.7	(43.2-48.2)		681	40.1	(36.3-43.9)		331	41.7	(36.3-47.1)	
	**2010-2014**	13053	42.7	(41.8-43.6)		10239	42.1	(41.2-43.1)		1736	46.4	(43.9-48.8)		709	41.3	(37.5-45)		369	43.3	(38.1-48.4)	
	**2011-2015**	14146	45.0	(44.1-45.8)		11065	44.3	(43.4-45.3)		1880	50.3	(47.9-52.6)		798	42.1	(38.6-45.6)		403	46	(40.9-50.9)	
	**2012-2016**	15283	45.8	(45-46.6)		12049	45.1	(44.2-46.1)		1998	51.9	(49.6-54.1)		808	42.0	(38.5-45.5)		428	44.6	(39.7-49.3)	
	**2013-2017**	16091	46.7	(45.9-47.5)		12781	46.2	(45.3-47.1)		2052	52.4	(50.1-54.6)					ShortFU	432	45.4	(40.5-50.1)	
	**Total Change***		23.0				22.6				25.1				24.2				24.3		
**Males**	**1997-2001**	3531	22.7	(21.3-24.1)		2873	22.4	(20.9-24)		427	26.8	(22.5-31.2)		170	16.8	(11.5-22.9)		61	17.8	(9.3-28.7)	
	**1998-2002**	3869	23.6	(22.3-25)		3173	23.5	(22-25)		442	27.1	(22.9-31.4)		178	19.2	(13.7-25.4)		76	17.6	(9.9-27.2)	
	**1999-2003**	4167	24.5	(23.2-25.9)		3409	24.4	(23-25.9)		485	27	(23-31.1)		189	20.4	(14.9-26.6)		84	18.7	(11.1-28)	
	**2000-2004**	4435	25.4	(24.1-26.7)		3602	25.3	(23.9-26.8)		550	26.7	(23-30.5)		198	22.2	(16.6-28.4)		85	21.9	(13.7-31.5)	
	**2001-2005**	4725	26.0	(24.7-27.3)		3828	25.9	(24.5-27.4)		594	28.2	(24.5-31.9)		204	23.2	(17.5-29.3)		99	20	(12.6-28.6)	
	**2002-2006**	5138	27.2	(26-28.5)		4152	27.3	(25.9-28.7)		646	28.7	(25.2-32.3)		229	24.3	(18.9-30.2)		111	23.7	(16.1-32.1)	
	**2003-2007**	5568	28.6	(27.4-29.8)		4448	28.7	(27.3-30.1)		727	29.4	(26-32.8)		264	25.9	(20.6-31.4)		129	28.2	(20.6-36.4)	
	**2004-2008**	6009	30.5	(29.3-31.7)		4765	30.5	(29.2-31.9)		790	32.5	(29.2-35.9)		308	27	(22.1-32.1)		146	28.8	(21.6-36.5)	
	**2005-2009**	6594	32.3	(31.2-33.5)		5219	32	(30.7-33.3)		853	35.4	(32.1-38.7)		348	31.9	(27-37)		174	32.3	(25.4-39.5)	
	**2006-2010**	7194	34.6	(33.5-35.7)		5688	34.6	(33.4-35.9)		938	35.5	(32.3-38.6)		383	33.4	(28.6-38.3)		185	35	(28.1-42.1)	
	**2007-2011**	7750	36.4	(35.3-37.5)		6069	36	(34.7-37.2)		1031	38.6	(35.5-41.6)		445	37.9	(33.3-42.5)		205	38.1	(31.3-44.8)	
	**2008-2012**	8353	38.5	(37.4-39.6)		6527	37.7	(36.5-38.9)		1113	43.1	(40.1-46.1)		489	39.6	(35.1-44)		224	40.8	(34.2-47.3)	
	**2009-2013**	9274	40.2	(39.1-41.2)		7215	39.3	(38.1-40.4)		1266	45.4	(42.6-48.2)		538	39.9	(35.7-44.1)		255	43.3	(37-49.5)	
	**2010-2014**	10178	42.4	(41.5-43.4)		7961	42	(40.9-43.1)		1380	45.1	(42.4-47.8)		559	40.4	(36.3-44.6)		278	44.9	(38.8-50.8)	
	**2011-2015**	11015	44.8	(43.8-45.7)		8598	44.2	(43.1-45.3)		1489	50	(47.4-52.6)		634	41.4	(37.4-45.3)		294	45.2	(39.3-51)	
	**2012-2016**	11929	45.4	(44.5-46.3)		9399	44.8	(43.7-45.8)		1571	51.3	(48.7-53.8)		648	42.3	(38.4-46.2)		311	43.1	(37.4-48.7)	
	**2013-2017**	12568	46.6	(45.7-47.5)		9983	46.1	(45.1-47.1)		1612	52.3	(49.8-54.8)					ShortFU	314	43.3	(37.6-48.8)	
	**Total Change***		23.9				23.7				25.5				25.5				25.5		
**Females**	**1997-2001**	1226	27.4	(24.9-30)		992	27.7	(24.9-30.6)		137	30	(22.4-37.9)		69	21.2	(12.4-31.7)		28	27.5	(12.6-44.9)	
	**1998-2002**	1288	26.9	(24.5-29.4)		1039	27.8	(25.1-30.6)		139	28.7	(21.3-36.5)		77	18.0	(10.3-27.5)		33	26.5	(12.9-42.4)	
	**1999-2003**	1348	28.6	(26.2-31.1)		1080	29.4	(26.6-32.2)		157	30.3	(23.2-37.8)		76	17.9	(10.2-27.5)		35	38.1	(22.3-54)	
	**2000-2004**	1377	29.9	(27.5-32.4)		1093	29.9	(27.2-32.7)		174	33.2	(26.2-40.4)		76	24.6	(15.5-34.9)		34	44.5	(27.4-60.5)	
	**2001-2005**	1490	29.8	(27.5-32.2)		1180	29.6	(27-32.3)		185	34.6	(27.7-41.6)		87	22.6	(14.4-32)		38	45.9	(29.5-61)	
	**2002-2006**	1534	30.6	(28.2-33)		1197	30.3	(27.7-33)		209	36.4	(29.7-43.1)		89	21.3	(13.4-30.5)		39	41.6	(25.9-56.8)	
	**2003-2007**	1642	31.9	(29.6-34.2)		1286	31.6	(29-34.2)		221	36	(29.6-42.5)		93	23.7	(15.5-32.8)		42	41.6	(26.4-56.3)	
	**2004-2008**	1720	32.5	(30.2-34.7)		1332	32.5	(29.9-35.1)		230	37.3	(31-43.7)		111	24.8	(17.1-33.2)		47	29.3	(17-42.9)	
	**2005-2009**	1861	33.8	(31.7-36)		1460	34.3	(31.9-36.8)		236	37.5	(31.2-43.8)		113	25.6	(17.9-34.1)		52	32.1	(19.8-45.1)	
	**2006-2010**	2006	35.7	(33.5-37.8)		1580	36	(33.6-38.4)		251	39.9	(33.7-46)		117	27.2	(19.4-35.7)		58	25.6	(15.1-37.5)	
	**2007-2011**	2218	37.4	(35.3-39.4)		1760	37.4	(35.1-39.7)		264	40.3	(34.3-46.3)		130	33.8	(25.7-42.2)		64	30.9	(20-42.6)	
	**2008-2012**	2373	39.7	(37.7-41.7)		1875	39.6	(37.3-41.8)		295	43	(37.2-48.7)		138	37.9	(29.7-46.2)		65	35	(23.5-46.7)	
	**2009-2013**	2656	41.8	(39.9-43.7)		2117	41	(38.9-43.2)		320	48.4	(42.6-53.9)		143	41.8	(33.5-50)		76	40.4	(29.1-51.4)	
	**2010-2014**	2875	43.9	(42-45.7)		2278	42.8	(40.7-44.9)		356	50.8	(45.4-56)		150	44.5	(36.2-52.5)		91	43.8	(33.3-54)	
	**2011-2015**	3131	45.6	(43.8-47.4)		2467	44.6	(42.6-46.6)		391	52.1	(46.9-57.1)		164	42.9	(35.1-50.5)		109	49.6	(39.7-58.8)	
	**2012-2016**	3354	47.3	(45.6-49.1)		2650	46.7	(44.7-48.6)		427	54.4	(49.4-59.2)		160	38.1	(30.4-45.7)		117	48.1	(38.6-57)	
	**2013-2017**	3523	47.3	(45.6-48.9)		2798	46.6	(44.7-48.5)		440	53.3	(48.4-58)					ShortFU	118	51.8	(42.2-60.6)	
	**Total Change***		19.9				18.9				23.3				16.9				24.3		

ICCA		UK				England				Scotland				Wales				Northern Ireland			
	Cohort	N	Net Survival	CI	suppress	N	Net Survival	CI	suppress	N	Net Survival	CI	suppress	N	Net Survival	CI	suppress	N	Net Survival	CI	suppress
**Persons**	**1997-2001**	4573	22.6	(21.4-23.9)		3711	22.2	(20.9-23.6)		488	25.3	(21.4-29.4)		264	26.6	(21.3-32.2)		110	14.6	(8.7-22.1)	
	**1998-2002**	4741	23.2	(22-24.4)		3866	23	(21.6-24.3)		498	24.3	(20.5-28.3)		277	27.9	(22.6-33.5)		100	18	(11.1-26.3)	
	**1999-2003**	4886	24.0	(22.8-25.3)		4004	23.6	(22.3-25)		491	26.6	(22.7-30.7)		301	26.7	(21.7-31.9)		90	16.7	(9.7-25.3)	
	**2000-2004**	5196	24.2	(23-25.4)		4278	23.6	(22.4-25)		506	28.4	(24.4-32.5)		327	27.5	(22.7-32.6)		85	12.8	(6.6-21)	
	**2001-2005**	5379	23.7	(22.6-24.9)		4433	23.4	(22.1-24.7)		528	25.6	(21.9-29.5)		341	26.8	(22.1-31.7)		77	16.4	(9-25.7)	
	**2002-2006**	5742	24.0	(22.9-25.2)		4716	23.7	(22.5-25)		549	25.9	(22.2-29.8)		361	26.9	(22.4-31.7)		116	16.3	(10.1-23.8)	
	**2003-2007**	6042	23.9	(22.8-25)		4945	23.8	(22.5-25)		589	26.9	(23.3-30.6)		402	24.5	(20.4-29)		106	13.1	(7.5-20.4)	
	**2004-2008**	6430	24.1	(23-25.2)		5284	23.9	(22.7-25.1)		595	27	(23.4-30.7)		438	25.1	(21.1-29.4)		113	19.3	(12.5-27.2)	
	**2005-2009**	6875	24.8	(23.8-25.9)		5705	24.6	(23.5-25.8)		600	28.4	(24.8-32.2)		452	24.4	(20.5-28.6)		118	25.9	(18.2-34.2)	
	**2006-2010**	7215	25.4	(24.4-26.4)		6061	25	(23.9-26.2)		591	30.8	(27-34.6)		451	26.7	(22.6-30.9)		112	24.3	(16.7-32.8)	
	**2007-2011**	7553	26.1	(25.1-27.1)		6383	25.7	(24.6-26.8)		610	30.1	(26.4-33.8)		461	26.9	(22.9-31.1)		99	27.2	(18.7-36.5)	
	**2008-2012**	7917	26.7	(25.8-27.8)		6687	26.1	(25-27.2)		652	30.2	(26.6-33.9)		473	30.6	(26.4-34.9)		105	30.3	(21.7-39.5)	
	**2009-2013**	8272	27.8	(26.8-28.8)		6938	27.2	(26.1-28.3)		731	31.2	(27.7-34.6)		492	30.3	(26.2-34.5)		111	31.3	(22.7-40.4)	
	**2010-2014**	8542	29.1	(28.1-30.1)		7109	28.5	(27.5-29.6)		804	32.7	(29.4-36.1)		506	31.5	(27.4-35.7)		123	26.5	(18.9-34.8)	
	**2011-2015**	9066	29.7	(28.8-30.7)		7464	29.6	(28.6-30.7)		900	31.2	(28.1-34.3)		555	29.4	(25.6-33.4)		147	25.5	(18.6-33)	
	**2012-2016**	9444	30.1	(29.2-31.1)		7783	30.3	(29.3-31.4)		936	28.4	(25.4-31.4)		568	30.9	(27.1-34.9)		157	27.5	(20.6-34.9)	
	**2013-2017**	9795	30.5	(29.6-31.4)		8109	30.7	(29.7-31.7)		958	28.5	(25.6-31.5)					ShortFU	173	28.3	(21.6-35.3)	
	**Total Change***		7.9				8.5				3.2				4.3				13.7		
**Males**	**1997-2001**	2138	22.2	(20.4-24)		1764	21.7	(19.7-23.7)		213	25.8	(20-32)		118	20.7	(13.8-28.7)					Insuff
	**1998-2002**	2203	21.9	(20.1-23.7)		1822	21.9	(20-23.9)		216	22.3	(16.9-28.3)		126	26.5	(19-34.8)					Insuff
	**1999-2003**	2295	22.9	(21.1-24.7)		1910	23.1	(21.1-25)		216	21.5	(16.2-27.4)		133	25.8	(18.5-33.8)					Insuff
	**2000-2004**	2415	23.2	(21.5-24.9)		2014	23.2	(21.3-25.1)		218	22.9	(17.4-28.9)		142	27.4	(20.1-35.1)					Insuff
	**2001-2005**	2525	23.2	(21.5-24.9)		2102	23.2	(21.3-25.1)		231	21.8	(16.6-27.5)		151	27.7	(20.7-35.3)					Insuff
	**2002-2006**	2712	23.5	(21.9-25.2)		2231	23.5	(21.7-25.3)		258	24.2	(19-29.7)		161	25.7	(19.1-32.9)		62	11.4	(5-20.9)	
	**2003-2007**	2838	24.1	(22.5-25.7)		2324	24	(22.3-25.9)		277	26.4	(21.2-31.9)		182	24.7	(18.5-31.3)		55	14.8	(6.9-25.7)	
	**2004-2008**	3020	24.5	(22.9-26)		2485	24.2	(22.5-25.9)		278	29.6	(24.2-35.2)		201	24.0	(18.2-30.3)		56	21.7	(11.9-33.5)	
	**2005-2009**	3247	24.7	(23.2-26.3)		2688	24.2	(22.6-25.9)		285	32.5	(27-38.2)		215	22.4	(17-28.3)		59	25.8	(15.3-37.7)	
	**2006-2010**	3385	25.8	(24.3-27.3)		2834	25.4	(23.7-27)		278	34.5	(28.8-40.3)		215	25.7	(19.9-31.9)		58	22.5	(12.6-34.2)	
	**2007-2011**	3552	26.5	(25.1-28)		3008	26.3	(24.7-27.9)		274	31.9	(26.3-37.7)		219	24.8	(19.2-30.8)		51	25.6	(14.4-38.4)	
	**2008-2012**	3750	27.2	(25.8-28.7)		3171	26.6	(25-28.2)		289	33.1	(27.6-38.7)		227	28.5	(22.6-34.7)		63	28.4	(17.7-40.1)	
	**2009-2013**	3903	28.2	(26.8-29.7)		3285	27.8	(26.2-29.3)		327	32.5	(27.4-37.8)		226	29.1	(23.2-35.3)		65	28.8	(18.2-40.5)	
	**2010-2014**	4053	30.2	(28.7-31.6)		3381	29.8	(28.2-31.4)		364	33.1	(28.2-38.1)		241	33.2	(27.2-39.3)		67	27.3	(17.1-38.7)	
	**2011-2015**	4320	30.1	(28.7-31.5)		3558	29.8	(28.3-31.4)		409	32.9	(28.2-37.6)		277	30.7	(25.2-36.3)		76	27	(17.4-37.6)	
	**2012-2016**	4506	30.2	(28.8-31.6)		3715	29.9	(28.4-31.5)		430	30.4	(26-35)		287	35.7	(30.1-41.4)		74	33.4	(22.8-44.6)	
	**2013-2017**	4695	30.2	(28.8-31.5)		3897	30.3	(28.8-31.8)		450	27.7	(23.5-32)					ShortFU	74	35.3	(24.4-46.5)	
	**Total Change***		8.0				8.6				1.9				15.0				NC		
**Females**	**1997-2001**	2435	23.7	(22-25.5)		1947	23.7	(21.7-25.6)		275	24.5	(19.5-29.9)		146	29.8	(22.5-37.6)		67	12.3	(5.7-21.6)	
	**1998-2002**	2538	24.8	(23.1-26.6)		2044	24.5	(22.6-26.4)		282	26.5	(21.3-31.9)		151	29.6	(22.4-37.2)		61	29.4	(18.3-41.4)	
	**1999-2003**	2591	25.6	(23.9-27.4)		2094	24.7	(22.8-26.6)		275	31.5	(25.9-37.2)		168	27.9	(21.2-35)		54	28.8	(17.2-41.6)	
	**2000-2004**	2781	25.4	(23.8-27.1)		2264	24.3	(22.5-26.1)		288	33.8	(28.2-39.5)		185	27.9	(21.5-34.7)		44	28.6	(16-42.8)	
	**2001-2005**	2854	24.6	(23-26.2)		2331	23.8	(22.1-25.6)		297	30.3	(25-35.8)		190	28.0	(21.7-34.6)		36	30.8	(16.4-46.5)	
	**2002-2006**	3030	24.8	(23.2-26.4)		2485	24.1	(22.4-25.9)		291	28.9	(23.7-34.4)		200	30.4	(24-37)		54	24.1	(13.5-36.4)	
	**2003-2007**	3204	23.7	(22.2-25.2)		2621	23.5	(21.9-25.2)		312	27.1	(22.1-32.3)		220	25.3	(19.7-31.4)		51	9	(3.1-19)	
	**2004-2008**	3410	23.8	(22.4-25.3)		2799	23.7	(22.1-25.3)		317	23.6	(19-28.6)		237	27.2	(21.6-33.1)		57	14.7	(6.9-25.3)	
	**2005-2009**	3628	25.0	(23.6-26.5)		3017	25.1	(23.5-26.7)		315	24	(19.3-28.9)		237	27.6	(22-33.6)		59	22.1	(12.5-33.6)	
	**2006-2010**	3830	25.0	(23.6-26.4)		3227	24.7	(23.2-26.2)		313	27.1	(22.2-32.3)		236	27.5	(21.9-33.5)		54	23.6	(13.2-35.8)	
	**2007-2011**	4001	25.6	(24.2-27)		3375	25.1	(23.6-26.6)		336	28.6	(23.8-33.7)		242	29.2	(23.5-35.2)		48	33.2	(20.2-46.9)	
	**2008-2012**	4167	26.3	(25-27.7)		3516	25.6	(24.2-27.1)		363	27.3	(22.7-32.1)		246	32.4	(26.5-38.5)		42	39.6	(24.7-54.4)	
	**2009-2013**	4369	27.4	(26.1-28.8)		3653	26.7	(25.3-28.2)		404	29.4	(25-34.1)		266	31.2	(25.6-37)		46	38.4	(24.2-52.6)	
	**2010-2014**	4489	28.1	(26.8-29.4)		3728	27.4	(26-28.9)		440	32.5	(28-37)		265	29.3	(23.8-35)		56	30.3	(18.6-42.9)	
	**2011-2015**	4746	29.5	(28.2-30.9)		3906	29.6	(28.1-31.1)		491	29.5	(25.4-33.7)		278	28.7	(23.3-34.2)		71	25.8	(16.1-36.7)	
	**2012-2016**	4938	30.3	(29-31.6)		4068	31	(29.5-32.4)		506	26.7	(22.8-30.7)		281	28.1	(22.9-33.7)		83	26.2	(17.1-36.2)	
	**2013-2017**	5100	31.1	(29.8-32.4)		4212	31.5	(30-32.9)		508	30	(26-34.1)					ShortFU	99	22.5	(14.7-31.3)	
	**Total Change***		7.4				7.8				5.5				-1.7				10.2		

Other		UK				England				Scotland				Wales				Northern Ireland			
	Cohort	N	Net Survival	CI	suppress	N	Net Survival	CI	suppress	N	Net Survival	CI	suppress	N	Net Survival	CI	suppress	N	Net Survival	CI	suppress
**Persons**	**1997-2001**	1648	13.2	(11.6-14.9)		1397	12.9	(11.2-14.8)		108	15.5	(9.3-23.2)		117	15.9	(9.8-23.2)		26	13.2	(3.6-29.2)	
	**1998-2002**	1610	14.4	(12.7-16.2)		1379	14.3	(12.4-16.2)		102	18.2	(11.3-26.5)		109	15.3	(9.2-22.9)		20	2.5	(0-17.7)	
	**1999-2003**	1571	15.6	(13.8-17.5)		1345	15.4	(13.4-17.4)		96	18.6	(11.4-27.3)		105	19.2	(12.2-27.5)		25	4.8	(0.4-18.4)	
	**2000-2004**	1442	16.8	(14.9-18.8)		1230	16.5	(14.5-18.7)		93	18.9	(11.6-27.8)		87	21.2	(13.2-30.5)		32	8.1	(1.8-20.9)	
	**2001-2005**	1386	18.5	(16.5-20.7)		1190	18.6	(16.4-20.9)		91	15.8	(9-24.3)		78	20.2	(12-30)		27	18.9	(6.8-35.7)	
	**2002-2006**	1414	21.0	(18.9-23.3)		1222	21.7	(19.4-24.1)		91	16.3	(9.4-24.9)		69	17.7	(9.6-27.9)					Insuff
	**2003-2007**	1443	22.6	(20.5-24.9)		1249	23.5	(21.2-26)		101	16.7	(10-24.8)		56	16.1	(7.8-27.2)					Insuff
	**2004-2008**	1591	24.6	(22.4-26.8)		1403	25.8	(23.5-28.2)		104	13.6	(7.7-21.2)		49	9.4	(3.2-19.7)					Insuff
	**2005-2009**	1793	28.3	(26.2-30.5)		1610	29.5	(27.2-31.8)		99	12.5	(6.8-20)		47	16.3	(7.3-28.6)					Insuff
	**2006-2010**	2032	30.4	(28.4-32.5)		1829	31.6	(29.4-33.8)		103	14.3	(8.3-22)		47	27.1	(15.1-40.7)		53	18.6	(9.4-30.3)	
	**2007-2011**	2141	31.6	(29.6-33.6)		1921	32.4	(30.3-34.6)		103	15.9	(9.5-23.8)		54	36.4	(23.6-49.5)		63	25.4	(15.3-36.8)	
	**2008-2012**	2420	33.4	(31.4-35.3)		2197	34.1	(32.1-36.1)		94	15.7	(9.1-24)		67	36.7	(25.1-48.5)		62	29.4	(18.5-41.3)	
	**2009-2013**	2736	35.9	(34-37.7)		2515	36.4	(34.4-38.3)		87	16.2	(9.2-25)					Insuff	62	30.9	(19.7-42.9)	
	**2010-2014**	3006	36.0	(34.2-37.8)		2763	36.5	(34.6-38.3)		94	20.4	(12.8-29.3)		86	43.7	(32.8-54.2)		63	30.9	(19.8-42.9)	
	**2011-2015**	3054	37.1	(35.3-38.9)		2820	37.3	(35.4-39.1)		83	25.6	(16.6-35.8)		97	42.7	(32.4-52.7)		54	41.6	(28.1-54.7)	
	**2012-2016**	3174	37.4	(35.6-39.1)		2949	37.6	(35.8-39.4)		77	18.7	(10.7-28.5)		102	41.3	(31.4-51.1)		46	41.1	(26.6-55.3)	
	**2013-2017**	3065	37.9	(36.1-39.6)		2831	38.2	(36.3-40)					Insuff				ShortFU	50	27.5	(15.8-40.7)	
	**Total Change***		24.7				25.3				NC				25.4				14.3		
**Males**	**1997-2001**	976	11.2	(9.2-13.3)		825	10.7	(8.7-13)		63	12.1	(5.4-21.7)		71	13.2	(6.5-22.4)					2yr>1yr
	**1998-2002**	970	12.3	(10.3-14.5)		826	11.8	(9.7-14.2)		61	15.7	(7.7-26.2)		69	15.1	(7.7-24.8)		14	3.1	(0-23.1)	
	**1999-2003**	936	12.6	(10.5-14.9)		794	11.9	(9.7-14.3)		53	15	(6.9-26.3)		72	21.6	(12.8-32)		17	6.5	(0.5-24.8)	
	**2000-2004**	841	14.1	(11.8-16.6)		714	13.4	(10.9-16.1)		49	16.1	(7.3-28.1)		57	22.5	(12.5-34.4)					2yr>1yr
	**2001-2005**	787	15.6	(13.1-18.4)		667	15.4	(12.7-18.3)		53	13.5	(5.9-24.5)		49	19.3	(9.5-31.7)		18	15.1	(3.3-35.4)	
	**2002-2006**	799	17.4	(14.8-20.2)		683	18	(15.1-21)		50	14.1	(6.1-25.6)		46	15.1	(6.5-27.4)					Insuff
	**2003-2007**	828	19.8	(17.1-22.7)		717	20.6	(17.7-23.8)					Insuff	34	15.8	(5.9-30.3)					Insuff
	**2004-2008**	915	22.4	(19.6-25.2)		808	23.7	(20.7-26.7)					Insuff	25	8.6	(1.5-23.9)					Insuff
	**2005-2009**	1030	24.4	(21.7-27.1)		926	25.6	(22.8-28.5)					Insuff	20	15.0	(3.5-34.4)					Insuff
	**2006-2010**	1166	26.8	(24.2-29.4)		1053	28.2	(25.4-31)					Insuff				Insuff	26	9	(1.8-23.8)	
	**2007-2011**	1242	28.0	(25.5-30.6)		1117	29.4	(26.7-32.1)		73	13.4	(6.7-22.5)					Insuff	31	9.8	(2.5-23.5)	
	**2008-2012**	1405	30.7	(28.2-33.2)		1270	31.9	(29.3-34.6)		66	13.9	(6.8-23.6)					Insuff	34	17.2	(6.8-31.9)	
	**2009-2013**	1586	34.3	(31.9-36.7)		1450	35.3	(32.8-37.8)		62	16.2	(8.2-26.7)					Insuff	34	19.7	(8.3-34.7)	
	**2010-2014**	1732	36.0	(33.7-38.3)		1584	36.8	(34.4-39.3)		60	19	(10.1-30.2)		54	35.2	(22.6-48.3)		34	25.4	(12.2-41.1)	
	**2011-2015**	1742	37.3	(35-39.6)		1595	37.9	(35.5-40.4)		55	18.2	(9.2-29.8)		59	32.1	(20.4-44.5)					Insuff
	**2012-2016**	1816	38.0	(35.7-40.3)		1667	38.4	(36-40.8)					Insuff	70	35.9	(24.6-47.4)					Insuff
	**2013-2017**	1721	38.4	(36-40.8)		1570	38.9	(36.4-41.4)					Insuff				ShortFU				Insuff
	**Total Change***		27.2				28.2				NC				22.7				NC		
**Females**	**1997-2001**	672	17.0	(14.2-20)		572	17.1	(14-20.3)		45	17.8	(8.2-30.6)		46	20.7	(10.3-33.5)					Insuff
	**1998-2002**	640	18.3	(15.3-21.5)		553	19	(15.8-22.5)		41	17.9	(7.9-31.4)		40	12.8	(4.7-25.3)					Insuff
	**1999-2003**	635	20.8	(17.6-24.1)		551	21.2	(17.8-24.8)		43	21.7	(10.7-35.5)					2yr>1yr				Insuff
	**2000-2004**	601	20.9	(17.7-24.3)		516	21.2	(17.7-24.9)		44	19.5	(9.2-32.8)					Insuff				Insuff
	**2001-2005**	599	22.2	(18.9-25.7)		523	22.6	(19-26.3)		38	13.3	(4.8-26.5)					Insuff				Insuff
	**2002-2006**	615	25.2	(21.7-28.7)		539	26.1	(22.3-29.9)		41	16	(6.6-29.3)					Insuff				Insuff
	**2003-2007**	615	25.8	(22.3-29.4)		532	27.1	(23.3-31)		44	15.4	(6.5-28)					Insuff				Insuff
	**2004-2008**	676	27.6	(24.2-31.1)		595	28.9	(25.2-32.6)		42	18.8	(8.6-32.1)					Insuff				Insuff
	**2005-2009**	763	34.1	(30.6-37.6)		684	35	(31.4-38.7)		36	22.9	(10.7-38.1)					Insuff				Insuff
	**2006-2010**	866	36.1	(32.8-39.4)		776	37.1	(33.6-40.6)					2yr>1yr	26	35.0	(17.4-53.6)					Insuff
	**2007-2011**	899	37.0	(33.8-40.2)		804	37.2	(33.8-40.7)					Insuff	33	40.4	(23.5-57)					Insuff
	**2008-2012**	1015	37.5	(34.4-40.5)		927	37.5	(34.3-40.7)					Insuff	32	45.1	(27.2-61.8)		28	38.1	(20.4-55.8)	
	**2009-2013**	1150	37.9	(35-40.8)		1065	37.6	(34.7-40.6)					Insuff				Insuff	28	38.3	(20.5-56.2)	
	**2010-2014**	1274	35.6	(32.9-38.3)		1179	35.4	(32.6-38.2)		34	20.2	(8.6-35.5)					Insuff	29	33.8	(17.3-51.5)	
	**2011-2015**	1312	36.6	(33.9-39.3)		1225	36.1	(33.3-38.9)		28	24	(10.1-41.4)		38	64.3	(46.3-78.3)					Insuff
	**2012-2016**	1358	36.5	(33.8-39.1)		1282	36.5	(33.8-39.2)					Insuff	32	54.9	(35.6-71.2)					Insuff
	**2013-2017**	1344	37.2	(34.5-39.8)		1261	37.3	(34.5-40)					Insuff				ShortFU				Insuff
	**Total Change***		20.2				20.2				NC				34.2				NC		

*Between 1997-2001 and 2013-2017 (2012-2016 for Wales). Suppressed results: Insuff, no deaths or data in at least one age band; <10, less than 10 cases in total; SE>0.2, standard error greater than 0.2; 2yr>1yr, 2yr survival longer than 1 year survival; NC: Not Calculatable

Table S7 Primary liver cancer two-year age-standardised net-survival by nation, sex, subtype and 5-year cohort

		UK				England				Scotland				Wales				Northern Ireland			
	Cohort	N	Net Survival	CI	suppress	N	Net Survival	CI	suppress	N	Net Survival	CI	suppress	N	Net Survival	CI	suppress	N	Net Survival	CI	suppres
**PLC**																					
**Persons**	**1997-2001**	2300	12.8	(12.1-13.4)		1848	12.7	(12-13.4)		286	13.6	(11.6-15.8)		126	13.7	(11.1-16.6)		41	7	(4.1-11.1)	
	**1998-2002**	2495	13.2	(12.6-13.9)		2035	13.2	(12.5-13.9)		287	13.8	(11.8-15.9)		138	14.1	(11.4-17)		39	8.7	(5.4-13)	
	**1999-2003**	2702	13.9	(13.3-14.6)		2200	13.9	(13.2-14.6)		314	13.8	(11.8-15.9)		148	15.5	(12.8-18.5)		43	10.3	(6.7-14.9)	
	**2000-2004**	2906	14.1	(13.5-14.7)		2348	14	(13.3-14.7)		357	14.8	(12.8-16.8)		164	16	(13.3-19)		44	11.1	(7.3-15.8)	
	**2001-2005**	3071	14.5	(13.8-15.1)		2488	14.3	(13.6-15)		371	15.3	(13.4-17.3)		167	15.7	(13.1-18.6)		50	12.6	(8.6-17.4)	
	**2002-2006**	3421	15.3	(14.7-16)		2777	15.3	(14.7-16)		410	15.6	(13.7-17.6)		178	15.7	(13.1-18.5)		60	13.3	(9.6-17.6)	
	**2003-2007**	3754	16.0	(15.4-16.7)		3042	16	(15.3-16.7)		449	16.6	(14.7-18.5)		193	16	(13.5-18.7)		69	14.8	(11-19.2)	
	**2004-2008**	4210	17.1	(16.5-17.7)		3412	17.1	(16.4-17.8)		498	19.0	(17.1-21)		221	15.5	(13.2-18.1)		79	13.5	(10-17.5)	
	**2005-2009**	4829	18.5	(17.9-19.1)		3929	18.3	(17.7-19)		549	20.8	(18.9-22.9)		250	17.9	(15.5-20.5)		104	16.9	(13.2-21)	
	**2006-2010**	5495	19.7	(19.1-20.3)		4505	19.5	(18.8-20.1)		602	22.2	(20.3-24.2)		279	20	(17.5-22.7)		113	18.8	(15.1-22.9)	
	**2007-2011**	6117	20.8	(20.2-21.4)		4960	20.2	(19.6-20.9)		675	24.4	(22.4-26.4)		347	21.5	(19-24.1)		136	21.8	(17.9-26)	
	**2008-2012**	6902	22.2	(21.6-22.8)		5557	21.5	(20.9-22.2)		789	27.0	(25.1-29)		403	23.1	(20.7-25.7)		157	23.2	(19.3-27.4)	
	**2009-2013**	7897	23.4	(22.9-24)		6339	22.7	(22-23.3)		937	29.0	(27.1-30.9)		441	24.2	(21.7-26.7)		187	26.2	(22.3-30.3)	
	**2010-2014**	8920	25.1	(24.5-25.7)		7181	24.5	(23.8-25.1)		1051	29.4	(27.6-31.2)		479	25.3	(22.9-27.8)		206	27.1	(23.3-31.1)	
	**2011-2015**	9947	26.5	(25.9-27)		7978	25.9	(25.3-26.5)		1207	31.2	(29.4-33)		530	25.3	(23-27.7)		232	25.7	(22.2-29.5)	
	**2012-2016**	10753	27.2	(26.6-27.7)		8686	26.7	(26.1-27.3)		1286	31.4	(29.7-33.2)					ShortFU	241	26.1	(22.6-29.7)	
	**Total Change***		14.4				14.0				17.8				11.6				19.1		
**Males**	**1997-2001**	1329	12.5	(11.7-13.3)		1071	12.4	(11.5-13.3)		169	14.3	(11.7-17.2)		64	11	(7.9-14.7)		22	5.8	(2.4-11.4)	
	**1998-2002**	1459	13.0	(12.1-13.8)		1194	12.8	(12-13.8)		170	14.2	(11.6-17)		76	12.8	(9.5-16.6)		17	6.5	(2.9-12.1)	
	**1999-2003**	1594	13.6	(12.8-14.4)		1309	13.5	(12.7-14.5)		178	13.7	(11.2-16.4)		84	14.7	(11.3-18.6)		21	8.4	(4.3-14.3)	
	**2000-2004**	1730	14.0	(13.2-14.8)		1415	14	(13.1-14.9)		197	13.4	(11.1-16)		91	15.8	(12.3-19.8)		23	9.4	(5.2-15.2)	
	**2001-2005**	1857	14.4	(13.6-15.2)		1516	14.4	(13.5-15.3)		216	14.2	(11.9-16.8)		95	15.9	(12.4-19.8)		27	10	(5.8-15.7)	
	**2002-2006**	2099	15.3	(14.5-16.1)		1714	15.3	(14.5-16.2)		249	15.1	(12.8-17.6)		103	16.2	(12.7-20)		34	10.7	(6.7-15.8)	
	**2003-2007**	2358	16.3	(15.5-17.1)		1913	16.3	(15.4-17.1)		284	16.4	(14.1-18.8)		116	16.7	(13.4-20.4)		44	14.5	(9.9-19.9)	
	**2004-2008**	2690	17.6	(16.9-18.4)		2176	17.6	(16.7-18.4)		331	19.2	(16.8-21.6)		132	17.4	(14.2-20.9)		51	13	(8.8-18.1)	
	**2005-2009**	3086	18.9	(18.2-19.7)		2478	18.6	(17.8-19.5)		381	21.6	(19.2-24.1)		159	19.6	(16.4-23.1)		70	16.8	(12.3-21.9)	
	**2006-2010**	3559	20.5	(19.7-21.2)		2890	20.2	(19.4-21.1)		416	22.3	(19.9-24.7)		178	21.6	(18.3-25.1)		77	19	(14.4-24.2)	
	**2007-2011**	3981	21.5	(20.8-22.3)		3193	21	(20.1-21.8)		472	24.6	(22.3-27.1)		224	23.2	(19.9-26.5)		92	22.2	(17.4-27.4)	
	**2008-2012**	4528	23.0	(22.2-23.7)		3598	22.3	(21.4-23.1)		560	28	(25.6-30.4)		262	24.1	(21-27.4)		111	22.5	(17.9-27.5)	
	**2009-2013**	5203	24.3	(23.6-25)		4125	23.6	(22.8-24.4)		664	29.7	(27.4-32)		288	24.6	(21.5-27.7)		130	25.1	(20.6-30)	
	**2010-2014**	5975	26.3	(25.6-27)		4779	25.7	(24.9-26.5)		726	29.7	(27.5-32)		319	26.6	(23.5-29.7)		143	26.6	(22-31.4)	
	**2011-2015**	6681	27.7	(27-28.4)		5309	27.1	(26.3-27.9)		856	33.1	(30.9-35.3)		361	26.5	(23.7-29.5)		156	25.4	(21.1-30)	
	**2012-2016**	7246	28.4	(27.7-29.1)		5787	27.8	(27.1-28.6)		914	32.9	(30.8-35.1)					ShortFU	162	26.2	(21.8-30.7)	
	**Total Change***		15.9				15.4				18.6				15.5				20.4		
**Females**	**1997-2001**	1013	13.7	(12.7-14.8)		812	13.6	(12.4-14.8)		119	13.7	(10.6-17.1)		63	17.7	(13.3-22.7)		17	6.1	(2.5-12)	
	**1998-2002**	1076	14.1	(13-15.2)		875	14	(12.9-15.2)		118	13.9	(10.8-17.3)		62	16.1	(11.9-20.8)		21	10.7	(5.5-17.9)	
	**1999-2003**	1154	14.8	(13.8-15.9)		929	14.7	(13.6-15.9)		141	14.4	(11.4-17.9)		65	17.1	(12.8-21.9)		23	13.5	(7.5-21.5)	
	**2000-2004**	1221	14.7	(13.6-15.7)		970	14.2	(13.1-15.4)		160	17.2	(13.9-20.7)		74	16.5	(12.4-21.2)		23	15.3	(8.5-23.9)	
	**2001-2005**	1255	14.8	(13.8-15.8)		1003	14.4	(13.3-15.5)		158	17.5	(14.3-21.1)		76	15.4	(11.6-19.8)		26	19.7	(11.7-29.3)	
	**2002-2006**	1356	15.6	(14.6-16.7)		1089	15.5	(14.4-16.6)		168	16.7	(13.5-20.1)		78	14.7	(11-19)		29	19.7	(12.5-28.2)	
	**2003-2007**	1422	15.8	(14.8-16.8)		1149	15.7	(14.6-16.8)		171	17.2	(14.1-20.5)		80	14.6	(11-18.8)		20	12.4	(6.9-19.7)	
	**2004-2008**	1533	16.3	(15.3-17.3)		1245	16.3	(15.2-17.4)		170	18.9	(15.7-22.3)		92	12.9	(9.7-16.6)		22	11.4	(6.4-18.1)	
	**2005-2009**	1765	18.0	(17-19)		1466	18	(16.9-19.1)		172	19.8	(16.5-23.2)		95	15.8	(12.3-19.8)		34	16.6	(10.6-23.8)	
	**2006-2010**	1956	18.5	(17.6-19.5)		1626	18.1	(17.1-19.2)		192	22.9	(19.5-26.5)		102	17.8	(14-21.9)		36	18.5	(12.4-25.6)	
	**2007-2011**	2147	19.5	(18.6-20.5)		1773	18.9	(17.9-20)		207	24.5	(21.1-28.1)		124	18.9	(15.2-23)		45	22.1	(15.6-29.5)	
	**2008-2012**	2387	20.9	(20-21.9)		1967	20.3	(19.3-21.3)		232	25.2	(21.9-28.7)		142	21.8	(17.8-26)		49	26.5	(19.1-34.4)	
	**2009-2013**	2715	22.0	(21.1-22.9)		2228	21.1	(20.1-22.1)		278	27.8	(24.5-31.2)		156	24.4	(20.4-28.7)		58	29.6	(22.3-37.3)	
	**2010-2014**	2949	22.8	(21.9-23.7)		2401	22	(21-23)		326	28.4	(25.3-31.7)		157	23.4	(19.4-27.6)		64	28.9	(22.2-36.1)	
	**2011-2015**	3265	24.1	(23.2-25)		2667	23.6	(22.6-24.6)		353	27.7	(24.7-30.8)		168	23.5	(19.6-27.5)		78	27.1	(20.9-33.7)	
	**2012-2016**	3524	25.0	(24.1-25.9)		2916	24.7	(23.7-25.7)		374	28.4	(25.5-31.4)					ShortFU	80	25.8	(19.9-32)	
	**Total Change***		11.3				11.1				14.7				5.8				19.7		

HCC		UK				England				Scotland				Wales				Northern Ireland			
	Cohort	N	Net Survival	CI	suppress	N	Net Survival	CI	suppress	N	Net Survival	CI	suppress	N	Net Survival	CI	suppress	N	Net Survival	CI	suppres
**Persons**	**1997-2001**	1085	15.4	(14.3-16.5)		879	15.5	(14.3-16.7)		148	15.6	(12.6-18.9)		41	12.2	(8.3-16.9)		18	11.6	(5.7-19.8)	
	**1998-2002**	1206	15.8	(14.8-16.9)		990	16	(14.8-17.2)		149	15.9	(12.9-19.2)		47	12.9	(9-17.5)		21	12.6	(7-20.1)	
	**1999-2003**	1345	16.6	(15.6-17.7)		1099	16.8	(15.7-18)		167	15.5	(12.7-18.6)		50	14.6	(10.5-19.3)		27	16.6	(10.2-24.4)	
	**2000-2004**	1473	17.0	(16-18.1)		1183	17	(15.9-18.2)		198	16.4	(13.6-19.3)		59	16.5	(12.2-21.3)		32	18.5	(11.8-26.6)	
	**2001-2005**	1600	17.1	(16.2-18.1)		1284	17.2	(16.1-18.3)		219	17.1	(14.4-20)		64	16.3	(12.2-21)		34	17.3	(11.2-24.7)	
	**2002-2006**	1794	18.2	(17.2-19.2)		1436	18.3	(17.3-19.4)		249	17.1	(14.6-19.9)		72	16.8	(12.8-21.4)		40	19.9	(13.7-27.1)	
	**2003-2007**	2035	19.2	(18.2-20.1)		1618	19.3	(18.2-20.3)		279	18.6	(16.1-21.3)		87	17.2	(13.3-21.5)		52	22.8	(16.5-29.7)	
	**2004-2008**	2307	20.5	(19.6-21.5)		1820	20.6	(19.6-21.7)		327	21.9	(19.3-24.6)		107	17.3	(13.7-21.3)		56	18.2	(12.9-24.2)	
	**2005-2009**	2664	21.9	(21-22.8)		2097	21.8	(20.8-22.9)		374	24.1	(21.5-26.8)		133	20.4	(16.7-24.3)		70	19.1	(14.1-24.8)	
	**2006-2010**	3099	23.5	(22.6-24.4)		2456	23.3	(22.3-24.3)		413	25.5	(23-28.2)		154	23.5	(19.8-27.5)		77	22.2	(17-27.9)	
	**2007-2011**	3537	24.9	(24.1-25.8)		2754	24.3	(23.3-25.3)		483	28.7	(26.1-31.3)		206	26.4	(22.7-30.2)		94	23.7	(18.6-29.1)	
	**2008-2012**	4030	27.1	(26.2-28)		3105	26.2	(25.2-27.2)		583	32.6	(30-35.2)		238	29	(25.3-32.7)		108	25.7	(20.7-31.2)	
	**2009-2013**	4679	28.5	(27.7-29.4)		3585	27.4	(26.5-28.4)		700	35.0	(32.5-37.5)		264	29.9	(26.3-33.6)		134	28.5	(23.5-33.7)	
	**2010-2014**	5408	30.8	(30-31.7)		4187	30	(29-30.9)		778	35.6	(33.2-38)		284	31.4	(27.8-35)		155	31.7	(26.8-36.7)	
	**2011-2015**	6171	33.0	(32.2-33.8)		4759	32.2	(31.3-33.1)		913	38.9	(36.6-41.3)		326	32.1	(28.7-35.5)		180	30.9	(26.2-35.7)	
	**2012-2016**	6783	33.8	(33-34.6)		5277	33.1	(32.2-34)		1002	39.7	(37.4-42)					ShortFU	185	31.4	(26.8-36)	
	**Total Change***		18.4				17.6				24.1				19.9				19.8		
**Males**	**1997-2001**	770	14.8	(13.6-16.1)		620	14.8	(13.5-16.2)		109	16.5	(12.9-20.4)		27	10.2	(6.1-15.6)		10	8.5	(2.9-18.2)	
	**1998-2002**	878	15.4	(14.2-16.6)		716	15.4	(14.1-16.8)		115	16.8	(13.3-20.7)		33	11.8	(7.5-17.3)		13	9.6	(4.1-18.4)	
	**1999-2003**	982	16.0	(14.9-17.2)		801	16.1	(14.8-17.4)		125	16.1	(12.8-19.7)		37	13.8	(9.2-19.4)		15	12.4	(6.1-21.2)	
	**2000-2004**	1085	16.4	(15.3-17.5)		879	16.5	(15.2-17.8)		141	15.5	(12.4-18.8)		42	15.6	(10.8-21.4)		18	14.8	(7.9-24)	
	**2001-2005**	1183	16.7	(15.6-17.8)		956	16.8	(15.6-18.1)		161	16.4	(13.4-19.7)		46	16.2	(11.3-21.9)		19	13.9	(7.6-22.2)	
	**2002-2006**	1349	17.6	(16.5-18.7)		1093	17.8	(16.6-19.1)		178	16.2	(13.4-19.4)		54	17.5	(12.6-23)		25	17.1	(10.5-25.2)	
	**2003-2007**	1537	18.6	(17.5-19.7)		1232	18.7	(17.5-19.9)		205	17.7	(14.9-20.8)		66	18.2	(13.6-23.4)		35	21.7	(14.7-29.6)	
	**2004-2008**	1770	20.2	(19.1-21.3)		1405	20.1	(19-21.4)		247	21.4	(18.5-24.5)		80	19.2	(14.8-24)		41	18.1	(12.1-25.2)	
	**2005-2009**	2060	21.8	(20.7-22.8)		1614	21.5	(20.3-22.7)		291	23.9	(20.9-27)		108	22.9	(18.4-27.6)		54	20.1	(14.3-26.7)	
	**2006-2010**	2409	23.5	(22.5-24.5)		1905	23.2	(22-24.3)		321	24.8	(22-27.8)		124	25.2	(20.8-29.9)		63	24.6	(18.4-31.3)	
	**2007-2011**	2733	24.9	(23.9-25.9)		2114	24.1	(23-25.2)		384	28.3	(25.4-31.2)		163	27.7	(23.5-32.2)		76	26.1	(20-32.6)	
	**2008-2012**	3116	27.0	(26-28)		2384	26	(24.9-27.1)		463	32.3	(29.4-35.2)		187	29.2	(25.1-33.5)		89	27.1	(21.2-33.3)	
	**2009-2013**	3607	28.3	(27.3-29.3)		2743	27.3	(26.2-28.4)		555	33.9	(31.2-36.7)		208	28.9	(24.9-32.9)		107	29.5	(23.7-35.5)	
	**2010-2014**	4185	30.5	(29.6-31.4)		3239	29.8	(28.7-30.8)		601	34.2	(31.6-36.9)		219	30.2	(26.3-34.3)		121	32.1	(26.4-37.9)	
	**2011-2015**	4780	32.7	(31.8-33.6)		3684	32	(30.9-33)		718	38.6	(36-41.3)		255	30.8	(27.1-34.6)		129	30.1	(24.7-35.7)	
	**2012-2016**	5243	33.3	(32.4-34.2)		4076	32.7	(31.7-33.7)		778	38.5	(36-41.1)					ShortFU	130	30.1	(24.8-35.5)	
	**Total Change***		18.5				17.9				22.0				20.6				21.6		
**Females**	**1997-2001**	325	17.5	(15.3-19.7)		266	17.7	(15.3-20.2)		40	14.7	(9.3-21.4)		14	16.2	(8.5-26.1)					<10
	**1998-2002**	336	17.4	(15.3-19.6)		280	17.9	(15.6-20.4)		39	14.3	(9-20.9)		13	13.8	(7.1-22.7)					<10
	**1999-2003**	374	19.1	(16.9-21.3)		307	19.6	(17.2-22.1)		46	13.8	(8.8-20)		13	16.6	(9.2-26)		13	30.3	(15.7-46.8)	
	**2000-2004**	400	19.7	(17.6-21.9)		317	19.7	(17.3-22.2)		56	19.3	(13.6-25.8)		18	19.4	(11.3-29.3)		15	28.3	(14-44.9)	
	**2001-2005**	431	19.1	(17-21.2)		339	19	(16.7-21.4)		62	19.7	(14.2-26.1)		19	16.9	(9.7-25.7)		17	28.5	(14.8-44.1)	
	**2002-2006**	455	20.4	(18.4-22.6)		351	20.7	(18.4-23.1)		73	20.1	(14.8-26.1)		19	15.5	(8.8-24)		16	29.2	(15.5-44.7)	
	**2003-2007**	507	21.6	(19.5-23.7)		393	21.9	(19.6-24.3)		77	22.2	(16.8-28.1)		22	14.8	(8.4-23)		17	24	(12.1-38.5)	
	**2004-2008**	542	22	(20-24.1)		419	22.7	(20.4-25.1)		83	24.3	(18.8-30.3)		27	12.8	(7.3-20)		13	14.9	(6.4-26.8)	
	**2005-2009**	612	22.7	(20.7-24.7)		487	23.4	(21.2-25.7)		86	25.8	(20.2-31.7)		28	15.7	(9.6-23.3)		16	15.6	(7.2-27.1)	
	**2006-2010**	696	23.9	(22-25.9)		553	23.9	(21.8-26.1)		97	29.2	(23.5-35.1)		31	18.6	(11.9-26.4)					2yr>1yr
	**2007-2011**	806	25.4	(23.5-27.3)		641	25.1	(23-27.2)		104	31.6	(25.8-37.5)		43	22.2	(15.2-30)		19	15.7	(7.9-25.9)	
	**2008-2012**	917	27.6	(25.8-29.5)		723	26.9	(24.9-29)		123	34.0	(28.4-39.8)		51	28.5	(20.9-36.6)		22	20.1	(11.2-31)	
	**2009-2013**	1081	29.7	(27.9-31.5)		846	28.2	(26.2-30.2)		150	39.5	(33.9-45.2)		58	35.5	(27.3-43.8)		30	27	(17.3-37.7)	
	**2010-2014**	1228	32.2	(30.5-34)		951	30.8	(28.8-32.8)		176	40.1	(34.7-45.4)		65	36.3	(28.3-44.4)		39	32.6	(22.9-42.8)	
	**2011-2015**	1391	34	(32.3-35.7)		1073	32.9	(31-34.9)		198	41.1	(36-46.2)		68	36.5	(28.8-44.3)		53	33.3	(24.4-42.6)	
	**2012-2016**	1546	35.9	(34.2-37.6)		1205	34.9	(33-36.8)		225	44.4	(39.4-49.3)					ShortFU	55	33.4	(24.7-42.3)	
	**Total Change***		18.4				17.2				29.7				20.3				NC		

ICCA		UK				England				Scotland				Wales				Northern Ireland			
	Cohort	N	Net Survival	CI	suppress	N	Net Survival	CI	suppress	N	Net Survival	CI	suppress	N	Net Survival	CI	suppress	N	Net Survival	CI	suppres
**Persons**	**1997-2001**	993	11.0	(10.1-12)		791	10.7	(9.7-11.8)		118	11.9	(9.1-15.1)		67	18.7	(14-23.8)		15	2.4	(0.6-7)	
	**1998-2002**	1054	11.2	(10.3-12.2)		851	11.1	(10.1-12.2)		116	11.8	(9-15)		74	15	(11-19.7)					2yr>1yr
	**1999-2003**	1126	11.6	(10.7-12.6)		908	11.4	(10.4-12.5)		125	12.2	(9.3-15.4)		77	15.3	(11.3-19.8)					2yr>1yr
	**2000-2004**	1205	11.4	(10.5-12.4)		971	11.2	(10.2-12.2)		137	13.2	(10.3-16.4)		87	14.3	(10.7-18.6)					2yr>1yr
	**2001-2005**	1226	11.6	(10.7-12.5)		996	11.3	(10.4-12.3)		129	13.1	(10.2-16.3)		88	14.7	(11-18.9)		12	7.3	(2.8-14.8)	
	**2002-2006**	1327	11.8	(11-12.7)		1076	11.6	(10.7-12.6)		136	12.6	(9.8-15.7)		94	14.7	(11.2-18.8)		18	7.4	(3.5-13.4)	
	**2003-2007**	1386	12.0	(11.2-12.9)		1130	11.7	(10.8-12.7)		151	13.8	(11-16.9)		95	15.1	(11.7-19)		13	3.7	(1.1-8.8)	
	**2004-2008**	1491	12.3	(11.4-13.1)		1217	12	(11.1-12.9)		154	15.3	(12.4-18.5)		106	14	(10.8-17.6)		21	6.7	(3-12.6)	
	**2005-2009**	1642	13.0	(12.2-13.9)		1352	12.6	(11.7-13.5)		164	16.4	(13.4-19.7)		106	15.6	(12.3-19.3)		30	11.5	(6.4-18.3)	
	**2006-2010**	1763	13.1	(12.3-13.9)		1460	12.7	(11.8-13.5)		175	17.3	(14.2-20.6)		116	16.1	(12.8-19.9)		26	11	(5.9-17.9)	
	**2007-2011**	1896	13.5	(12.7-14.3)		1578	13.2	(12.3-14.1)		176	16.8	(13.8-20)		120	13.2	(10.2-16.7)		26	16.7	(9.9-25)	
	**2008-2012**	2039	13.8	(13-14.6)		1680	13.5	(12.6-14.3)		189	16.1	(13.3-19.2)		139	13.7	(10.7-17.1)		31	17.8	(11-26)	
	**2009-2013**	2213	14.1	(13.4-14.9)		1817	13.8	(12.9-14.6)		219	15.9	(13.2-18.7)		143	15	(11.9-18.5)		33	20.2	(13.1-28.5)	
	**2010-2014**	2393	14.8	(14-15.6)		1956	14.6	(13.7-15.5)		253	16.5	(13.9-19.3)		154	15.2	(12.1-18.6)		31	17.2	(10.9-24.8)	
	**2011-2015**	2596	15.3	(14.5-16.1)		2132	15.4	(14.5-16.2)		270	15.3	(13-17.9)		157	14.1	(11.3-17.3)		36	14.4	(9.1-20.9)	
	**2012-2016**	2739	15.8	(15-16.6)		2273	16.1	(15.3-17)		255	13.4	(11.2-15.8)					ShortFU	42	14.6	(9.4-20.8)	
	**Total Change***		4.8				5.4				1.5				-4.6				12.2		
**Males**	**1997-2001**	452	10.6	(9.2-12)		365	10.5	(9.1-12.1)		52	11.2	(7.2-16.2)		23	12.3	(6.9-19.4)					Insuff
	**1998-2002**	460	10.4	(9.1-11.8)		381	10.5	(9.1-12.1)		46	9.5	(5.9-14.2)		32	13.3	(7.8-20.3)					Insuff
	**1999-2003**	501	11.0	(9.7-12.3)		421	11.2	(9.8-12.8)		44	8.4	(5-12.9)		33	14.1	(8.6-21)					Insuff
	**2000-2004**	535	11.1	(9.9-12.5)		448	11.3	(9.9-12.8)		48	8.5	(5.1-12.9)		37	14.5	(9-21.1)					Insuff
	**2001-2005**	561	11.3	(10.1-12.7)		467	11.2	(9.9-12.7)		48	9.8	(6.3-14.3)		40	15.9	(10.3-22.6)					Insuff
	**2002-2006**	612	11.5	(10.3-12.8)		503	11.4	(10.1-12.9)		60	11.9	(8.1-16.4)		40	15.5	(10.2-22)					<10
	**2003-2007**	656	12.3	(11.1-13.6)		536	12.1	(10.8-13.6)		70	13.4	(9.6-18)		43	15.7	(10.5-21.8)					<10
	**2004-2008**	709	12.7	(11.5-13.9)		577	12.5	(11.2-13.9)		79	15.4	(11.2-20.2)		46	15.8	(10.9-21.6)		12	5	(1.2-13.3)	
	**2005-2009**	770	12.9	(11.7-14.1)		625	12.4	(11.2-13.8)		89	17.4	(13-22.3)		46	15.3	(10.7-20.8)		15	8.7	(3.2-18)	
	**2006-2010**	838	13.3	(12.1-14.5)		690	13.1	(11.8-14.4)		92	16.7	(12.4-21.6)		53	17.4	(12.5-23.1)		13	5.7	(1.5-14.1)	
	**2007-2011**	905	13.5	(12.4-14.7)		759	13.5	(12.3-14.8)		84	14.7	(10.7-19.5)		52	13	(8.8-18.1)		13	13.8	(5.9-25.2)	
	**2008-2012**	981	13.3	(12.2-14.5)		810	13.1	(11.9-14.4)		92	15.9	(11.8-20.5)		62	13.2	(9.1-18.3)		17	14.5	(7-24.8)	
	**2009-2013**	1060	13.9	(12.7-15)		877	13.7	(12.5-15)		102	15.5	(11.7-19.9)		63	13.8	(9.5-18.9)		18	15.3	(7.6-25.7)	
	**2010-2014**	1177	15.1	(14-16.3)		969	15.1	(13.8-16.4)		116	15.4	(11.7-19.5)		77	17.1	(12.5-22.4)		17	15	(7.5-25.2)	
	**2011-2015**	1250	15.1	(14-16.3)		1022	15.2	(13.9-16.4)		129	15.8	(12.3-19.7)		82	16.4	(12.1-21.2)		20	12.5	(6.1-21.4)	
	**2012-2016**	1310	15.9	(14.8-17.1)		1071	16	(14.8-17.3)		125	15.0	(11.7-18.8)					ShortFU	24	14.1	(7.1-23.5)	
	**Total Change***		5.3				5.5				3.8				4.1			NC			
**Females**	**1997-2001**	556	11.9	(10.6-13.2)		443	11.2	(9.8-12.7)		65	13.1	(9.3-17.6)		42	23.2	(16.5-30.6)					<10
	**1998-2002**	606	12.3	(11-13.6)		481	11.8	(10.4-13.3)		72	14.7	(10.7-19.3)		43	16.9	(11.3-23.5)					2yr>1yr
	**1999-2003**	639	12.5	(11.2-13.9)		498	11.8	(10.4-13.3)		83	15.8	(11.6-20.6)		45	16	(10.7-22.2)		15	16.8	(8.1-28.4)	
	**2000-2004**	681	11.8	(10.6-13.1)		530	11	(9.7-12.4)		93	17.6	(13.3-22.4)		50	13.5	(8.9-19)		12	17.5	(7.9-30.4)	
	**2001-2005**	675	12.0	(10.8-13.3)		535	11.4	(10.1-12.7)		86	16.9	(12.7-21.6)		51	11.9	(7.6-17.1)		11	20.8	(9.2-35.8)	
	**2002-2006**	723	12.2	(11-13.4)		577	11.7	(10.5-13.1)		81	14.3	(10.4-18.9)		59	13	(8.6-18.2)		12	16.2	(7.7-27.7)	
	**2003-2007**	731	11.7	(10.5-12.9)		594	11.3	(10.1-12.6)		81	14.1	(10.4-18.5)		54	13.1	(8.9-18.1)					<10
	**2004-2008**	783	11.8	(10.7-13)		640	11.4	(10.2-12.7)		72	14.8	(11-19.3)		62	11.9	(8.1-16.6)					<10
	**2005-2009**	876	13.2	(12.1-14.4)		730	12.8	(11.6-14)		73	15.5	(11.6-20)		63	16.3	(11.8-21.5)		13	12.9	(5.7-23.2)	
	**2006-2010**	926	12.9	(11.9-14.1)		769	12.2	(11-13.4)		82	17.8	(13.7-22.5)		63	15.7	(11.3-20.9)		12	15.3	(7-26.6)	
	**2007-2011**	989	13.5	(12.4-14.6)		817	12.8	(11.7-14)		92	18.6	(14.5-23.2)		68	15.3	(11-20.3)		15	25.9	(14.3-39.3)	
	**2008-2012**	1059	14.3	(13.2-15.4)		870	13.9	(12.7-15.1)		95	16.5	(12.7-20.7)		77	15.4	(11.1-20.4)		16	27.7	(15-42.3)	
	**2009-2013**	1156	14.5	(13.4-15.6)		943	13.9	(12.8-15.1)		114	16.0	(12.5-19.9)		80	16.7	(12.4-21.6)		17	29.2	(16.6-43.2)	
	**2010-2014**	1216	14.5	(13.5-15.6)		987	14.1	(13-15.3)		137	17.7	(14.1-21.5)		75	14.5	(10.5-19.2)		16	20.1	(10.6-32.1)	
	**2011-2015**	1351	15.6	(14.6-16.7)		1115	15.7	(14.6-16.9)		139	14.9	(11.8-18.4)		77	14.2	(10.3-18.7)		17	17.6	(9.6-27.7)	
	**2012-2016**	1440	15.9	(14.8-16.9)		1214	16.4	(15.2-17.6)		130	12.2	(9.4-15.3)					ShortFU	21	15.8	(8.7-24.8)	
	**Total Change***		4.0				5.2				-0.9				-9.0			NC			

Other		UK				England				Scotland				Wales				Northern Ireland			
	Cohort	N	Net Survival	CI	suppress	N	Net Survival	CI	suppress	N	Net Survival	CI	suppress	N	Net Survival	CI	suppress	N	Net Survival	CI	suppress
**Persons**	**1997-2001**	208	8.5	(7.1-9.9)		172	8.7	(7.2-10.3)		16	7.0	(3.1-13.1)					2yr>1yr				<10
	**1998-2002**	221	9.3	(7.9-10.9)		188	9.3	(7.8-11)		18	8.2	(3.7-14.8)					2yr>1yr				<10
	**1999-2003**	234	10.1	(8.7-11.8)		198	9.9	(8.3-11.6)					2yr>1yr	19	18.1	(11.1-26.5)					<10
	**2000-2004**	232	10.3	(8.8-12.1)		195	9.9	(8.3-11.8)		17	10.5	(5.2-18.1)					2yr>1yr				<10
	**2001-2005**	246	12.1	(10.4-13.9)		212	11.9	(10.1-13.9)		14	9.2	(4.2-16.6)		15	18.5	(10.5-28.4)					<10
	**2002-2006**	285	14.2	(12.3-16.1)		254	14.5	(12.5-16.6)		14	9.2	(4.2-16.6)		12	15.6	(7.9-25.8)					Insuff
	**2003-2007**	313	15.1	(13.2-17.1)		282	15.8	(13.8-18)		16	6.6	(2.7-12.9)					<10				Insuff
	**2004-2008**	376	17.3	(15.4-19.3)		348	18.4	(16.3-20.5)		14	5.3	(2-11.1)					<10				Insuff
	**2005-2009**	488	21.3	(19.3-23.3)		456	22.2	(20.2-24.4)		12	5.7	(2.2-11.8)					<10				Insuff
	**2006-2010**	594	23.3	(21.4-25.3)		556	24.4	(22.3-26.5)		14	4.1	(1.3-9.5)		12	22.6	(11.6-36.1)					<10
	**2007-2011**	651	23.9	(22.1-25.9)		600	24.6	(22.6-26.6)		16	8.4	(4-15)		19	30	(18.1-43.1)		15	21.1	(11.8-32.4)	
	**2008-2012**	778	24.3	(22.5-26.1)		722	24.7	(22.8-26.6)					2yr>1yr	24	30.3	(19.4-42.2)		18	21.6	(12.1-33)	
	**2009-2013**	944	25.7	(24-27.4)		881	26	(24.3-27.9)		13	10.9	(5.3-18.8)					Insuff	18	23	(13.2-34.6)	
	**2010-2014**	1041	25.0	(23.4-26.6)		969	25.2	(23.5-26.9)		18	12.7	(6.7-20.7)		36	34.5	(24.3-45.1)		19	22.4	(12.7-34)	
	**2011-2015**	1089	24.6	(23-26.2)		1012	24.6	(23-26.3)		20	20.8	(12.4-30.8)		40	32.6	(23.1-42.5)		22	15.6	(7.2-27.1)	
	**2012-2016**	1139	24.4	(22.9-26)		1066	24.5	(22.9-26.2)		14	12.7	(6.1-21.9)					ShortFU	18	10	(3.3-21.4)	
	**Total Change***		15.9				15.8				5.7				NC				NC		
**Males**	**1997-2001**	104	6.8	(5.3-8.6)		85	6.8	(5.2-8.8)					<10				<10				SE>0.2
	**1998-2002**	114	7.4	(5.8-9.3)		93	7.1	(5.4-9.1)					<10				<10				<10
	**1999-2003**	112	8.0	(6.3-9.9)		90	7.3	(5.6-9.4)					<10				2yr>1yr				<10
	**2000-2004**	113	8.6	(6.7-10.7)		91	7.9	(6-10.2)					<10				2yr>1yr				SE>0.2
	**2001-2005**	118	10.1	(8-12.4)		98	9.9	(7.7-12.5)					<10				<10				<10
	**2002-2006**	133	12.0	(9.8-14.5)		117	12.3	(9.9-15.1)					<10				<10				Insuff
	**2003-2007**	157	13.6	(11.2-16.1)		142	14.2	(11.7-17)					Insuff				<10				Insuff
	**2004-2008**	196	15.7	(13.3-18.2)		184	16.7	(14.1-19.5)					Insuff				<10				Insuff
	**2005-2009**	241	18.1	(15.7-20.6)		228	19.1	(16.5-21.8)					Insuff				<10				Insuff
	**2006-2010**	300	21.1	(18.7-23.6)		285	22.4	(19.8-25.1)					Insuff				Insuff				<10
	**2007-2011**	335	21.6	(19.2-24)		316	22.7	(20.2-25.4)					<10				Insuff				<10
	**2008-2012**	415	22.5	(20.2-24.8)		391	23.5	(21.1-26)					<10				Insuff				<10
	**2009-2013**	523	24.8	(22.5-27)		493	25.6	(23.3-28)					<10				Insuff				<10
	**2010-2014**	600	25.3	(23.1-27.5)		561	25.9	(23.6-28.2)		11	10.4	(4.1-20.2)		18	26.3	(15.1-39)					<10
	**2011-2015**	625	24.4	(22.3-26.5)		582	24.6	(22.4-26.9)					<10	18	23.4	(13.3-35.4)					Insuff
	**2012-2016**	663	24.1	(22.1-26.2)		615	24.3	(22.1-26.5)					Insuff				ShortFU				Insuff
	**Total Change***		17.3				17.5				NC				NC				NC		
**Females**	**1997-2001**	109	11.5	(9.2-14.2)		93	12.2	(9.6-15.2)					<10				<10				Insuff
	**1998-2002**	112	12.8	(10.3-15.7)		101	13.5	(10.7-16.6)					<10				<10				Insuff
	**1999-2003**	126	13.9	(11.3-16.9)		112	14.2	(11.3-17.4)					<10				<10				Insuff
	**2000-2004**	121	13.2	(10.5-16.2)		105	13.2	(10.3-16.4)					<10				Insuff				Insuff
	**2001-2005**	128	14.8	(12-17.9)		113	14.5	(11.6-17.8)					<10				Insuff				Insuff
	**2002-2006**	149	16.8	(13.8-20)		135	17.3	(14.1-20.7)					<10				Insuff				Insuff
	**2003-2007**	153	17.2	(14.3-20.5)		139	18.2	(14.9-21.7)					<10				Insuff				Insuff
	**2004-2008**	180	19.6	(16.6-22.8)		165	20.7	(17.4-24.2)					<10				Insuff				Insuff
	**2005-2009**	250	25.9	(22.7-29.2)		230	26.8	(23.4-30.3)					<10				Insuff				Insuff
	**2006-2010**	301	26.9	(23.9-30)		277	27.5	(24.3-30.8)					SE>0.2				<10				Insuff
	**2007-2011**	320	27.5	(24.5-30.6)		288	27.3	(24.1-30.6)					Insuff	13	33.6	(17.8-50.6)					Insuff
	**2008-2012**	366	26.9	(24.1-29.8)		335	26.5	(23.6-29.5)					Insuff	14	38.4	(21.2-56)		10	32.4	(15.9-50.5)	
	**2009-2013**	420	26.8	(24.1-29.5)		387	26.4	(23.6-29.2)					Insuff				Insuff	10	32.6	(16-50.8)	
	**2010-2014**	437	24.2	(21.8-26.7)		402	23.9	(21.4-26.5)					<10				Insuff				<10
	**2011-2015**	462	24.8	(22.4-27.3)		426	24.5	(22-27.1)					<10	23	41.1	(24.8-57.3)					Insuff
	**2012-2016**	475	25.0	(22.6-27.4)		449	25	(22.6-27.5)					Insuff				ShortFU				Insuff
	**Total Change***		13.5				12.8				NC				NC				NC		

*Between 1997-2001 and 2012-2016 (2011-2015 for Wales). Suppressed results: Insuff, no deaths or data in at least one age band; <10, less than 10 cases in total; SE>0.2, standard error greater than 0.2; 2yr>1yr, 2yr survival longer than 1 year survival; NC: Not Calculatable

Table S8 Primary liver cancer five-year age-standardised net-survival by nation, sex, subtype and 5-year cohort

		UK				England				Scotland				Wales				Northern Ireland			
	**Cohort**	**N**	**Net Survival**	**CI**	**suppress**	**N**	**Net Survival**	**CI**	**suppress**	**N**	**Net Survival**	**CI**	**suppress**	**N**	**Net Survival**	**CI**	**suppress**	**N**	**Net Survival**	**CI**	**suppres**
**PLC**																					
**Persons**	**1997-2001**	1300	7.2	(6.7-7.8)		1053	7	(6.4-7.6)		146	8.5	(6.8-10.5)		81	9.1	(6.8-11.8)		14	3.8	(1.6-7.5)	
	**1998-2002**	1413	7.4	(6.9-8)		1155	7.3	(6.7-7.9)		151	7.9	(6.2-9.8)		85	10	(7.6-12.8)		18	3.3	(1.4-6.7)	
	**1999-2003**	1544	7.9	(7.4-8.5)		1264	7.8	(7.2-8.4)		157	8.1	(6.5-10)		98	11.1	(8.6-13.9)		22	3.4	(1.4-7.1)	
	**2000-2004**	1631	8.1	(7.6-8.6)		1323	7.9	(7.3-8.5)		181	8.7	(7.1-10.6)		103	10.7	(8.3-13.5)		24	5.3	(2.6-9.6)	
	**2001-2005**	1746	8.1	(7.6-8.7)		1417	7.9	(7.4-8.5)		197	9.1	(7.4-10.9)		105	10	(7.7-12.6)		28	6.7	(3.6-11.3)	
	**2002-2006**	1974	8.6	(8.1-9.2)		1614	8.5	(8-9.1)		215	9.4	(7.8-11.2)		110	9.5	(7.3-12)		36	7.1	(4.2-11.2)	
	**2003-2007**	2194	9.2	(8.7-9.7)		1778	9	(8.4-9.6)		249	10.7	(9.1-12.5)		122	9.4	(7.3-11.8)		43	7.4	(4.5-11.4)	
	**2004-2008**	2515	9.6	(9.1-10.2)		2039	9.6	(9-10.1)		301	11.4	(9.8-13.2)		132	8.2	(6.4-10.4)		43	6.6	(4-10.1)	
	**2005-2009**	2957	10.7	(10.2-11.3)		2395	10.6	(10-11.2)		344	13.3	(11.6-15.2)		161	10.2	(8.2-12.5)		60	6.9	(4.4-10.2)	
	**2006-2010**	3396	11.8	(11.3-12.4)		2752	11.6	(11.1-12.2)		387	14.3	(12.6-16.2)		188	12.3	(10.1-14.7)		72	9.1	(6.3-12.5)	
	**2007-2011**	3811	12.5	(12-13)		3047	12.1	(11.5-12.7)		454	15.8	(14-17.7)		220	13.5	(11.3-15.9)		88	10.9	(7.9-14.5)	
	**2008-2012**	4366	13.3	(12.8-13.8)		3477	12.9	(12.4-13.5)		540	16.7	(14.9-18.5)		253	14	(11.9-16.3)		99	12.7	(9.6-16.4)	
	**2009-2013**	5018	14.3	(13.8-14.8)		3975	13.9	(13.3-14.4)		647	18.4	(16.6-20.2)					ShortFU	123	14.2	(11-17.9)	
	**Total Change***		7.1				6.9				9.9				4.9				10.4		
**Males**	**1997-2001**	760	7.1	(6.4-7.8)		620	6.7	(5.9-7.4)		91	10.3	(7.8-13.2)		36	8.8	(5.8-12.7)					<10
	**1998-2002**	838	7.6	(6.9-8.3)		687	7.2	(6.4-7.9)		93	9.7	(7.3-12.5)		44	10.9	(7.5-15)					<10
	**1999-2003**	924	8.0	(7.3-8.7)		762	7.6	(6.9-8.4)		94	9.3	(7-12)		53	12.5	(9-16.6)		10	3.6	(1-9)	
	**2000-2004**	990	8.2	(7.5-8.9)		815	7.9	(7.2-8.7)		100	8.6	(6.5-11)		58	11.4	(8.1-15.4)		12	6.9	(3-13.2)	
	**2001-2005**	1070	8.2	(7.5-8.9)		879	8	(7.3-8.8)		114	8.2	(6.2-10.5)		59	10.6	(7.5-14.4)		14	7	(3.1-13.3)	
	**2002-2006**	1223	8.8	(8.1-9.5)		1005	8.7	(8-9.4)		132	9.0	(7-11.2)		65	10.5	(7.5-14.2)		19	6.9	(3.4-12.2)	
	**2003-2007**	1393	9.3	(8.7-10)		1129	9.1	(8.4-9.9)		159	10.2	(8.2-12.4)		74	11.4	(8.4-15)		27	7	(3.6-12.1)	
	**2004-2008**	1626	10.0	(9.4-10.7)		1315	9.9	(9.2-10.7)		199	11.5	(9.5-13.8)		86	10	(7.3-13.2)		27	6.2	(3.2-10.7)	
	**2005-2009**	1908	11.1	(10.4-11.8)		1526	10.8	(10.1-11.5)		238	13.8	(11.6-16.2)		106	12.2	(9.4-15.5)		39	6.2	(3.3-10.3)	
	**2006-2010**	2230	12.4	(11.7-13.1)		1799	12.2	(11.5-13)		263	14.4	(12.3-16.8)		124	14.4	(11.4-17.8)		48	7.5	(4.4-11.6)	
	**2007-2011**	2505	13.0	(12.3-13.7)		1984	12.6	(11.8-13.3)		313	15.9	(13.7-18.3)		148	15.3	(12.4-18.6)		59	10.2	(6.7-14.7)	
	**2008-2012**	2881	13.7	(13.1-14.4)		2267	13.4	(12.7-14.1)		380	16.9	(14.7-19.2)		169	14.3	(11.6-17.3)		67	11.7	(8.1-16.2)	
	**2009-2013**	3330	14.8	(14.1-15.4)		2614	14.4	(13.7-15.2)		454	18.5	(16.4-20.8)					ShortFU	82	13.7	(9.9-18.2)	
	**Total Change***		7.7				7.7				8.2				5.5			NC			
**Females**	**1997-2001**	561	7.6	(6.8-8.5)		446	7.7	(6.8-8.7)		59	6.3	(4.2-9.1)		45	10	(6.5-14.2)					<10
	**1998-2002**	592	7.4	(6.6-8.2)		479	7.6	(6.7-8.6)		61	5.6	(3.6-8.2)		42	9	(5.8-13.1)		10	1.8	(0.3-6.6)	
	**1999-2003**	638	8.1	(7.2-9)		515	8.3	(7.4-9.3)		65	6.1	(4-8.8)		45	9.5	(6.2-13.6)		12	2	(0.3-7.5)	
	**2000-2004**	657	8.1	(7.3-9)		519	8	(7.1-8.9)		82	8.9	(6.4-12)		46	10	(6.7-14.2)		13	2.3	(0.3-8.5)	
	**2001-2005**	690	8.2	(7.4-9)		547	7.8	(6.9-8.7)		85	10.8	(8-14.1)		45	9.6	(6.4-13.5)		15	7.1	(2.5-15.3)	
	**2002-2006**	762	8.4	(7.6-9.3)		616	8.3	(7.4-9.2)		84	10.3	(7.6-13.4)		44	8.4	(5.5-12.1)		19	7.5	(3.1-14.7)	
	**2003-2007**	810	8.9	(8.1-9.8)		656	8.8	(7.9-9.7)		92	11.9	(9.1-15.2)		47	7.2	(4.6-10.6)		13	7.3	(2.9-14.7)	
	**2004-2008**	890	8.9	(8.1-9.7)		725	8.9	(8-9.8)		103	11.4	(8.7-14.5)		46	6.4	(4.1-9.4)		13	5.6	(2.2-11.4)	
	**2005-2009**	1060	10.2	(9.4-11)		876	10.2	(9.3-11.1)		108	12.6	(9.8-15.8)		57	7.9	(5.3-11.1)		20	7.7	(3.7-13.7)	
	**2006-2010**	1170	10.9	(10.1-11.8)		952	10.7	(9.8-11.6)		129	14.3	(11.4-17.6)		64	9.4	(6.6-12.9)		24	12.4	(7.3-19.1)	
	**2007-2011**	1308	11.7	(10.9-12.5)		1059	11.2	(10.4-12.1)		145	15.6	(12.6-18.8)		73	11.3	(8.2-14.9)		30	13.3	(8.1-19.9)	
	**2008-2012**	1488	12.7	(11.9-13.5)		1208	12.1	(11.3-13)		162	15.9	(13-19.1)		86	14.3	(10.9-18.2)		34	16.2	(10.2-23.6)	
	**2009-2013**	1694	13.4	(12.6-14.3)		1359	12.9	(12-13.8)		195	17.6	(14.6-20.7)					ShortFU	42	16.9	(11-24)	
	**Total Change***		5.8				5.2				11.3				4.3			NC			

HCC		UK				England				Scotland				Wales				Northern Ireland			
	Cohort	N	Net Survival	CI	suppress	N	Net Survival	CI	suppress	N	Net Survival	CI	suppress	N	Net Survival	CI	suppress	N	Net Survival	CI	suppres
**Persons**	**1997-2001**	675	9.4	(8.5-10.4)		552	9.3	(8.2-10.3)		80	10.8	(8.1-14.1)		27	8.7	(5.3-13.2)					<10
	**1998-2002**	754	9.9	(9-10.8)		622	9.7	(8.7-10.8)		84	10.7	(8-13.9)		31	10.2	(6.6-14.8)		12	6.2	(2.4-12.7)	
	**1999-2003**	847	10.5	(9.6-11.4)		697	10.4	(9.4-11.5)		91	10.2	(7.6-13.2)		36	12	(8.1-16.8)		18	6.6	(2.5-13.6)	
	**2000-2004**	914	10.7	(9.9-11.7)		740	10.6	(9.6-11.6)		108	11.0	(8.6-13.9)		42	12.3	(8.4-17.1)		20	10.6	(5-18.9)	
	**2001-2005**	984	10.6	(9.7-11.5)		796	10.5	(9.6-11.5)		121	10.9	(8.5-13.7)		44	11.3	(7.7-15.8)		21	9.7	(4.7-17.5)	
	**2002-2006**	1123	11.0	(10.2-11.9)		911	11	(10.1-12)		134	11.2	(8.9-13.8)		50	10.6	(7.1-14.9)		27	11.7	(6.3-19.2)	
	**2003-2007**	1281	11.7	(10.8-12.5)		1025	11.5	(10.6-12.5)		162	12.7	(10.4-15.3)		57	11.3	(7.9-15.4)		36	10.9	(6-17.6)	
	**2004-2008**	1473	12.3	(11.5-13.2)		1169	12.3	(11.4-13.2)		206	13.7	(11.4-16.3)		68	10.5	(7.5-14.1)		33	9.4	(5.3-14.9)	
	**2005-2009**	1722	13.5	(12.7-14.3)		1357	13.3	(12.4-14.3)		243	16.3	(13.9-18.9)		88	13.1	(9.9-16.8)		40	7.5	(4.2-12.2)	
	**2006-2010**	2014	14.8	(14-15.7)		1574	14.6	(13.7-15.5)		281	17.7	(15.3-20.3)		110	15.1	(11.8-18.9)		51	10.6	(6.8-15.5)	
	**2007-2011**	2314	15.9	(15.1-16.8)		1771	15.4	(14.5-16.3)		343	19.8	(17.4-22.4)		142	17.3	(14-21)		59	12.7	(8.6-17.7)	
	**2008-2012**	2709	17.2	(16.4-18)		2051	16.7	(15.8-17.6)		424	21.4	(19-24)		169	17.2	(14-20.7)		70	15.5	(11.1-20.6)	
	**2009-2013**	3172	18.3	(17.5-19.1)		2386	17.6	(16.7-18.5)		514	23.9	(21.5-26.4)					ShortFU	88	16.5	(12.2-21.4)	
	**Total Change***		8.9				8.3				13.1				8.5				NC		
**Males**	**1997-2001**	480	8.8	(7.7-9.9)		392	8.5	(7.3-9.7)		63	11.2	(7.9-15.2)		16	8.3	(4.4-14)					<10
	**1998-2002**	547	9.4	(8.4-10.5)		449	9.2	(8.1-10.4)		67	11.2	(8-15.1)		20	10	(5.7-15.7)					<10
	**1999-2003**	612	9.8	(8.8-10.9)		504	9.6	(8.5-10.7)		70	10.7	(7.7-14.2)		24	12.3	(7.7-18.3)					<10
	**2000-2004**	667	10.1	(9.1-11.1)		546	9.9	(8.8-11.1)		77	10.1	(7.4-13.4)		28	10.8	(6.5-16.4)		11	10.7	(4.5-20.6)	
	**2001-2005**	726	10.0	(9.1-11)		593	10	(9-11.1)		88	9.8	(7.2-12.9)		30	10.3	(6.2-15.8)		12	8.2	(3-17.4)	
	**2002-2006**	836	10.6	(9.7-11.6)		684	10.7	(9.6-11.8)		96	10.1	(7.6-13)		37	10.1	(6.1-15.3)		17	9.4	(4.1-17.9)	
	**2003-2007**	957	11.0	(10.1-12)		768	10.9	(9.9-12)		118	11.8	(9.3-14.8)		44	11.7	(7.7-16.8)		26	9.1	(4.2-16.5)	
	**2004-2008**	1121	12.0	(11.1-12.9)		889	11.9	(10.8-12.9)		155	13.3	(10.7-16.3)		55	11.4	(7.7-15.9)		24	7.9	(3.7-14.4)	
	**2005-2009**	1330	13.2	(12.3-14.2)		1039	12.9	(11.9-14)		187	16.3	(13.5-19.3)		74	14.7	(10.8-19.3)		32	6.4	(2.9-11.8)	
	**2006-2010**	1566	14.7	(13.8-15.7)		1221	14.4	(13.3-15.4)		214	17.2	(14.5-20.2)		90	17.2	(13.1-21.9)		42	9.7	(5.6-15.4)	
	**2007-2011**	1788	15.7	(14.7-16.6)		1355	14.9	(13.9-16)		268	19.2	(16.5-22.2)		116	19.2	(15.2-23.6)		50	12.9	(8.3-18.8)	
	**2008-2012**	2092	16.8	(15.8-17.7)		1574	16.2	(15.2-17.2)		331	20.7	(17.9-23.6)		133	17.1	(13.5-21.1)		56	15.3	(10.4-21.3)	
	**2009-2013**	2433	17.7	(16.8-18.6)		1824	17.2	(16.2-18.2)		397	22.6	(19.9-25.4)					ShortFU	70	16.7	(11.8-22.5)	
	**Total Change***		8.9				8.7				11.4				8.8				NC		
**Females**	**1997-2001**	201	11.8	(9.9-13.9)		164	11.9	(9.8-14.3)		19	10.7	(6-17.2)		11	8.8	(3.4-17.5)					<10
	**1998-2002**	211	11.4	(9.5-13.4)		175	11.6	(9.6-13.9)		19	9.9	(5.3-16.3)		10	8.6	(3.5-16.8)					<10
	**1999-2003**	241	12.8	(10.9-14.9)		198	13.5	(11.3-15.8)		20	8.4	(4.3-14.3)		12	10.8	(4.8-19.5)					<10
	**2000-2004**	255	13.4	(11.5-15.5)		202	13.3	(11.2-15.7)		31	14.0	(8.8-20.5)		14	16.5	(8.8-26.6)					<10
	**2001-2005**	267	12.7	(10.9-14.7)		211	12.4	(10.4-14.6)		34	15.3	(10-21.8)		14	13.6	(7.2-22.4)		10	18.2	(6.3-36.9)	
	**2002-2006**	294	12.6	(10.8-14.5)		232	12.2	(10.3-14.4)		39	14.9	(9.9-20.9)		13	11.7	(5.8-19.9)		11	19.8	(7.6-37.9)	
	**2003-2007**	332	13.9	(12.1-15.9)		264	13.8	(11.8-16)		46	16.4	(11.4-22.4)		13	11.1	(5.5-18.9)					<10
	**2004-2008**	356	13.6	(11.8-15.4)		284	13.9	(12-16.1)		53	15.4	(10.7-21)		14	9.3	(4.6-16)					<10
	**2005-2009**	398	14.5	(12.8-16.3)		322	14.9	(13-17)		58	17.8	(12.8-23.4)		17	11.8	(6.4-19)					<10
	**2006-2010**	452	15.4	(13.7-17.2)		356	15.4	(13.5-17.5)		69	19.6	(14.5-25.3)		21	9.5	(4.8-16.3)					<10
	**2007-2011**	530	17.1	(15.4-18.8)		417	17	(15.1-19)		78	22.1	(16.9-27.8)		27	11.7	(6.5-18.6)					<10
	**2008-2012**	618	18.8	(17.1-20.6)		477	18.5	(16.6-20.5)		94	22.9	(17.8-28.5)		37	18.3	(11.8-26.1)		12	15.3	(7.3-26.5)	
	**2009-2013**	744	20.2	(18.5-21.9)		564	19.1	(17.3-21)		118	28.1	(22.6-33.8)					ShortFU	19	17.7	(9.5-28.2)	
	**Total Change***		8.4				7.2				17.4				9.5				NC		

ICCA		UK				England				Scotland				Wales				Northern Ireland			
	**Cohort**	**N**	**Net Survival**	**CI**	**suppress**	**N**	**Net Survival**	**CI**	**suppress**	**N**	**Net Survival**	**CI**	**suppress**	**N**	**Net Survival**	**CI**	**suppress**	**N**	**Net Survival**	**CI**	**suppres**
**Persons**	**1997-2001**	464	4.4	(3.8-5.1)		367	4.2	(3.5-5)		53	4.6	(2.8-7.2)		46	9.8	(6.3-14.2)					<10
	**1998-2002**	492	4.2	(3.6-4.9)		397	4.3	(3.6-5)		53	3.2	(1.7-5.4)		39	6.5	(3.8-10.3)					SE>0.2
	**1999-2003**	524	4.7	(4-5.4)		423	4.7	(4-5.4)		55	3.7	(2.1-6.1)		43	7.2	(4.4-10.9)					SE>0.2
	**2000-2004**	550	4.8	(4.2-5.5)		442	4.9	(4.2-5.6)		61	4.5	(2.7-6.9)		44	6.3	(3.8-9.8)					SE>0.2
	**2001-2005**	579	4.9	(4.3-5.6)		465	4.7	(4.1-5.4)		63	5.8	(3.8-8.5)		46	7.2	(4.5-10.7)					<10
	**2002-2006**	631	5.1	(4.5-5.7)		509	4.9	(4.3-5.6)		63	6.3	(4.2-9)		49	7.6	(4.9-11.1)					<10
	**2003-2007**	673	5.4	(4.8-6.1)		539	5.2	(4.5-5.9)		74	7.6	(5.3-10.3)		56	7.3	(4.7-10.5)					<10
	**2004-2008**	732	5.3	(4.7-5.9)		588	5.1	(4.5-5.8)		83	7.8	(5.6-10.6)		57	5.8	(3.7-8.6)					<10
	**2005-2009**	831	5.7	(5.1-6.3)		669	5.4	(4.8-6.1)		90	8.1	(5.8-10.9)		65	6.9	(4.6-9.8)		13	4.9	(1.8-10.4)	
	**2006-2010**	878	5.9	(5.3-6.5)		713	5.7	(5.1-6.4)		94	7.9	(5.7-10.6)		68	7.6	(5.2-10.6)		11	5	(1.8-10.8)	
	**2007-2011**	950	6.0	(5.5-6.7)		782	5.9	(5.3-6.6)		94	7.6	(5.4-10.3)		57	6.5	(4.3-9.3)		16	5.7	(2.1-12)	
	**2008-2012**	1014	6.0	(5.5-6.6)		838	5.8	(5.2-6.5)		97	6.7	(4.7-9.1)		61	8	(5.6-10.9)		18	6.6	(2.7-12.9)	
	**2009-2013**	1088	6.1	(5.6-6.7)		890	6	(5.4-6.7)		108	5.2	(3.6-7.2)					ShortFU	21	7.8	(3.6-14.2)	
	**Total Change***		1.7				1.8				0.6				-1.8				NC		
**Males**	**1997-2001**	205	4.3	(3.4-5.4)		169	3.9	(3-5)		21	7.5	(3.9-12.8)		13	8	(3.4-15.4)					Insuff
	**1998-2002**	209	3.9	(3.1-4.9)		176	3.7	(2.8-4.8)		18	4.8	(2-9.5)		15	8.6	(3.9-15.8)					Insuff
	**1999-2003**	230	4.2	(3.4-5.3)		196	4.1	(3.2-5.2)		16	3.2	(1.1-7.4)		17	8.5	(4.1-15.2)					Insuff
	**2000-2004**	247	4.5	(3.6-5.5)		210	4.5	(3.5-5.6)		17	2.1	(0.6-5.6)		19	8.5	(4.2-14.9)					Insuff
	**2001-2005**	263	4.5	(3.6-5.4)		218	4.2	(3.3-5.2)		21	2.6	(0.9-6.2)		22	10.2	(5.5-16.9)					Insuff
	**2002-2006**	288	4.9	(4-5.9)		236	4.6	(3.7-5.6)		28	5.1	(2.5-9)		22	11	(6.2-17.6)					<10
	**2003-2007**	322	5.6	(4.7-6.6)		261	5.3	(4.3-6.3)		34	5.8	(3.1-9.7)		25	11.8	(7-18.1)					<10
	**2004-2008**	353	5.7	(4.8-6.7)		287	5.5	(4.5-6.5)		39	6.9	(3.9-11.1)		29	9.1	(5.2-14.5)					<10
	**2005-2009**	386	5.8	(4.9-6.8)		309	5.5	(4.6-6.5)		45	7.2	(4.2-11.5)		30	9	(5.3-14.1)					<10
	**2006-2010**	415	6.2	(5.3-7.2)		343	6.2	(5.2-7.2)		42	6.2	(3.5-10.1)		35	10	(6.1-15.2)					<10
	**2007-2011**	443	6.3	(5.4-7.2)		375	6.5	(5.6-7.5)		37	4.7	(2.3-8.4)		26	4.5	(2.1-8.5)					<10
	**2008-2012**	461	6.0	(5.2-6.9)		384	6.1	(5.2-7.1)		42	4.5	(2.3-7.9)		28	8.7	(5.3-13.4)					<10
	**2009-2013**	500	6.3	(5.5-7.2)		416	6.4	(5.5-7.4)		47	3.7	(1.9-6.7)					ShortFU				<10
	**Total Change***		2.0				2.5				-3.8				0.7				NC		
**Females**	**1997-2001**	269	5.0	(4.1-6)		204	5.2	(4.2-6.4)		33	2.6	(1.1-5.4)		32	11.3	(6.6-17.6)					<10
	**1998-2002**	290	4.7	(3.9-5.7)		225	5.2	(4.2-6.4)		38	2.4	(1-5.1)		24	5.1	(2.2-9.8)					SE>0.2
	**1999-2003**	303	5.4	(4.5-6.4)		231	5.6	(4.6-6.8)		40	4.4	(2.2-7.7)		25	4.5	(1.9-8.9)					<10
	**2000-2004**	307	5.3	(4.4-6.3)		232	5.4	(4.5-6.5)		47	6.5	(3.8-10.1)		23	3.4	(1.3-7.3)					<10
	**2001-2005**	320	5.4	(4.6-6.4)		248	5.3	(4.4-6.4)		46	9.0	(5.8-13.2)		21	3.2	(1.2-6.8)					<10
	**2002-2006**	344	5.3	(4.5-6.2)		273	5.3	(4.4-6.3)		38	8.5	(5.3-12.7)		25	4.2	(1.9-8.1)					<10
	**2003-2007**	349	5.2	(4.4-6.1)		276	5.1	(4.2-6)		40	9.3	(6-13.5)		27	2.8	(1.1-6.1)					<10
	**2004-2008**	377	4.9	(4.1-5.7)		299	4.7	(3.9-5.6)		43	8.2	(5.2-12.1)		27	2.9	(1.2-6)					<10
	**2005-2009**	449	5.6	(4.8-6.4)		361	5.3	(4.5-6.3)		45	8.9	(5.8-12.9)		36	5.2	(2.7-8.8)					<10
	**2006-2010**	464	5.6	(4.8-6.4)		367	5.2	(4.4-6.1)		52	9.1	(5.9-13.1)		35	6	(3.3-9.9)					<10
	**2007-2011**	507	5.8	(5-6.6)		405	5.3	(4.5-6.1)		58	9.7	(6.5-13.6)		35	8.9	(5.5-13.3)		12	4.3	(0.7-13.5)	
	**2008-2012**	558	6.1	(5.3-6.9)		456	5.6	(4.8-6.5)		55	8.6	(5.8-12.2)		36	8.9	(5.6-13.2)		11	4.2	(0.6-14.2)	
	**2009-2013**	594	6.0	(5.2-6.8)		477	5.7	(4.9-6.6)		60	6.3	(4-9.3)					ShortFU	13	7.7	(2.1-18.6)	
	**Total Change***		1.0				0.5				3.7				-2.4				NC		

Other		UK				England				Scotland				Wales				Northern Ireland			
	**Cohort**	**N**	**Net Survival**	**CI**	**suppress**	**N**	**Net Survival**	**CI**	**suppress**	**N**	**Net Survival**	**CI**	**suppress**	**N**	**Net Survival**	**CI**	**suppress**	**N**	**Net Survival**	**CI**	**suppres**
**Persons**	**1997-2001**	128	5.6	(4.5-7)		111	5.3	(4.2-6.8)					<10	10	9.7						SE>0.2
	**1998-2002**	138	6.4	(5.1-7.8)		118	5.8	(4.5-7.3)					<10	13	14.2						Insuff
	**1999-2003**	147	6.8	(5.5-8.3)		123	6	(4.7-7.5)					<10	17	17.9						Insuff
	**2000-2004**	138	6.3	(5-7.8)		113	5.3	(4-6.8)					<10	16	20						<10
	**2001-2005**	156	7.2	(5.8-8.9)		132	6.4	(5-8.1)					<10	13	17.3						<10
	**2002-2006**	185	9.3	(7.7-11.1)		164	9.1	(7.4-11)					<10				<10				Insuff
	**2003-2007**	202	10.0	(8.4-11.8)		183	10.2	(8.4-12.2)					<10				<10				Insuff
	**2004-2008**	255	10.7	(9.1-12.5)		239	11.2	(9.5-13.2)					<10				<10				Insuff
	**2005-2009**	353	13.9	(12.2-15.8)		332	14.4	(12.6-16.4)					<10				<10				Insuff
	**2006-2010**	439	16.0	(14.2-17.8)		413	16.5	(14.6-18.4)					<10				<10				<10
	**2007-2011**	475	15.4	(13.7-17.2)		437	15.8	(14-17.7)					<10	15	23.6			13	17.4	(8.8-28.8)	
	**2008-2012**	544	15.7	(14.2-17.4)		503	16.1	(14.4-17.8)					SE>0.2	19	23.3			13	17.9	(9.1-29.5)	
	**2009-2013**	651	17.3	(15.8-19)		607	17.7	(16-19.4)					<10				ShortFU	13	19.2	(10-31)	
	**Total Change***		11.7				12.4				NC				NC				NC		
**Males**	**1997-2001**	60	4.9	(3.6-6.6)		51	4.4	(3.1-6.2)					<10				<10				Insuff
	**1998-2002**	66	5.9	(4.3-7.7)		54	5	(3.5-6.9)					<10				<10				Insuff
	**1999-2003**	68	6.4	(4.8-8.3)		53	5.2	(3.7-7.1)					<10	13	21.6	(11.5-34.4)					Insuff
	**2000-2004**	66	6.3	(4.6-8.3)		52	5	(3.5-7)					<10	11	22.4	(11.2-37.1)					Insuff
	**2001-2005**	73	7.0	(5.2-9.1)		61	6.2	(4.4-8.4)					<10				<10				<10
	**2002-2006**	88	8.7	(6.7-11.1)		77	8.4	(6.2-11)					<10				<10				Insuff
	**2003-2007**	103	9.5	(7.4-11.9)		94	9.6	(7.3-12.2)					Insuff				<10				Insuff
	**2004-2008**	132	9.5	(7.5-11.8)		125	10	(7.8-12.5)					Insuff				<10				Insuff
	**2005-2009**	172	11.5	(9.4-13.9)		163	12	(9.7-14.6)					Insuff				<10				Insuff
	**2006-2010**	227	14.0	(11.8-16.4)		218	14.9	(12.5-17.4)					Insuff				Insuff				<10
	**2007-2011**	248	12.9	(10.9-15.1)		235	13.7	(11.5-16.1)					SE>0.2				Insuff				<10
	**2008-2012**	292	13.8	(11.8-15.9)		275	14.6	(12.5-16.9)					SE>0.2				Insuff				<10
	**2009-2013**	362	16.0	(14-18.1)		342	16.8	(14.7-19.1)					SE>0.2				Insuff				<10
	**Total Change***		11.1				12.4				NC				NC				NC		
**Females**	**1997-2001**	72	6.7	(4.8-9)		65	6.9	(4.8-9.4)					<10				<10				Insuff
	**1998-2002**	76	7.0	(5-9.4)		69	6.9	(4.8-9.5)					<10				<10				Insuff
	**1999-2003**	83	7.4	(5.4-9.9)		73	6.9	(4.8-9.4)					<10				<10				Insuff
	**2000-2004**	74	6.1	(4.2-8.4)		64	5.6	(3.7-8)					<10				Insuff				Insuff
	**2001-2005**	83	7.4	(5.4-10)		71	6.6	(4.6-9.3)					<10				Insuff				Insuff
	**2002-2006**	97	10.1	(7.6-12.9)		87	10.1	(7.5-13.1)					<10				Insuff				Insuff
	**2003-2007**	99	11.2	(8.7-14.1)		91	11.7	(8.9-14.9)					SE>0.2				Insuff				Insuff
	**2004-2008**	124	12.6	(10-15.5)		115	13.3	(10.4-16.4)					SE>0.2				Insuff				Insuff
	**2005-2009**	184	17.9	(15-21)		171	18.4	(15.3-21.7)					<10				Insuff				Insuff
	**2006-2010**	217	19.5	(16.6-22.5)		199	19.5	(16.5-22.7)					Insuff				<10				Insuff
	**2007-2011**	230	19.5	(16.7-22.5)		204	19.2	(16.3-22.4)					Insuff	10	30.5	(14.9-48.7)					Insuff
	**2008-2012**	254	18.8	(16.2-21.5)		229	18.3	(15.6-21.1)					Insuff	11	35.9	(18.3-55.5)					<10
	**2009-2013**	287	19.1	(16.7-21.7)		262	18.7	(16.2-21.4)					Insuff				Insuff				<10
	**Total Change***		12.4				11.8				NC				NC				NC		

*Between 1997-2001 and 2009-2013 (2008-2012 for Wales). Suppressed results: Insuff, no deaths or data in at least one age band; <10, less than 10 cases in total; SE>0.2, standard error greater than 0.2; 2yr>1yr, NC: Not Calculatable

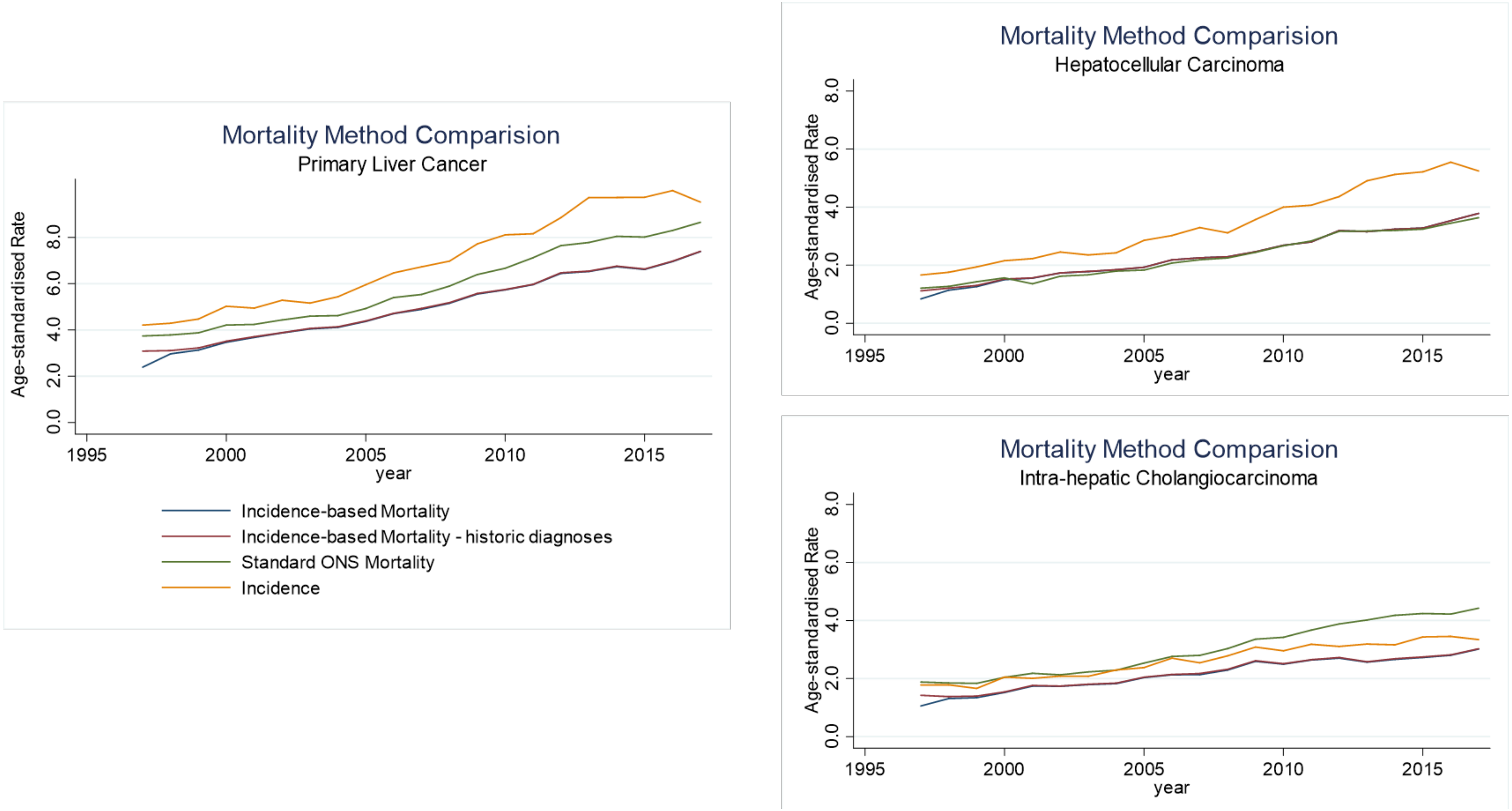

Fig. S1: Sensitivity analysis: standard mortality vs incidence-based mortality in English data

Note: Historic diagnoses are diagnoses pre-1997

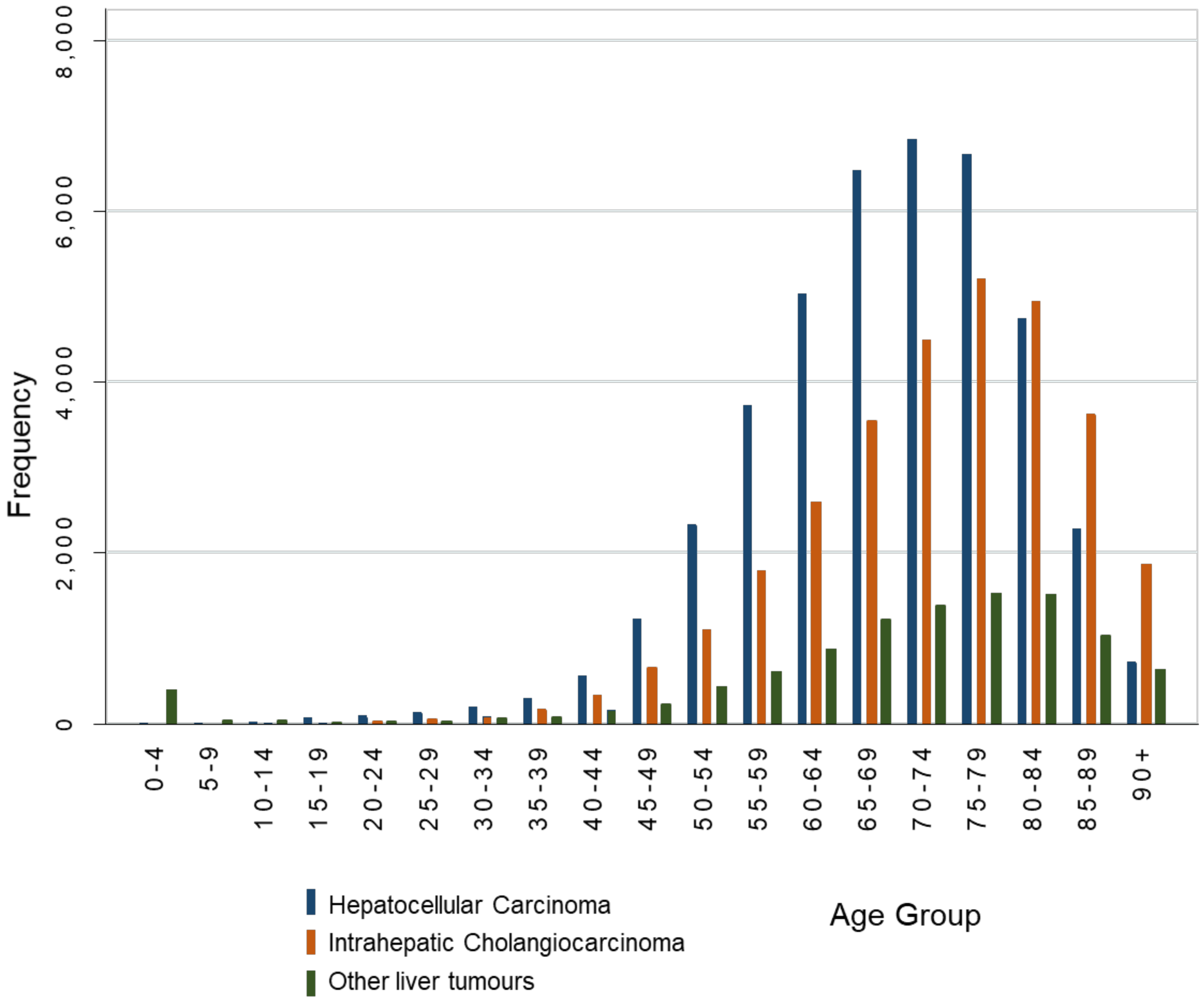

Fig. S2: Age distribution by subtype

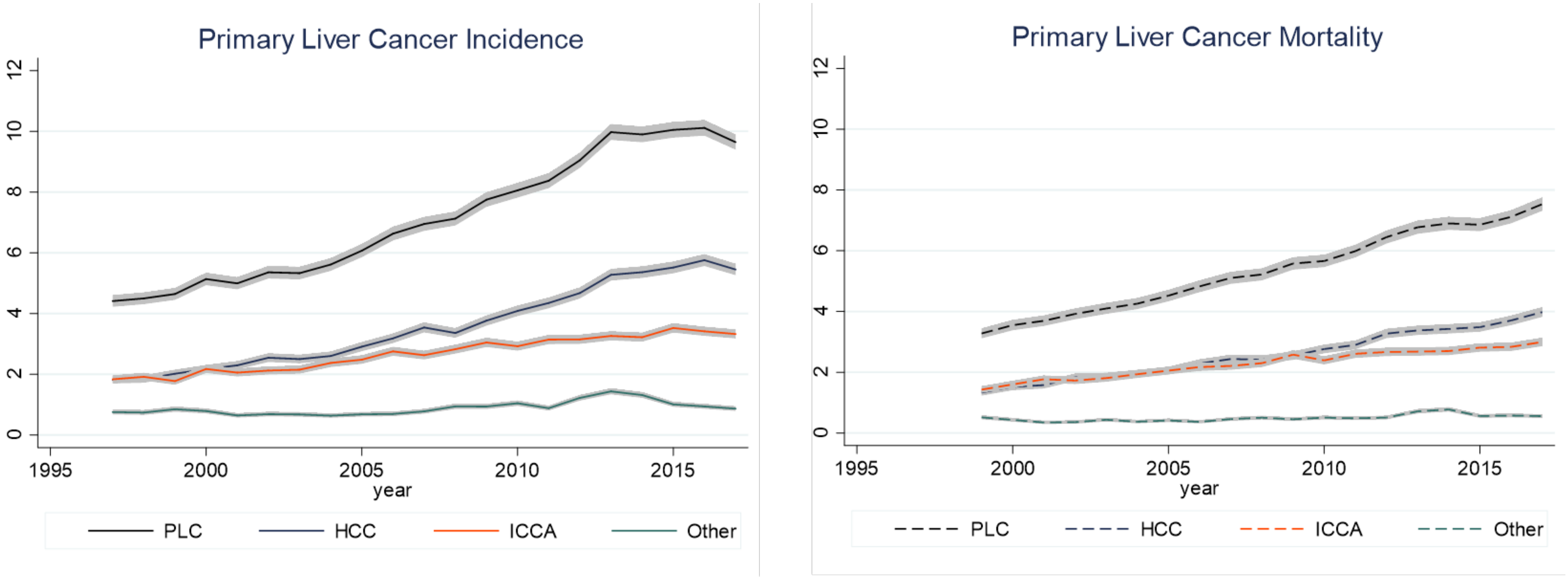

Fig. S3: Primary liver cancer age-standardised incidence and incidence-based mortality 1997-2017)

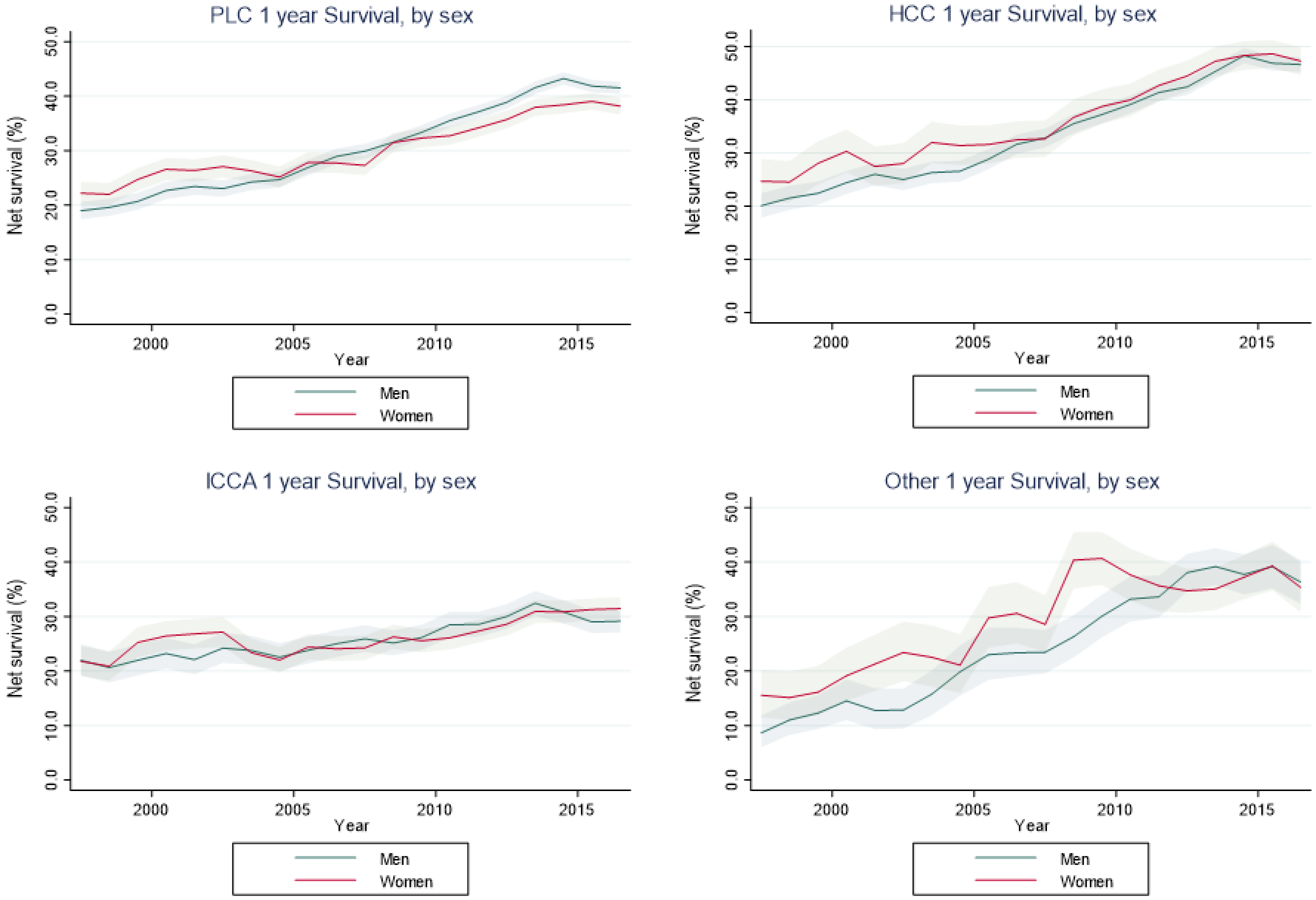

Fig. S4: Net-survival by sex and subtype

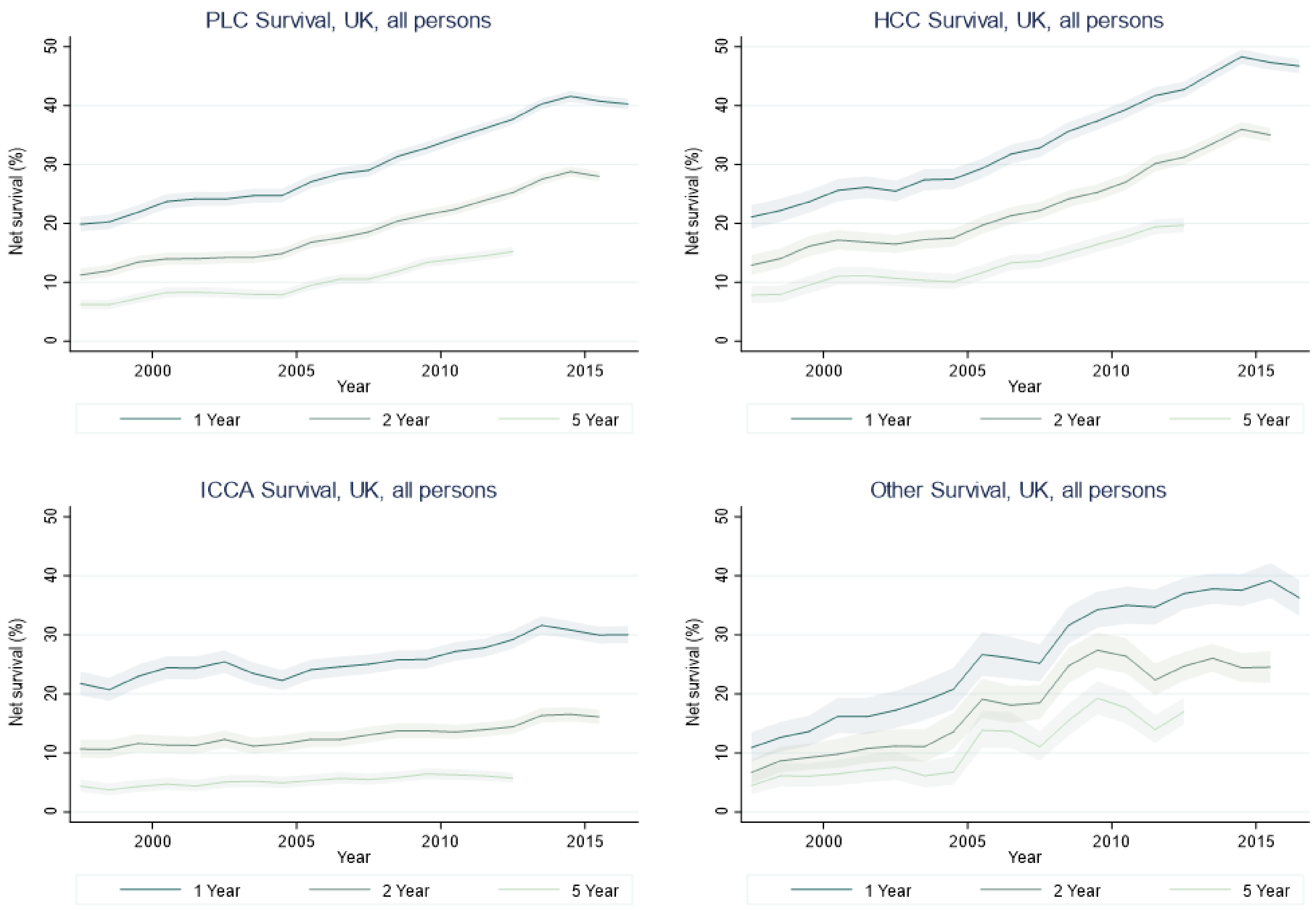

Fig. S5: Net-survival by subtype
